# Supplementary material for: Systematic evaluation of isoform function in literature reports of alternative splicing
Source: BMC Genomics. 2018 Aug 28;19:637. doi: 10.1186/s12864-018-5013-2 (PMC6114036; doi:10.1186/s12864-018-5013-2)
Supplement: Supplementary file 1 — Contains explanations of each heading found in Additional file 2, standards curators used to evaluate studies for evidence of functionally distinct splice isoforms, three genes where literature reported splice isoforms were not found in Ensembl, supplemental tables and figures, and citations for all human and mouse literature curated for this study. (PDF 1624 kb) [file 12864_2018_5013_MOESM1_ESM.pdf]

Systematic evaluation of isoform function in literature reports of alternative splicing: Supplemental Data

Authors: Shamsuddin A. Bhuiyan[1,2], Sophia Ly[1], Minh Phan[1], Brandon Huntington[1], Ellie Hogan[1], Chao Chun Liu[1], James Liu[1], Paul Pavlidis*[1,2]

[1] Michael Smith Laboratories, University of British Columbia, Vancouver, BC V6T 1Z4, Canada
[2] Department of Psychiatry, University of British Columbia, Vancouver, BC V6T 1Z4, Canada

*To whom correspondence should be addressed. Tel: (604) 827-4157 Email: paul@msl.ubc.ca

Table of Contents

SECTION 1: MASTER SPREADSHEET LEGEND 3

SECTION 2: CURATION STANDARDS (INSTRUCTIONS TO FILL OUT MASTER SPREADSHEET) 5

SECTION 3: PROBLEMATIC CASES OF LINKING FDSIs TO ENSEMBL 8

RNA binding protein, fox-1 homolog 1 (*Rbfox1*) 8

Ryanodine receptor 3 (*Ryr3*) 9

Sad1 and UNC84 domain containing 1 (*SUN1*) 9

SECTION 4: SUPPLEMENTAL TABLES AND FIGURES 11

Section 5: References for all curated studies 15

SECTION 1: MASTER SPREADSHEET LEGEND

File S2 contains all human and mouse studies we curated along with all information annotated for each study. In this section, we explain each column heading in File S2. Guidelines to adding publications to this spreadsheet can be found in Section 2.

- "Evidence_of_Functional_Distinctness" – This column indicates whether the study provided evidence of functional distinctness. The column is annotated as the following (for further information, see Methods and Figure 2):
    - If no depletion of a splice isoform occurred, then this column is left blank
    - "Positive" – If multiple isoforms of the same gene are individually depleted and more than two individual depletions cause a phenotype
    - "Potentially positive" – If the depletion of a single isoform causes a phenotype
    - "Negative" – If the depletion of the multiple isoforms of the same gene occurs but only one depletion causes a phenotype while the remaining depletions cause no isoform
- "Gene" – the gene symbol and gene name investigated in this study
- "PMID" – the PubMed identifier of the curated study
- "Isoform_Names" – a list of the isoforms studied in the curated study
    - "NA" – Study will be annotated as NA if the study does not investigate splice isoforms
- "Number_of_Reported_Isoforms" – the number of splice isoforms for the investigated gene according to the authors
    - "NA" – Study will be annotated as NA if the study does not investigate splice isoforms
- "Number_of_Functional_Isoforms_according_to_authors" – the number of splice isoforms for the investigated gene that the authors claim is functional
    - "NA" – Study will be annotated as NA if the study not investigate splice isoforms or the authors make no claim about how many are functional
- "Article_claims_functional_splice_isoforms" – whether the authors indicate that the splice isoforms of the investigated gene are functional
    - "NA" – Study will be annotated as NA if the study does not investigate splice isoforms, or if the study makes no claims on functionality
- "Evidence_of_Functionality" – curator's notes about how functionality was tested in this paper
- "Isoform_causes_disease" – the investigated splice isoforms are only expressed in disease conditions
- "Isoform_organism" – the organism that endogenously expresses the splice isoforms
- "Experimental_organism" – the organism the splice isoforms were tested in
- "Only_evidence_is_presence" – a "no" in this column indicates depletion experiments in the study
- "Type_of_experiments" – experiments used to characterize splice isoforms

- “Curator” – initials of individual who curated the study

SECTION 2: CURATION STANDARDS (INSTRUCTIONS TO FILL OUT MASTER SPREADSHEET)

1. Double check the Master Spreadsheet to ensure the paper you have curated has not already been curated.
2. If paper is a review article, fill in all column with NAs with the exception of study type and PubMed ID. Fill in Pubmed ID with PubMed ID and study type with “review article”
3. Note that even if the species is not of interest (i.e human or mouse), we still will annotate the experiment
4. Identify investigated gene(s). Fill out gene column with the NCBI gene name and PMID with the article’s PubMed ID.
    a. If multiple genes are investigated, then one row per gene
    b. If a paper doesn’t have a PMID, enter in the citation for the paper
    c. The gene name should be written to the standards of the organism in which the isoforms are endogenously expressed
5. Identify the number of splice isoforms for the investigated gene. Identify the names of the splice isoforms if possible.
    a. This is reported by the authors but may be different from the number of splice isoforms they actually test in the experiment
    b. Fill out ‘# of splice isoforms’ column and ‘isoform name” column
6. Identify if the paper is actually about identifying the function of the gene’s **endogenously** expressed splice isoforms.
    a. Splicing papers are sometimes not about the gene’s splice isoform’s function but regulation of the alternative splicing of that gene.
        i. This can be further confusing if the investigated gene is a splicing regulator. Sometimes these splicing regulators have splice isoforms whose function is being studied.
    b. If the study is about the regulation of the splice isoforms and not function, then make a note of this in the Evidence of Functionality column. Fill out the remaining columns as NA. Stop curating the study.
    c. Endogenously expressed isoforms are ones that are expressed in a healthy wildtype population
        i. Splice isoforms expressed solely in a disease condition is **not** what we are looking for.
            1. Example: in cancer we observe novel splice isoform expressions and we don’t report on this
            2. If the study is about disease associated splice isoforms, make a note of this in the ‘Evidence of Functionality’ column and mark the “isoform

causes a disease" column as yes. Fill out the remaining columns as NA and then stop curating. Annotate "study type" column as "disease association"

d. This can be further complicated if the **upregulation of an endongenously expressed splice isoform** causes a disease. Certain studies can identify a splice isoform that is necessary for the overall function of the gene at the wildtype expression level but at an upregulated level, there's a disease phenotype. We are still interested in these splice isoforms.
    i. Another source of confusion is that often time a lowly expressed wildtype splice isoform can be slightly detrimental but has no effect on the cell as it is lowly expressed. But when the splice isoform is upregulated or an overexpression study, a disease is caused because now the splice isoform is abundant.

7. Determine which splice isoforms the authors use for their experiments and whether or not the authors are claiming the splice isoforms are functional
    a. Fill out the "# of functional isoforms" column
8. Determine the organism the splice isoform has been studied in and fill out the "Organism" column
    a. This is generally the organism where the splice isoform is endogenously expressed
        i. However, studies will occasionally test their splice isoforms in multiple organisms.
        ii. Add this to the organism column (e.g mouse, rat, ferret)
9. In the 'study types' column, fill out the experiment that was performed to investigate the splice isoforms. Common experiments we have seen are: knockdown, knockdown (1 isoform), knockdown (non-isoform-specific), knockout, knockout (1 isoform), knockout (non-isoform-specific), rescue, rescue (1-isoform), rescue (non-isoform-specific) overexpression, tissue distribution, subcellular localization, activity assay, protein interaction, disease association, regulation of isoform expression, detection, structural characterization, mutation isoform absence, immunodepletion, timecourse distribution
    a. If multiple experiments were used, then annotate column with all experiments. The main experiment type should be listed first.
        i. Usually if the experiments depleted a splice isoform, then we want that experiment listed first.
            1. Example: if an experiment investigated isoform tissue distribution and knockdown, then "knockdown, tissue distribution" is what the column should be annotated with
        ii. If any of the experiments used eliminated the expression of a single isoform and looked for an effect, then mark the 'Only evidence is presence' as no. These are the studies we are most interested in.

iii. If more than one splice isoform is shown to be necessary (i.e the absence of each splice isoform causes a phenotype) then mark this study in the “Evidence for Functional Distinctness?” column as “positive”.
iv. If only one splice isoform is depleted, then mark this study in the “Evidence for Functional Distinctness column as “potentially positive”

b. If the splice isoforms cause a different function from each other or have the same function as each other, then fill out the appropriate column to reflect that (“same function” column or “different function” column)

10. Fill out the Evidence of Functionality column with a short, concise description of the study in present tense. This description will often explain how the isoforms were molecularly characterized and what function was tested. If the paper does provide evidence of a “Gold Standard Gene”, please include in the description which figure(s) best shows this evidence.
11. Fill out “sequence accession” column with any sequence accession information provided to the investigated splice isoforms.

SECTION 3: PROBLEMATIC CASES OF LINKING FDSIs TO ENSEMBL

The following file describes the functionally distinct splice isoforms (FDSIs) that we failed to link to an appropriate Ensembl Transcript ID, and the investigators of the curated literature provided sequence accessions.

RNA binding protein, fox-1 homolog 1 (*Rbfox1*)

According to Hamada and colleagues, mouse *Rbfox1* has two FDSIs: Rbfox1-isoform5 (aliases: Rbfox1_C, RBFOX1-isoform2) and Rbfox1-isoform1 (alias: Rbfox1_N) (1). Hamada and colleagues performed knockdown experiments of both splice isoforms. In vivo knockdown of Rbfox1-isoform1 in mice resulted in cortical neuron radial migration and terminal translocation defects. In vitro knockdown of Rbfox1-isoform1 in hippocampal neurons reduced spine density, and the length of dendrites and primary axons. In-vitro Rbfox1-isoform5 knockdown increased the number of stubby-shaped immature spines only, while Rbfox1-isoform1 knockdown increased the number of filopodia-like immature spines as well.

The Ensembl database contains two splice transcripts for mouse *Rbfox1*: ENSMUST00000056416.7 and ENSMUST00000115841.9. The transcript ENSMUST00000056416.7 has a CDS length of 4048 bp and a protein length of 417 amino acids. The other transcript, ENSMUST00000115841.9, has a CDS length of 1,192 bp and a protein length of 396 amino acids.

We linked Rbfox1-isoform1 to Ensembl transcript ID ENSMUST00000115841.9 for Rbfox1. Hamada and colleagues constructed the plasmid for Rbfox1-isoform1 from GenBank submission AY659954. According to GenBank, Rbfox1-isoform1 has a CDS length of 1188 bp and a protein length of 396 amino acids. The amino acid sequence found in GenBank for Rbfox1-isoform1 match exactly to the amino acid sequence for an Ensembl transcript ID ENSMUST00000115841.9 – though the CDS sequences do not match.

We failed to link Rbfox1-isoform5 to any Ensembl transcripts. Hamada and colleagues constructed the plasmid for this splice isoform from the sequence reported in the GenBank entry AY659955. According to this GenBank record, Rfox1-isoform5 has a CDS length of 1,241 bp and protein length of 373 amino acids which does not match either transcript reported in the Ensembl entry for Rbfox1.

Two other reports further provided positive evidence of FDSIs for *Rbfox1*. Hamada and colleagues (2015) again performed in vitro knockdown experiments for Rfox1-isoform5 (2). Lee and colleagues performed isoform specific rescues using Rbfox1-isoform1 and Rbfox-isoform5 (3). Neither reports provided GenBank accessions however they provided sequence information which matched the isoforms reported in Hamada and colleagues (2016).

Ryanodine receptor 3 (*Ryr3*)

According to Dabertrand and colleagues, mouse *Ryr3* has two FDSIs: RYR3L and RYR3S (4). Dabertrand and colleagues performed knockdown experiments for both FDSIs of Ryr3 in mouse duodenum myocytes. The elimination of RYR3L decreased the upstroke and amplitude velocity of caffeine-induced calcium response while the elimination of RYR3S increased the upstroke and amplitude velocity of caffeine-induced calcium response.

The Ensembl database contains 12 splice variants for mouse *Ryr3*. Of these splice variants, the six splice are protein coding. Five of the protein coding splice variants have a protein length greater than 4,000 amino acids while the remaining splice variant has a protein length of 46 amino acids.

We failed to link RYR3L to any Ensembl transcript. Dabertrand and colleagues constructed the plasmid for RYR3L from sequence data provided in the GenBank entry AF111166. The protein sequence provided in this GenBank entry has a length of 454 amino acids. Note, that the European Nucleotide Archive (ENA) has linked the sequence for this GenBank entry to two Ryr3 transcripts in Ensembl: ENSMUST00000080673 and ENSMUST00000208151. The amino acid length for the proteins encoded by these Ensembl transcripts are 4,863 amino acids and 4,834 amino acids, respectively. We failed to understand how ENA linked RYR3L to these Ensembl transcripts.

We also failed to link RYR3S to any Ensembl transcript. For their construction of the RYR3S plasmid, Dabertrand and colleagues referred to previous work by Coussin and colleagues (5). Coussin and colleagues constructed their sequence from the GenBank entry X83934. This GenBank entry connected us to a RYR3S protein sequence with a length of 288 amino acids. Despite failing to link this protein for RYR3S to any Ensembl entry, we linked the RYR3S sequence to the RefSeq entry XM_619795. However, this RefSeq removed this entry from their database due to insufficient evidence and replaced it with a new sequence.

Sad1 and UNC84 domain containing 1 (*SUN1*)

According to Nishioka and colleagues, human *SUN1* has three FDSIs: SUN1_916, SUN1_888 and SUN1_785 (6). They performed knockdown experiments for all three splice isoforms in HeLa cells as well as further knockdown experiments for SUN1_916 and SUN1_888 in MDA-MB-231 cells. The experiments revealed that all three splice isoforms were necessary for proper cell migration.

The Ensembl database contains 35 transcripts for human *SUN1* and 17 transcripts are protein-coding. The largest protein-coding transcript encodes for a splice isoform of 822 amino acids.

We linked SUN1_785 to the Ensembl transcript ID ENST00000401592.5. Nishioka and colleagues constructed the transcript from the RefSeq ID NM_001130965 and the transcript encoded for a 785

amino acid protein. The Ensembl database had already linked this RefSeq ID to ENST00000401592.5 and we ensured that the RefSeq protein matched the Ensembl protein using ClustalOmega.

We failed to link SUN1_916 and SUN1_888 to any Ensembl record. Nishioka and colleagues provided the GenBank accessions EAW87177.1 and AB648918, respectively, as the source for plasmid construction. According to their GenBank entries, SUN1_916 encoded a 916 amino acid protein while SUN1_888 encoded for a protein with 888 amino acids. No Ensembl record matched either of these transcripts or proteins. Furthermore, we found no notes on the GenBank entries to explain the absence of these splice isoforms on the Ensembl database.

SECTION 4: SUPPLEMENTAL TABLES AND FIGURES

ACTR2	CSF2RA	GPX6	MBD3L5	POLE	STC2
AGPAT2	CTSD	GRK4	MDM4	PPP1R32	SUMF1
AGTR1	CUL3	HBA1	MEG9	PRKAR1A	SUMO3
ALG3	DGKI	HMGB1P6	MGAT5B	PTGS2	SYNCRIP
ARHGEF26	DNAJB11	HMGB2	MKLN1	RABL6	TBC1D2
C1QTNF7	DPEP3	HMGB3P18	MLLT10P1	REG1A	THRB
CCDC71L	DSC3	HRH3	MORN4	RTBDN	TRIM25
CD82	ELMOD3	IKBKB	MTA2	SCARB1	TSPY1
CDH10	EME1	INTS7	MYC	SCIN	URB2
CHRD	EXD2	IPP	NAPA	SLC12A3	VEGFC
CHRNE	FAM19A1	KIF21A	NAPG	SLC26A2	VTA1
CHST12	FAM96B	KRR1	NOTCH1	SMARCB1	XAGE-4
CLEC10A	FANCD2	KRT72	NR2E3	SOST	ZCCHC8
CLHC1	FGD3	LRRN4	NYAP2	SPERT	ZNF441
CMBL	FILIP1	MAL	PARP15	SRRD	ZNF506
COL6A4P1	FSCB	MAPKAPK2	PITX2	SRSF11	
CRNN	GLE1	MBD3L4	PKD2	STAP1	

Table S1: 100 random human genes for gene-centric curation. We randomly selected these genes from the set of genes that linked to the publications retrieved from the PubMed query “alternative splicing”. We also gene-centrically curated the mouse orthologues of these genes.

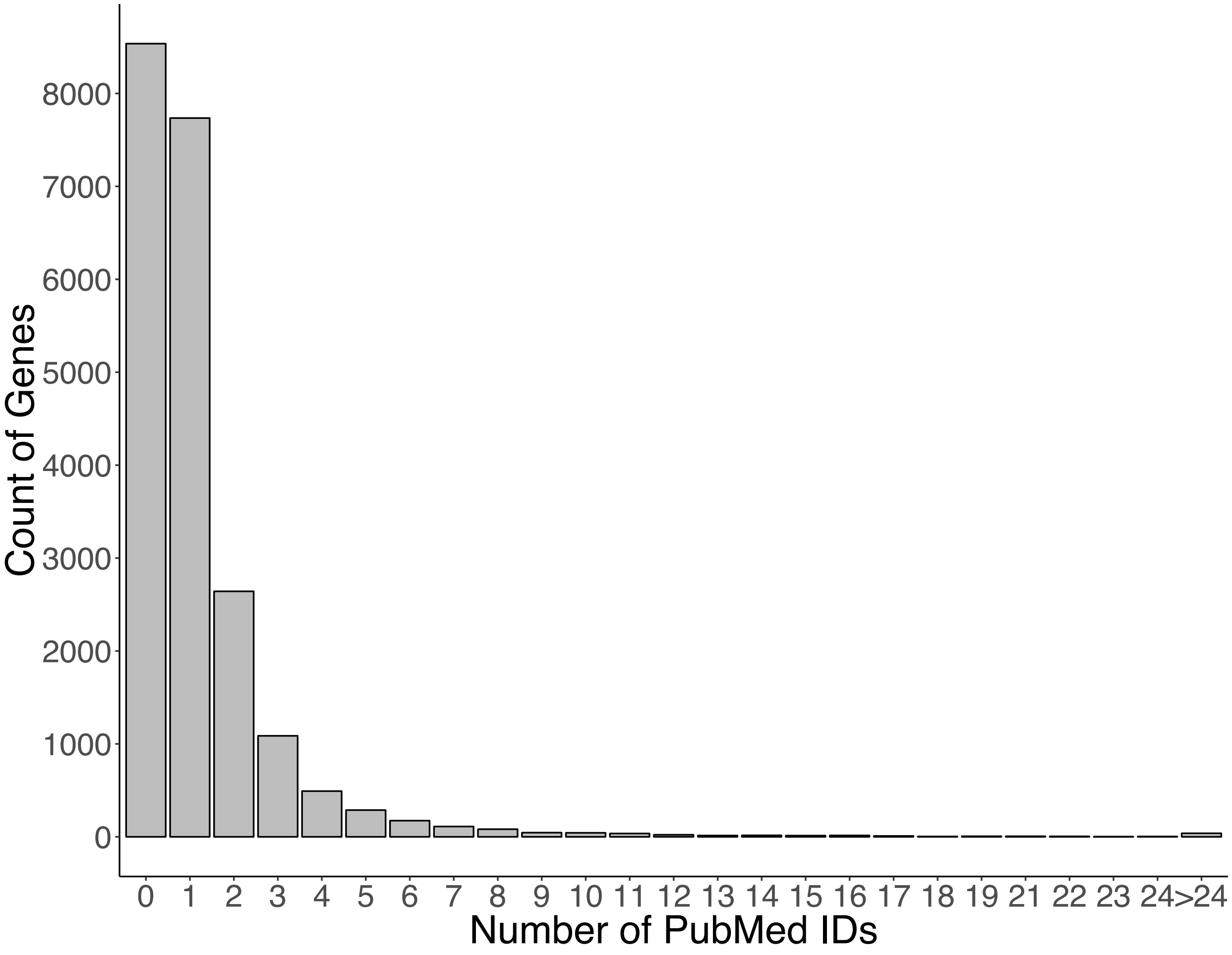

Figure S1: Most human genes have one study linked to alternative splicing in PubMed. The total number of human studies retrieved with the term "alternative splicing" was 19,049. These studies linked to 12,891 human genes. Genes (taken from Ensembl) that were not retrieved from this query were labelled as 0.

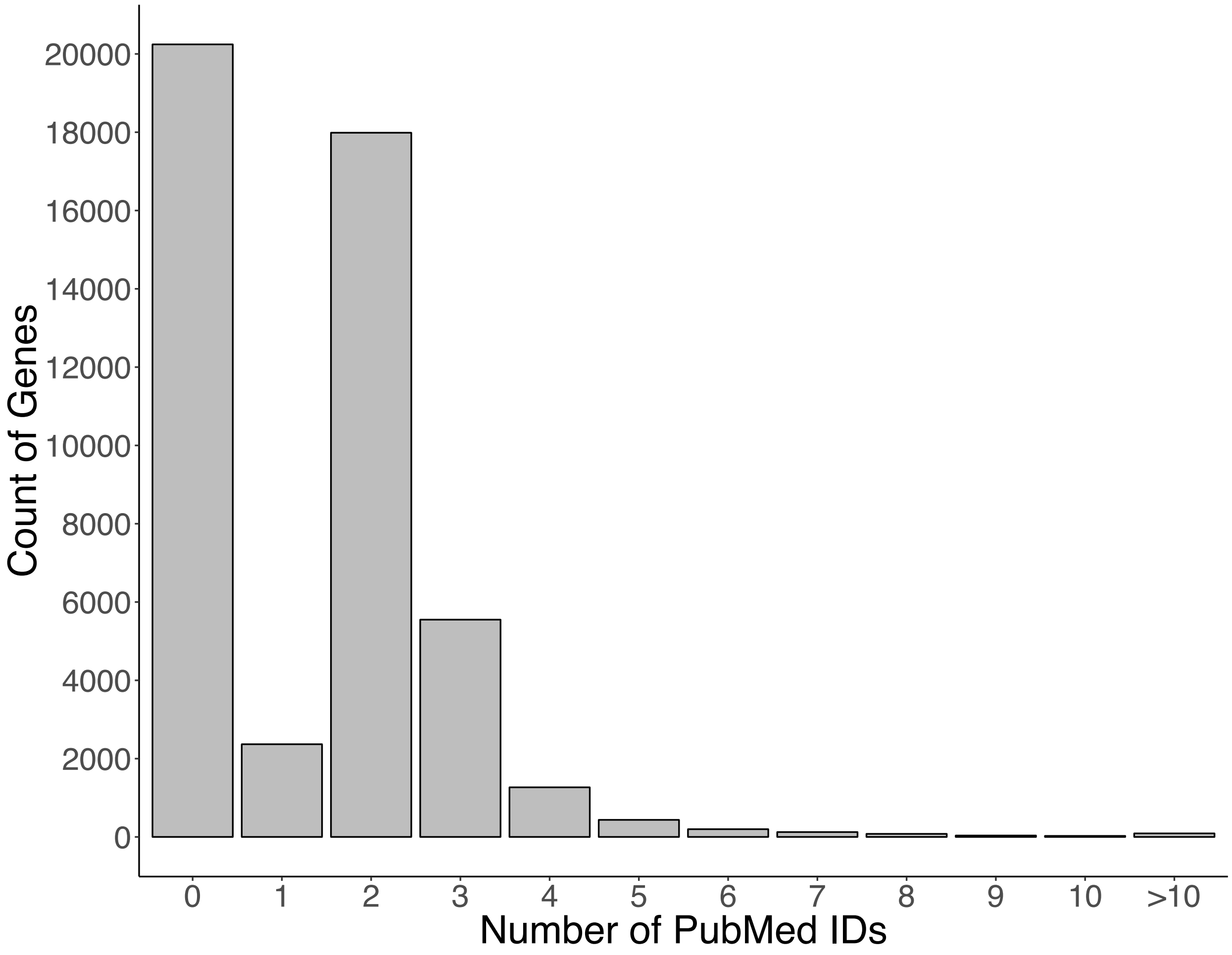

Figure S2: Most mouse genes have two study linked to alternative splicing in PubMed. The total number of mouse studies retrieved with the term "alternative splicing" was 8,203. These studies linked to 28,167 mouse genes. Note that this gene count, unlike the human gene count, include non-protein coding genes. This was likely due to six transcriptome-wide mouse studies which include the term "alternative splicing" and all mouse genes. Removal of these six studies from the query results led to only 7,585 mouse genes associated with a total of 8,197 "alternative splicing"-mentioning studies as described in the Results. Furthermore, after filtering these six studies, most mouse genes had one study which mentioned alternative splicing. We did not see a similar issue in our human query shown in Figure S1. Genes (taken from Ensembl) that were not retrieved from this query were labelled as 0.

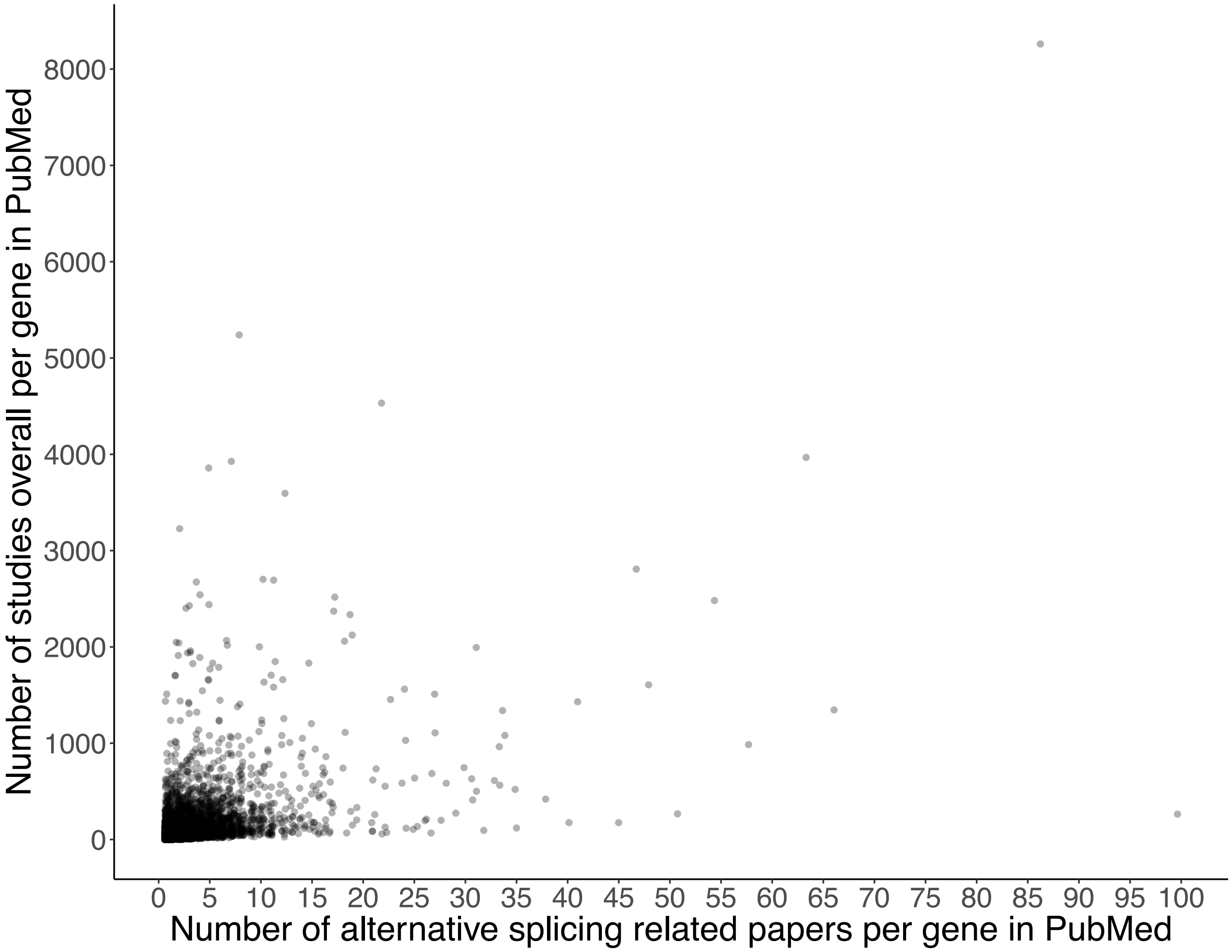

Figure S3: Human genes commonly studied in the context of alternative splicing are commonly well-studied in general. Based on our query of PubMed, we compared the number of papers which mentioned “alternative splicing” to the overall number of studies for a given gene (Spearman’s rank correlation = 0.55).

Section 5: References for all curated studies

1. Abramowski P, Ogrodowczyk C, Martin R, Pongs O. A truncation variant of the cation channel P2RX5 is upregulated during T cell activation. PLoS ONE. 2014;9:e104692.

2. Abrams MT, Robertson NM, Yoon K, Wickstrom E. Inhibition of glucocorticoid-induced apoptosis by targeting the major splice variants of BIM mRNA with small interfering RNA and short hairpin RNA. J Biol Chem. 2004;279:55809–17.

3. Abu-Elheiga L, Matzuk MM, Abo-Hashema KAH, Wakil SJ. Continuous Fatty Acid Oxidation and Reduced Fat Storage in Mice Lacking Acetyl-CoA Carboxylase 2. Science. 2001;291:2613–6.

4. Adams PJ, Garcia E, David LS, Mulatz KJ, Spacey SD, Snutch TP. Ca(V)2.1 P/Q-type calcium channel alternative splicing affects the functional impact of familial hemiplegic migraine mutations: implications for calcium channelopathies. Channels (Austin). 2009;3:110–21.

5. Adato A, Lefèvre G, Delprat B, Michel V, Michalski N, Chardenoux S, et al. Usherin, the defective protein in Usher syndrome type IIA, is likely to be a component of interstereocilia ankle links in the inner ear sensory cells. Hum Mol Genet. 2005;14:3921–32.

6. Adebiyi A, Zhao G, Narayanan D, Thomas-Gatewood CM, Bannister JP, Jaggar JH. Isoform-Selective Physical Coupling of TRPC3 Channels to IP_3 Receptors in Smooth Muscle Cells Regulates Arterial Contractility. Circulation Research. 2010;106:1603–12.

7. Adema GJ, Baas PD. A novel calcitonin-encoding mRNA is produced by alternative processing of calcitonin/calcitonin gene-related peptide-I pre-mRNA. J Biol Chem. 1992;267:7943–8.

8. Aghababaei M, Perdu S, Irvine K, Beristain AG. A disintegrin and metalloproteinase 12 (ADAM12) localizes to invasive trophoblast, promotes cell invasion and directs column outgrowth in early placental development. Mol Hum Reprod. 2014;20:235–49.

9. Ahn AH, Kunkel LM. Syntrophin binds to an alternatively spliced exon of dystrophin. J Cell Biol. 1995;128:363–71.

10. Ahn J, Febbraio M, Silverstein RL. A novel isoform of human Golgi complex-localized glycoprotein-1 (also known as E-selectin ligand-1, MG-160 and cysteine-rich fibroblast growth factor receptor) targets differential subcellular localization. J Cell Sci. 2005;118 Pt 8:1725–31.

11. Aigner A, Juhl H, Malerczyk C, Tkybusch A, Benz CC, Czubayko F. Expression of a truncated 100 kDa HER2 splice variant acts as an endogenous inhibitor of tumour cell proliferation. Oncogene. 2001;20:2101–11.

12. Ali MM, Yoshizawa T, Ishibashi O, Matsuda A, Ikegame M, Shimomura J, et al. PIASxbeta is a key regulator of osterix transcriptional activity and matrix mineralization in osteoblasts. J Cell Sci. 2007;120 Pt 15:2565–73.

13. Alvarado-Kristensson M, Rodríguez MJ, Silió V, Valpuesta JM, Carrera AC. SADB phosphorylation of gamma-tubulin regulates centrosome duplication. Nat Cell Biol. 2009;11:1081–92.

14. Amin EM, Oltean S, Hua J, Gammons MVR, Hamdollah-Zadeh M, Welsh GI, et al. WT1 mutants reveal SRPK1 to be a downstream angiogenesis target by altering VEGF splicing. Cancer Cell. 2011;20:768–80.

15. Amiri S, Movahedin M, Mowla SJ, Hajebrahimi Z, Tavallaei M. Differential gene expression and

alternative splicing of survivin following mouse sciatic nerve injury. Spinal Cord. 2009;47:739–44.

16. An JJ, Gharami K, Liao G-Y, Woo NH, Lau AG, Vanevski F, et al. Distinct role of long 3’ UTR BDNF mRNA in spine morphology and synaptic plasticity in hippocampal neurons. Cell. 2008;134:175–87.

17. Anai S, Goodison S, Shiverick K, Hirao Y, Brown BD, Rosser CJ. Knock-down of Bcl-2 by antisense oligodeoxynucleotides induces radiosensitization and inhibition of angiogenesis in human PC-3 prostate tumor xenografts. Mol Cancer Ther. 2007;6:101–11.

18. Anand S, Batista FD, Tkach T, Efremov DG, Burrone OR. Multiple transcripts of the murine immunoglobulin epsilon membrane locus are generated by alternative splicing and differential usage of two polyadenylation sites. Mol Immunol. 1997;34:175–83.

19. Ando S, Sarlis NJ, Krishnan J, Feng X, Refetoff S, Zhang MQ, et al. Aberrant alternative splicing of thyroid hormone receptor in a TSH-secreting pituitary tumor is a mechanism for hormone resistance. Mol Endocrinol. 2001;15:1529–38.

20. Andolfo I, De Falco L, Asci R, Russo R, Colucci S, Gorrese M, et al. Regulation of divalent metal transporter 1 (DMT1) non-IRE isoform by the microRNA Let-7d in erythroid cells. Haematologica. 2010;95:1244–52.

21. Andrade A, Denome S, Jiang Y-Q, Marangoudakis S, Lipscombe D. Opioid inhibition of N-type Ca2+ channels and spinal analgesia couple to alternative splicing. Nat Neurosci. 2010;13:1249–56.

22. Andreadis A, Broderick JA, Kosik KS. Relative exon affinities and suboptimal splice site signals lead to non-equivalence of two cassette exons. Nucleic Acids Res. 1995;23:3585–93.

23. Andreadis A, Brown WM, Kosik KS. Structure and novel exons of the human tau gene. Biochemistry. 1992;31:10626–33.

24. Andreadis A, Nisson PE, Kosik KS, Watkins PC. The exon trapping assay partly discriminates against alternatively spliced exons. Nucleic Acids Res. 1993;21:2217–21.

25. Aoyama M, Asai K, Shishikura T, Kawamoto T, Miyachi T, Yokoi T, et al. Human neuroblastomas with unfavorable biologies express high levels of brain-derived neurotrophic factor mRNA and a variety of its variants. Cancer Lett. 2001;164:51–60.

26. Apostolatos A, Song S, Acosta S, Peart M, Watson JE, Bickford P, et al. Insulin promotes neuronal survival via the alternatively spliced protein kinase CδII isoform. J Biol Chem. 2012;287:9299–310.

27. Apostolatos H, Apostolatos A, Vickers T, Watson JE, Song S, Vale F, et al. Vitamin A metabolite, all-trans-retinoic acid, mediates alternative splicing of protein kinase C deltaVIII (PKCdeltaVIII) isoform via splicing factor SC35. J Biol Chem. 2010;285:25987–95.

28. Aris JP, Basta PV, Holmes WD, Ballas LM, Moomaw C, Rankl NB, et al. Molecular and biochemical characterization of a recombinant human PKC-δ family member. Biochimica et Biophysica Acta (BBA) - Gene Structure and Expression. 1993;1174:171–81.

29. Arsic N, Gadea G, Lagerqvist EL, Busson M, Cahuzac N, Brock C, et al. The p53 isoform Δ133p53β promotes cancer stem cell potential. Stem Cell Reports. 2015;4:531–40.

30. Atkinson PJ, Cho C-H, Hansen MR, Green SH. Activity of all JNK isoforms contributes to neurite growth in spiral ganglion neurons. Hearing Research. 2011;278:77–85.

31. Azuma N, Tadokoro K, Asaka A, Yamada M, Yamaguchi Y, Handa H, et al. The Pax6 isoform bearing an alternative spliced exon promotes the development of the neural retinal structure. Hum Mol Genet. 2005;14:735–45.

32. Bach I, Yaniv M. More potent transcriptional activators or a transdominant inhibitor of the HNF1 homeoprotein family are generated by alternative RNA processing. EMBO J. 1993;12:4229–42.

33. Bae J, Leo CP, Hsu SY, Hsueh AJ. MCL-1S, a splicing variant of the antiapoptotic BCL-2 family member MCL-1, encodes a proapoptotic protein possessing only the BH3 domain. J Biol Chem. 2000;275:25255–61.

34. Bagnato P, Barone V, Giacomello E, Rossi D, Sorrentino V. Binding of an ankyrin-1 isoform to obscurin suggests a molecular link between the sarcoplasmic reticulum and myofibrils in striated muscles. J Cell Biol. 2003;160:245–53.

35. Bähring R, Dannenberg J, Peters HC, Leicher T, Pongs O, Isbrandt D. Conserved Kv4 N-terminal domain critical for effects of Kv channel-interacting protein 2.2 on channel expression and gating. J Biol Chem. 2001;276:23888–94.

36. Ball SG, Baldock C, Kielty CM, Shuttleworth CA. The role of the C1 and C2 a-domains in type VI collagen assembly. J Biol Chem. 2001;276:7422–30.

37. Bannister JP, Thomas-Gatewood CM, Neeb ZP, Adebiyi A, Cheng X, Jaggar JH. Ca(V)1.2 channel N-terminal splice variants modulate functional surface expression in resistance size artery smooth muscle cells. J Biol Chem. 2011;286:15058–66.

38. Bao J, Lu Z, Joseph JJ, Carabenciov D, Dimond CC, Pang L, et al. Characterization of the murine SIRT3 mitochondrial localization sequence and comparison of mitochondrial enrichment and deacetylase activity of long and short SIRT3 isoforms. J Cell Biochem. 2010;110:238–47.

39. Baraniak AP, Chen JR, Garcia-Blanco MA. Fox-2 mediates epithelial cell-specific fibroblast growth factor receptor 2 exon choice. Mol Cell Biol. 2006;26:1209–22.

40. Barash U, Cohen-Kaplan V, Arvatz G, Gingis-Velitski S, Levy-Adam F, Nativ O, et al. A novel human heparanase splice variant, T5, endowed with protumorigenic characteristics. FASEB J. 2010;24:1239–48.

41. Barbier J, Dutertre M, Bittencourt D, Sanchez G, Gratadou L, de la Grange P, et al. Regulation of H-ras splice variant expression by cross talk between the p53 and nonsense-mediated mRNA decay pathways. Mol Cell Biol. 2007;27:7315–33.

42. Baricault L, Ségui B, Guégand L, Olichon A, Valette A, Larminat F, et al. OPA1 cleavage depends on decreased mitochondrial ATP level and bivalent metals. Experimental Cell Research. 2007;313:3800–8.

43. Bartel F, Schulz J, Böhnke A, Blümke K, Kappler M, Bache M, et al. Significance of HDMX-S (or MDM4) mRNA splice variant overexpression and HDMX gene amplification on primary soft tissue sarcoma prognosis. Int J Cancer. 2005;117:469–75.

44. Baumbusch LO, Myhre S, Langerød A, Bergamaschi A, Geisler SB, Lønning PE, et al. Expression of full-length p53 and its isoform Deltap53 in breast carcinomas in relation to mutation status and clinical parameters. Mol Cancer. 2006;5:47.

45. Bechmann LP, Gastaldelli A, Vetter D, Patman GL, Pascoe L, Hannivoort RA, et al. Glucokinase links Krüppel-like factor 6 to the regulation of hepatic insulin sensitivity in nonalcoholic fatty liver

disease. Hepatology. 2012;55:1083–93.

46. Beffert U, Nematollah Farsian F, Masiulis I, Hammer RE, Yoon SO, Giehl KM, et al. ApoE receptor 2 controls neuronal survival in the adult brain. Curr Biol. 2006;16:2446–52.

47. Bellemare J, Rouleau M, Girard H, Harvey M, Guillemette C. Alternatively spliced products of the UGT1A gene interact with the enzymatically active proteins to inhibit glucuronosyltransferase activity in vitro. Drug Metab Dispos. 2010;38:1785–9.

48. Bellemare J, Rouleau M, Harvey M, Guillemette C. Modulation of the human glucuronosyltransferase UGT1A pathway by splice isoform polypeptides is mediated through protein-protein interactions. J Biol Chem. 2010;285:3600–7.

49. Ben-Hur V, Denichenko P, Siegfried Z, Maimon A, Krainer A, Davidson B, et al. S6K1 alternative splicing modulates its oncogenic activity and regulates mTORC1. Cell Rep. 2013;3:103–15.

50. Ben-Tekaya H, Kahn RA, Hauri H-P. ADP ribosylation factors 1 and 4 and group VIA phospholipase A_2 regulate morphology and intraorganellar traffic in the endoplasmic reticulum-Golgi intermediate compartment. Mol Biol Cell. 2010;21:4130–40.

51. Benedict CL, Gilfillan S, Kearney JF. The long isoform of terminal deoxynucleotidyl transferase enters the nucleus and, rather than catalyzing nontemplated nucleotide addition, modulates the catalytic activity of the short isoform. J Exp Med. 2001;193:89–99.

52. Bergamaschi A, Christensen BL, Katzenellenbogen BS. Reversal of endocrine resistance in breast cancer: interrelationships among 14-3-3ζ, FOXM1, and a gene signature associated with mitosis. Breast Cancer Res. 2011;13:R70.

53. Berge EO, Staalesen V, Straume AH, Lillehaug JR, Lønning PE. Chk2 splice variants express a dominant-negative effect on the wild-type Chk2 kinase activity. Biochim Biophys Acta. 2010;1803:386–95.

54. Berger R, Theodor L, Shoham J, Gokkel E, Brok-Simoni F, Avraham KB, et al. The characterization and localization of the mouse thymopoietin/lamina-associated polypeptide 2 gene and its alternatively spliced products. Genome Res. 1996;6:361–70.

55. Bernasconi R, Pertel T, Luban J, Molinari M. A dual task for the Xbp1-responsive OS-9 variants in the mammalian endoplasmic reticulum: inhibiting secretion of misfolded protein conformers and enhancing their disposal. J Biol Chem. 2008;283:16446–54.

56. Bertolesi GE, Walia Da Silva R, Jollimore C a. B, Shi C, Barnes S, Kelly MEM. Ca(v)3.1 splice variant expression during neuronal differentiation of Y-79 retinoblastoma cells. Neuroscience. 2006;141:259–68.

57. Bhaskar K, Yen S-H, Lee G. Disease-related modifications in tau affect the interaction between Fyn and Tau. J Biol Chem. 2005;280:35119–25.

58. Bianchi M, Amendola R, Federico R, Polticelli F, Mariottini P. Two short protein domains are responsible for the nuclear localization of the mouse spermine oxidase mu isoform. FEBS J. 2005;272:3052–9.

59. Bickmore WA, Oghene K, Little MH, Seawright A, van Heyningen V, Hastie ND. Modulation of DNA binding specificity by alternative splicing of the Wilms tumor wt1 gene transcript. Science. 1992;257:235–7.

60. Bies RD, Phelps SF, Cortez MD, Roberts R, Caskey CT, Chamberlain JS. Human and murine dystrophin mRNA transcripts are differentially expressed during skeletal muscle, heart, and brain development. Nucleic Acids Res. 1992;20:1725–31.

61. Billington CK, Le Jeune IR, Young KW, Hall IP. A major functional role for phosphodiesterase 4D5 in human airway smooth muscle cells. Am J Respir Cell Mol Biol. 2008;38:1–7.

62. Biocca S, Filesi I, Mango R, Maggiore L, Baldini F, Vecchione L, et al. The splice variant LOXIN inhibits LOX-1 receptor function through hetero-oligomerization. J Mol Cell Cardiol. 2008;44:561–70.

63. Birikh KR, Sklan EH, Shoham S, Soreq H. Interaction of “readthrough” acetylcholinesterase with RACK1 and PKCbeta II correlates with intensified fear-induced conflict behavior. Proc Natl Acad Sci USA. 2003;100:283–8.

64. Birukov KG, Csortos C, Marzilli L, Dudek S, Ma SF, Bresnick AR, et al. Differential regulation of alternatively spliced endothelial cell myosin light chain kinase isoforms by p60(Src). J Biol Chem. 2001;276:8567–73.

65. Bismuth K, Maric D, Arnheiter H. MITF and cell proliferation: the role of alternative splice forms. Pigment Cell Res. 2005;18:349–59.

66. Bista M, Petrovich M, Fersht AR. MDMX contains an autoinhibitory sequence element. Proc Natl Acad Sci USA. 2013;110:17814–9.

67. Blanco FJ, Bernabeu C. Alternative splicing factor or splicing factor-2 plays a key role in intron retention of the endoglin gene during endothelial senescence. Aging Cell. 2011;10:896–907.

68. Blanco S, Klimcakova L, Vega FM, Lazo PA. The subcellular localization of vaccinia-related kinase-2 (VRK2) isoforms determines their different effect on p53 stability in tumour cell lines. FEBS J. 2006;273:2487–504.

69. Blöcher S, Fink L, Bohle RM, Bergmann M, Steger K. CREM activator and repressor isoform expression in human male germ cells. Int J Androl. 2005;28:215–23.

70. Blum H, Wolf M, Enssle K, Röllinghoff M, Gessner A. Two distinct stimulus-dependent pathways lead to production of soluble murine interleukin-4 receptor. J Immunol. 1996;157:1846–53.

71. Bock G, Gebhart M, Scharinger A, Jangsangthong W, Busquet P, Poggiani C, et al. Functional properties of a newly identified C-terminal splice variant of Cav1.3 L-type Ca2+ channels. J Biol Chem. 2011;286:42736–48.

72. Bode S, Peters C, Deussing JM. Placental cathepsin M is alternatively spliced and exclusively expressed in the spongiotrophoblast layer. Biochim Biophys Acta. 2005;1731:160–7.

73. Boise LH, González-García M, Postema CE, Ding L, Lindsten T, Turka LA, et al. bcl-x, a bcl-2-related gene that functions as a dominant regulator of apoptotic cell death. Cell. 1993;74:597–608.

74. Bombail V, Collins F, Brown P, Saunders PTK. Modulation of ER alpha transcriptional activity by the orphan nuclear receptor ERR beta and evidence for differential effects of long- and short-form splice variants. Mol Cell Endocrinol. 2010;314:53–61.

75. Bongers G, Krueger KM, Miller TR, Baranowski JL, Estvander BR, Witte DG, et al. An 80-amino acid deletion in the third intracellular loop of a naturally occurring human histamine H3 isoform confers pharmacological differences and constitutive activity. J Pharmacol Exp Ther. 2007;323:888–98.

76. Bonora E, Bianco F, Cordeddu L, Bamshad M, Francescatto L, Dowless D, et al. Mutations in RAD21 disrupt regulation of APOB in patients with chronic intestinal pseudo-obstruction. Gastroenterology. 2015;148:771-782.e11.

77. Boone M, Mobasheri A, Fenton RA, van Balkom BWM, Wismans R, van der Zee CEEM, et al. The lysosomal trafficking regulator interacting protein-5 localizes mainly in epithelial cells. J Mol Histol. 2010;41:61–74.

78. Borner C, Martinou I, Mattmann C, Irmler M, Schaerer E, Martinou JC, et al. The protein bcl-2 alpha does not require membrane attachment, but two conserved domains to suppress apoptosis. J Cell Biol. 1994;126:1059–68.

79. Bouffard F, Plourde K, Bélanger S, Ouellette G, Labrie Y, Durocher F. Analysis of a FANCE splice isoform in regard to DNA repair. J Mol Biol. 2015;427:3056–73.

80. Bouillet P, Zhang LC, Huang DC, Webb GC, Bottema CD, Shore P, et al. Gene structure alternative splicing, and chromosomal localization of pro-apoptotic Bcl-2 relative Bim. Mamm Genome. 2001;12:163–8.

81. Bourdon J-C, Fernandes K, Murray-Zmijewski F, Liu G, Diot A, Xirodimas DP, et al. p53 isoforms can regulate p53 transcriptional activity. Genes Dev. 2005;19:2122–37.

82. Boyle LH, Traherne JA, Plotnek G, Ward R, Trowsdale J. Splice variation in the cytoplasmic domains of myelin oligodendrocyte glycoprotein affects its cellular localisation and transport. J Neurochem. 2007;102:1853–62.

83. Bozzi F, Conca E, Laurini E, Posocco P, Lo Sardo A, Jocollè G, et al. In vitro and in silico studies of MDM2/MDMX isoforms predict Nutlin-3A sensitivity in well/de-differentiated liposarcomas. Lab Invest. 2013;93:1232–40.

84. Brandes C, Kahr L, Stockinger W, Hiesberger T, Schneider WJ, Nimpf J. Alternative splicing in the ligand binding domain of mouse ApoE receptor-2 produces receptor variants binding reelin but not alpha 2-macroglobulin. J Biol Chem. 2001;276:22160–9.

85. Brandt P, Neve RL, Kammesheidt A, Rhoads RE, Vanaman TC. Analysis of the tissue-specific distribution of mRNAs encoding the plasma membrane calcium-pumping ATPases and characterization of an alternately spliced form of PMCA4 at the cDNA and genomic levels. J Biol Chem. 1992;267:4376–85.

86. Brandt P, Ibrahim E, Bruns GAP, Neve RL. Determination of the nucleotide sequence and chromosomal localization of the ATP2B2 Gene Encoding Human Ca2+-Pumping ATPase Isoform PMCA2. Genomics. 1992;14:484–7.

87. Bratt A, Birot O, Sinha I, Veitonmäki N, Aase K, Ernkvist M, et al. Angiomotin regulates endothelial cell-cell junctions and cell motility. J Biol Chem. 2005;280:34859–69.

88. Brenman JE, Xia H, Chao DS, Black SM, Bredt DS. Regulation of neuronal nitric oxide synthase through alternative transcripts. Dev Neurosci. 1997;19:224–31.

89. Brignatz C, Paronetto MP, Opi S, Cappellari M, Audebert S, Feuillet V, et al. Alternative splicing modulates autoinhibition and SH3 accessibility in the Src kinase Fyn. Mol Cell Biol. 2009;29:6438–48.

90. Brockschmidt FF, Heilmann S, Ellis JA, Eigelshoven S, Hanneken S, Herold C, et al. Susceptibility variants on chromosome 7p21.1 suggest HDAC9 as a new candidate gene for male-pattern

baldness. Br J Dermatol. 2011;165:1293–302.

91. Brodbeck D, Cron P, Hemmings BA. A human protein kinase Bgamma with regulatory phosphorylation sites in the activation loop and in the C-terminal hydrophobic domain. J Biol Chem. 1999;274:9133–6.

92. Brodbeck D, Hill MM, Hemmings BA. Two splice variants of protein kinase B gamma have different regulatory capacity depending on the presence or absence of the regulatory phosphorylation site serine 472 in the carboxyl-terminal hydrophobic domain. J Biol Chem. 2001;276:29550–8.

93. Brunkow ME, Gardner JC, Van Ness J, Paeper BW, Kovacevich BR, Proll S, et al. Bone dysplasia sclerosteosis results from loss of the SOST gene product, a novel cystine knot-containing protein. Am J Hum Genet. 2001;68:577–89.

94. Bruno AM, Huang JY, Bennett DA, Marr RA, Hastings ML, Stutzmann GE. Altered ryanodine receptor expression in mild cognitive impairment and Alzheimer's disease. Neurobiol Aging. 2012;33:1001.e1-6.

95. Bunker JM, Wilson L, Jordan MA, Feinstein SC. Modulation of microtubule dynamics by tau in living cells: implications for development and neurodegeneration. Mol Biol Cell. 2004;15:2720–8.

96. Buratti E, Dörk T, Zuccato E, Pagani F, Romano M, Baralle FE. Nuclear factor TDP-43 and SR proteins promote in vitro and in vivo CFTR exon 9 skipping. EMBO J. 2001;20:1774–84.

97. Burgess RW, Nguyen QT, Son YJ, Lichtman JW, Sanes JR. Alternatively spliced isoforms of nerve- and muscle-derived agrin: their roles at the neuromuscular junction. Neuron. 1999;23:33–44.

98. Buss F, Arden SD, Lindsay M, Luzio JP, Kendrick-Jones J. Myosin VI isoform localized to clathrin-coated vesicles with a role in clathrin-mediated endocytosis. EMBO J. 2001;20:3676–84.

99. Byun H-J, Hong I-K, Kim E, Jin Y-J, Jeoung D-I, Hahn J-H, et al. A splice variant of CD99 increases motility and MMP-9 expression of human breast cancer cells through the AKT-, ERK-, and JNK-dependent AP-1 activation signaling pathways. J Biol Chem. 2006;281:34833–47.

100. Cabral RM, Wan H, Cole CL, Abrams DJ, Kelsell DP, South AP. Identification and characterization of DSPIa, a novel isoform of human desmoplakin. Cell Tissue Res. 2010;341:121–9.

101. Caldas H, Fangusaro JR, Boué DR, Holloway MP, Altura RA. Dissecting the role of endothelial SURVIVIN DeltaEx3 in angiogenesis. Blood. 2007;109:1479–89.

102. Caldas H, Jiang Y, Holloway MP, Fangusaro J, Mahotka C, Conway EM, et al. Survivin splice variants regulate the balance between proliferation and cell death. Oncogene. 2005;24:1994–2007.

103. Calvo D, Vega MA. Identification, primary structure, and distribution of CLA-1, a novel member of the CD36/LIMPII gene family. J Biol Chem. 1993;268:18929–35.

104. Candi E, Rufini A, Terrinoni A, Dinsdale D, Ranalli M, Paradisi A, et al. Differential roles of p63 isoforms in epidermal development: selective genetic complementation in p63 null mice. Cell Death Differ. 2006;13:1037–47.

105. Cánovas J, Berndt FA, Sepúlveda H, Aguilar R, Veloso FA, Montecino M, et al. The Specification of Cortical Subcerebral Projection Neurons Depends on the Direct Repression of TBR1 by CTIP1/BCL11a. J Neurosci. 2015;35:7552–64.

106. Cao W, Garcia-Blanco MA. A serine/arginine-rich domain in the human U1 70k protein is necessary and sufficient for ASF/SF2 binding. J Biol Chem. 1998;273:20629–35.

107. Cardona C, Sánchez-Mejías E, Dávila JC, Martín-Rufián M, Campos-Sandoval JA, Vitorica J, et al. Expression of Gls and Gls2 glutaminase isoforms in astrocytes. Glia. 2015;63:365–82.

108. Caride AJ, Filoteo AG, Enyedi A, Verma AK, Penniston JT. Detection of isoform 4 of the plasma membrane calcium pump in human tissues by using isoform-specific monoclonal antibodies. Biochemical Journal. 1996;316:353–9.

109. Casas F, Busson M, Grandemange S, Seyer P, Carazo A, Pessemesse L, et al. Characterization of a novel thyroid hormone receptor alpha variant involved in the regulation of myoblast differentiation. Mol Endocrinol. 2006;20:749–63.

110. Cascino I, Fiucci G, Papoff G, Ruberti G. Three functional soluble forms of the human apoptosis-inducing Fas molecule are produced by alternative splicing. J Immunol. 1995;154:2706–13.

111. Castiglioni AJ, Raingo J, Lipscombe D. Alternative splicing in the C-terminus of CaV2.2 controls expression and gating of N-type calcium channels. J Physiol. 2006;576 Pt 1:119–34.

112. Castro MAA, Oliveira MI, Nunes RJ, Fabre S, Barbosa R, Peixoto A, et al. Extracellular isoforms of CD6 generated by alternative splicing regulate targeting of CD6 to the immunological synapse. J Immunol. 2007;178:4351–61.

113. Cattaneo M, Lotti LV, Martino S, Cardano M, Orlandi R, Mariani-Costantini R, et al. Functional characterization of two secreted SEL1L isoforms capable of exporting unassembled substrate. J Biol Chem. 2009;284:11405–15.

114. Cauffman G, Liebaers I, Van Steirteghem A, Van de Velde H. POU5F1 isoforms show different expression patterns in human embryonic stem cells and preimplantation embryos. Stem Cells. 2006;24:2685–91.

115. Cervelli M, Bellini A, Bianchi M, Marcocci L, Nocera S, Polticelli F, et al. Mouse spermine oxidase gene splice variants. Nuclear subcellular localization of a novel active isoform. Eur J Biochem. 2004;271:760–70.

116. Chai Y, Chipitsyna G, Cui J, Liao B, Liu S, Aysola K, et al. c-Fos oncogene regulator Elk-1 interacts with BRCA1 splice variants BRCA1a/1b and enhances BRCA1a/1b-mediated growth suppression in breast cancer cells. Oncogene. 2001;20:1357–67.

117. Chaidarun SS, Swearingen B, Alexander JM. Differential expression of estrogen receptor-beta (ER beta) in human pituitary tumors: functional interactions with ER alpha and a tumor-specific splice variant. J Clin Endocrinol Metab. 1998;83:3308–15.

118. Chan SC, Li Y, Dehm SM. Androgen receptor splice variants activate androgen receptor target genes and support aberrant prostate cancer cell growth independent of canonical androgen receptor nuclear localization signal. J Biol Chem. 2012;287:19736–49.

119. Chan WY, Soloviev MM, Ciruela F, McIlhinney RA. Molecular determinants of metabotropic glutamate receptor 1B trafficking. Mol Cell Neurosci. 2001;17:577–88.

120. Chandrasekharan NV, Dai H, Roos KLT, Evanson NK, Tomsik J, Elton TS, et al. COX-3, a cyclooxygenase-1 variant inhibited by acetaminophen and other analgesic/antipyretic drugs: cloning, structure, and expression. Proc Natl Acad Sci USA. 2002;99:13926–31.

121. Chang AC, Reddel RR. Identification of a second stanniocalcin cDNA in mouse and human: stanniocalcin 2. Mol Cell Endocrinol. 1998;141:95–9.

122. Chang BS, Kelekar A, Harris MH, Harlan JE, Fesik SW, Thompson CB. The BH3 Domain of Bcl-xS Is Required for Inhibition of the Antiapoptotic Function of Bcl-xL. Mol Cell Biol. 1999;19:6673–81.

123. Chang SY, Yong TF, Yu CY, Liang MC, Pletnikova O, Troncoso J, et al. Age and gender-dependent alternative splicing of P/Q-type calcium channel EF-hand. Neuroscience. 2007;145:1026–36.

124. Chao C, Ives KL, Goluszko E, Kolokoltsov AA, Davey RA, Townsend CM, et al. SRC regulates constitutive internalization and rapid resensitization of a cholecystokinin 2 receptor splice variant. J Biol Chem. 2005;280:33368–73.

125. Charton K, Suel L, Henriques SF, Moussu J-P, Bovolenta M, Taillepierre M, et al. Exploiting the CRISPR/Cas9 system to study alternative splicing in vivo: application to titin. Hum Mol Genet. 2016;25:4518–32.

126. Chemin J, Monteil A, Bourinet E, Nargeot J, Lory P. Alternatively spliced alpha(1G) (Ca(V)3.1) intracellular loops promote specific T-type Ca(2+) channel gating properties. Biophys J. 2001;80:1238–50.

127. Chen AM, Perrin MH, Digruccio MR, Vaughan JM, Brar BK, Arias CM, et al. A soluble mouse brain splice variant of type 2alpha corticotropin-releasing factor (CRF) receptor binds ligands and modulates their activity. Proc Natl Acad Sci USA. 2005;102:2620–5.

128. Chen F, Figueroa DJ, Marmorstein AD, Zhang Q, Petrukhin K, Caskey CT, et al. Retina-specific nuclear receptor: A potential regulator of cellular retinaldehyde-binding protein expressed in retinal pigment epithelium and Müller glial cells. Proc Natl Acad Sci USA. 1999;96:15149–54.

129. Chen H-H, Chang J-G, Lu R-M, Peng T-Y, Tarn W-Y. The RNA binding protein hnRNP Q modulates the utilization of exon 7 in the survival motor neuron 2 (SMN2) gene. Mol Cell Biol. 2008;28:6929–38.

130. Chen J-Z, Ji C-N, Gu S-H, Li J-X, Zhao E-P, Huang Y, et al. Over-expression of Bim alpha3, a novel isoform of human Bim, result in cell apoptosis. Int J Biochem Cell Biol. 2004;36:1554–61.

131. Chen J, Crutchley J, Zhang D, Owzar K, Kastan MB. Identification of a DNA Damage-Induced Alternative Splicing Pathway That Regulates p53 and Cellular Senescence Markers. Cancer Discov. 2017;7:766–81.

132. Chen L, Jeffries O, Rowe ICM, Liang Z, Knaus H-G, Ruth P, et al. Membrane trafficking of large conductance calcium-activated potassium channels is regulated by alternative splicing of a transplantable, acidic trafficking motif in the RCK1-RCK2 linker. J Biol Chem. 2010;285:23265–75.

133. Chen M-J, Ma S-M, Dumitrache LC, Hasty P. Biochemical and cellular characteristics of the 3’ -> 5’ exonuclease TREX2. Nucleic Acids Res. 2007;35:2682–94.

134. Chen M, Beaven S, Tontonoz P. Identification and characterization of two alternatively spliced transcript variants of human liver X receptor alpha. J Lipid Res. 2005;46:2570–9.

135. Chen N, Koopmans F, Gordon A, Paliukhovich I, Klaassen RV, van der Schors RC, et al. Interaction proteomics of canonical Caspr2 (CNTNAP2) reveals the presence of two Caspr2 isoforms with overlapping interactomes. Biochimica et Biophysica Acta (BBA) - Proteins and Proteomics. 2015;1854:827–33.

136. Chen S, Townsend K, Goldberg TE, Davies P, Conejero-Goldberg C. MAPT Isoforms: Differential Transcriptional Profiles Related to 3R and 4R Splice Variants. Journal of Alzheimer's Disease. 2010;22:1313–29.

137. Chen Y, Decker KF, Zheng D, Matkovich SJ, Jia L, Dorn GW. A nucleus-targeted alternately spliced Nix/Bnip3L protein isoform modifies nuclear factor κB (NFκB)-mediated cardiac transcription. J Biol Chem. 2013;288:15455–65.

138. Chen Z, Gore BB, Long H, Ma L, Tessier-Lavigne M. Alternative splicing of the Robo3 axon guidance receptor governs the midline switch from attraction to repulsion. Neuron. 2008;58:325–32.

139. Cheng S, Wang L, Deng C-H, Du S-C, Han Z-G. ARID1A represses hepatocellular carcinoma cell proliferation and migration through lncRNA MVIH. Biochemical and Biophysical Research Communications. doi:10.1016/j.bbrc.2017.07.072.

140. Cheng T-L, Liao C-C, Tsai W-H, Lin C-C, Yeh C-W, Teng C-F, et al. Identification and characterization of the mitochondrial targeting sequence and mechanism in human citrate synthase. J Cell Biochem. 2009;107:1002–15.

141. Cheng Y, Wang Y, Li Y, Deng Y, Hu J, Mo X, et al. A novel human gene ZNF415 with five isoforms inhibits AP-1- and p53-mediated transcriptional activity. Biochem Biophys Res Commun. 2006;351:33–9.

142. Chester AH, Azam R, Felkin LE, George R, Brand N. Correlation between vascular responsivensss and expression of novel transcripts of the ETA-receptor in human vascular tissue. Vascul Pharmacol. 2007;46:181–7.

143. Cheung HC, Hai T, Zhu W, Baggerly KA, Tsavachidis S, Krahe R, et al. Splicing factors PTBP1 and PTBP2 promote proliferation and migration of glioma cell lines. Brain. 2009;132:2277–88.

144. Chiaruttini C, Vicario A, Li Z, Baj G, Braiuca P, Wu Y, et al. Dendritic trafficking of BDNF mRNA is mediated by translin and blocked by the G196A (Val66Met) mutation. PNAS. 2009;106:16481–6.

145. Chiba-Falek O, Nichols M, Suchindran S, Guyton J, Ginsburg GS, Barrett-Connor E, et al. Impact of gene variants on sex-specific regulation of human Scavenger receptor class B type 1 (SR-BI) expression in liver and association with lipid levels in a population-based study. BMC Med Genet. 2010;11:9.

146. Chkheidze AN, Liebhaber SA. A novel set of nuclear localization signals determine distributions of the alphaCP RNA-binding proteins. Mol Cell Biol. 2003;23:8405–15.

147. Choi HS, Kim CS, Hwang CK, Song KY, Wang W, Qiu Y, et al. The opioid ligand binding of human mu-opioid receptor is modulated by novel splice variants of the receptor. Biochem Biophys Res Commun. 2006;343:1132–40.

148. Choudhury R, Noakes CJ, McKenzie E, Kox C, Lowe M. Differential clathrin binding and subcellular localization of OCRL1 splice isoforms. J Biol Chem. 2009;284:9965–73.

149. Chow VT, Quek HH, Tock EP. Alternative splicing of the p53 tumor suppressor gene in the Molt-4 T-lymphoblastic leukemia cell line. Cancer Lett. 1993;73:141–8.

150. Chowdhury B, Tsokos CG, Krishnan S, Robertson J, Fisher CU, Warke RG, et al. Decreased stability and translation of T cell receptor zeta mRNA with an alternatively spliced 3'-untranslated region contribute to zeta chain down-regulation in patients with systemic lupus erythematosus. J

Biol Chem. 2005;280:18959–66.

151. Christmas P, Jones JP, Patten CJ, Rock DA, Zheng Y, Cheng SM, et al. Alternative splicing determines the function of CYP4F3 by switching substrate specificity. J Biol Chem. 2001;276:38166–72.

152. Christofk HR, Vander Heiden MG, Harris MH, Ramanathan A, Gerszten RE, Wei R, et al. The M2 splice isoform of pyruvate kinase is important for cancer metabolism and tumour growth. Nature. 2008;452:230–3.

153. Chu H-Y, Ohtoshi A. Cloning and functional analysis of hypothalamic homeobox gene Bsx1a and its isoform, Bsx1b. Mol Cell Biol. 2007;27:3743–9.

154. Chuvpilo S, Avots A, Berberich-Siebelt F, Glöckner J, Fischer C, Kerstan A, et al. Multiple NF-ATc Isoforms with Individual Transcriptional Properties Are Synthesized in T Lymphocytes. The Journal of Immunology. 1999;162:7294–301.

155. Cianetti L, Segnalini P, Calzolari A, Morsilli O, Felicetti F, Ramoni C, et al. Expression of alternative transcripts of ferroportin-1 during human erythroid differentiation. Haematologica. 2005;90:1595–606.

156. Clark LN, Poorkaj P, Wszolek Z, Geschwind DH, Nasreddine ZS, Miller B, et al. Pathogenic implications of mutations in the tau gene in pallido-ponto-nigral degeneration and related neurodegenerative disorders linked to chromosome 17. Proc Natl Acad Sci USA. 1998;95:13103–7.

157. Clarke E, Rahman N, Page N, Rolph MS, Stewart GJ, Jones GJ. Functional characterization of the atopy-associated gene PHF11. J Allergy Clin Immunol. 2008;121:1148-1154.e3.

158. Clayburgh DR, Rosen S, Witkowski ED, Wang F, Blair S, Dudek S, et al. A differentiation-dependent splice variant of myosin light chain kinase, MLCK1, regulates epithelial tight junction permeability. J Biol Chem. 2004;279:55506–13.

159. Clere N, Bermont L, Fauconnet S, Lascombe I, Saunier M, Vettoretti L, et al. The human papillomavirus type 18 E6 oncoprotein induces Vascular Endothelial Growth Factor 121 (VEGF121) transcription from the promoter through a p53-independent mechanism. Exp Cell Res. 2007;313:3239–50.

160. Clower CV, Chatterjee D, Wang Z, Cantley LC, Heiden MGV, Krainer AR. The alternative splicing repressors hnRNP A1/A2 and PTB influence pyruvate kinase isoform expression and cell metabolism. PNAS. 2010;107:1894–9.

161. Clybouw C, Merino D, Nebl T, Masson F, Robati M, O'Reilly L, et al. Alternative splicing of Bim and Erk-mediated Bim(EL) phosphorylation are dispensable for hematopoietic homeostasis in vivo. Cell Death Differ. 2012;19:1060–8.

162. Cogan J, Austin E, Hedges L, Womack B, West J, Loyd J, et al. Role of BMPR2 alternative splicing in heritable pulmonary arterial hypertension penetrance. Circulation. 2012;126:1907–16.

163. Cohen CD, Doran PP, Blattner SM, Merkle M, Wang GQ, Schmid H, et al. Sam68-like mammalian protein 2, identified by digital differential display as expressed by podocytes, is induced in proteinuria and involved in splice site selection of vascular endothelial growth factor. J Am Soc Nephrol. 2005;16:1958–65.

164. Coldwell MJ, Sack U, Cowan JL, Barrett RM, Vlasak M, Sivakumaran K, et al. Multiple isoforms of

the translation initiation factor eIF4GII are generated via use of alternative promoters, splice sites and a non-canonical initiation codon. Biochem J. 2012;448:1–11.

165. Connell JW, Rodriguez-Martin T, Gibb GM, Kahn NM, Grierson AJ, Hanger DP, et al. Quantitative analysis of tau isoform transcripts in sporadic tauopathies. Brain Res Mol Brain Res. 2005;137:104–9.

166. Conrad C, Zhu J, Conrad C, Schoenfeld D, Fang Z, Ingelsson M, et al. Single molecule profiling of tau gene expression in Alzheimer’s disease. J Neurochem. 2007;103:1228–36.

167. Convery E, Shin EK, Ding Q, Wang W, Douglas P, Davis LS, et al. Inhibition of homologous recombination by variants of the catalytic subunit of the DNA-dependent protein kinase (DNA-PKcs). Proc Natl Acad Sci USA. 2005;102:1345–50.

168. Corominas R, Yang X, Lin GN, Kang S, Shen Y, Ghamsari L, et al. Protein interaction network of alternatively spliced isoforms from brain links genetic risk factors for autism. Nat Commun. 2014;5:3650.

169. Côté J, Dupuis S, Wu JY. Polypyrimidine Track-binding Protein Binding Downstream of Caspase-2 Alternative Exon 9 Represses Its Inclusion. J Biol Chem. 2001;276:8535–43.

170. Craven AJ, Ormandy CJ, Robertson FG, Wilkins RJ, Kelly PA, Nixon AJ, et al. Prolactin Signaling Influences the Timing Mechanism of the Hair Follicle: Analysis of Hair Growth Cycles in Prolactin Receptor Knockout Mice. Endocrinology. 2001;142:2533–9.

171. Crawford PA, Gaddie KJ, Smith TM, Kirley TL. Characterization of an alternative splice variant of human nucleoside triphosphate diphosphohydrolase 3 (NTPDase3): a possible modulator of nucleotidase activity and purinergic signaling. Arch Biochem Biophys. 2007;457:7–15.

172. Cribbs LL, Gomora JC, Daud AN, Lee JH, Perez-Reyes E. Molecular cloning and functional expression of Ca(v)3.1c, a T-type calcium channel from human brain. FEBS Lett. 2000;466:54–8.

173. Cuchet D, Sykes A, Nicolas A, Orr A, Murray J, Sirma H, et al. PML isoforms I and II participate in PML-dependent restriction of HSV-1 replication. J Cell Sci. 2011;124 Pt 2:280–91.

174. Cunha SR, Le Scouarnec S, Schott J-J, Mohler PJ. Exon organization and novel alternative splicing of the human ANK2 gene: implications for cardiac function and human cardiac disease. J Mol Cell Cardiol. 2008;45:724–34.

175. Curnow KM, Pascoe L, Davies E, White PC, Corvol P, Clauser E. Alternatively spliced human type 1 angiotensin II receptor mRNAs are translated at different efficiencies and encode two receptor isoforms. Mol Endocrinol. 1995;9:1250–62.

176. D’Souza I, Schellenberg GD. tau Exon 10 expression involves a bipartite intron 10 regulatory sequence and weak 5’ and 3’ splice sites. J Biol Chem. 2002;277:26587–99.

177. D’Souza VN, Man N thi, Morris GE, Karges W, Pillers D-AM, Ray PN. A novel dystrophin isoform is required for normal retinal electrophysiology. Hum Mol Genet. 1995;4:837–42.

178. da Glória VG, Martins de Araújo M, Mafalda Santos A, Leal R, de Almeida SF, Carmo AM, et al. T cell activation regulates CD6 alternative splicing by transcription dynamics and SRSF1. J Immunol. 2014;193:391–9.

179. Dabertrand F, Morel J-L, Sorrentino V, Mironneau J, Mironneau C, Macrez N. Modulation of calcium signalling by dominant negative splice variant of ryanodine receptor subtype 3 in native smooth

muscle cells. Cell Calcium. 2006;40:11–21.

180. Dai F, Chang C, Lin X, Dai P, Mei L, Feng X-H. Erbin inhibits transforming growth factor beta signaling through a novel Smad-interacting domain. Mol Cell Biol. 2007;27:6183–94.

181. Dale RM, Remo BF, Svensson EC. An alternative transcript of the FOG-2 gene encodes a FOG-2 isoform lacking the FOG repression motif. Biochem Biophys Res Commun. 2007;357:683–7.

182. Dance AL, Miller M, Seragaki S, Aryal P, White B, Aschenbrenner L, et al. Regulation of myosin-VI targeting to endocytic compartments. Traffic. 2004;5:798–813.

183. Datta D, Flaxenburg JA, Laxmanan S, Geehan C, Grimm M, Waaga-Gasser AM, et al. Ras-induced modulation of CXCL10 and its receptor splice variant CXCR3-B in MDA-MB-435 and MCF-7 cells: relevance for the development of human breast cancer. Cancer Res. 2006;66:9509–18.

184. Davies JQ, Chang G-W, Yona S, Gordon S, Stacey M, Lin H-H. The role of receptor oligomerization in modulating the expression and function of leukocyte adhesion-G protein-coupled receptors. J Biol Chem. 2007;282:27343–53.

185. Davis LH, Davis JQ, Bennett V. Ankyrin regulation: an alternatively spliced segment of the regulatory domain functions as an intramolecular modulator. J Biol Chem. 1992;267:18966–72.

186. Day TW, Huang S, Safa AR. c-FLIP knockdown induces ligand-independent DR5-, FADD-, caspase-8-, and caspase-9-dependent apoptosis in breast cancer cells. Biochem Pharmacol. 2008;76:1694–704.

187. De Angelis E, Brummendorf T, Cheng L, Lemmon V, Kenwrick S. Alternative use of a mini exon of the L1 gene affects L1 binding to neural ligands. J Biol Chem. 2001;276:32738–42.

188. de Graaf P, Little NA, Ramos YFM, Meulmeester E, Letteboer SJF, Jochemsen AG. Hdmx protein stability is regulated by the ubiquitin ligase activity of Mdm2. J Biol Chem. 2003;278:38315–24.

189. De Jaegere S, Wuytack F, De Smedt H, Van den Bosch L, Casteels R. Alternative processing of the gene transcripts encoding a plasma-membrane and a sarco/endoplasmic reticulum Ca2+ pump during differentiation of BC3H1 muscle cells. Biochim Biophys Acta. 1993;1173:188–94.

190. de la Luna S, Burden MJ, Lee CW, La Thangue NB. Nuclear accumulation of the E2F heterodimer regulated by subunit composition and alternative splicing of a nuclear localization signal. J Cell Sci. 1996;109 (Pt 10):2443–52.

191. De Laurenzi V, Costanzo A, Barcaroli D, Terrinoni A, Falco M, Annicchiarico-Petruzzelli M, et al. Two new p73 splice variants, gamma and delta, with different transcriptional activity. J Exp Med. 1998;188:1763–8.

192. Decher N, Uyguner O, Scherer CR, Karaman B, Yüksel-Apak M, Busch AE, et al. hKChIP2 is a functional modifier of hKv4.3 potassium channels: cloning and expression of a short hKChIP2 splice variant. Cardiovasc Res. 2001;52:255–64.

193. Dehm SM, Schmidt LJ, Heemers HV, Vessella RL, Tindall DJ. Splicing of a novel androgen receptor exon generates a constitutively active androgen receptor that mediates prostate cancer therapy resistance. Cancer Res. 2008;68:5469–77.

194. Delahaye NF, Rusakiewicz S, Martins I, Ménard C, Roux S, Lyonnet L, et al. Alternatively spliced NKp30 isoforms affect the prognosis of gastrointestinal stromal tumors. Nat Med. 2011;17:700–7.

195. Delisle MB, Murrell JR, Richardson R, Trofatter JA, Rascol O, Soulages X, et al. A mutation at codon 279 (N279K) in exon 10 of the Tau gene causes a tauopathy with dementia and supranuclear palsy. Acta Neuropathol. 1999;98:62–77.

196. Denecke J, Kranz C, von Kleist-Retzow JC, Bosse K, Herkenrath P, Debus O, et al. Congenital disorder of glycosylation type Id: clinical phenotype, molecular analysis, prenatal diagnosis, and glycosylation of fetal proteins. Pediatr Res. 2005;58:248–53.

197. Desai J, Velo MPR, Yamada K, Overman LM, Engle EC. Spatiotemporal expression pattern of KIF21A during normal embryonic development and in congenital fibrosis of the extraocular muscles type 1 (CFEOM1). Gene Expr Patterns. 2012;12:180–8.

198. Di Modugno F, DeMonte L, Balsamo M, Bronzi G, Nicotra MR, Alessio M, et al. Molecular cloning of hMena (ENAH) and its splice variant hMena+11a: epidermal growth factor increases their expression and stimulates hMena+11a phosphorylation in breast cancer cell lines. Cancer Res. 2007;67:2657–65.

199. Diamond SE, Chiono M, Gutierrez-Hartmann A. Reconstitution of the protein kinase A response of the rat prolactin promoter: differential effects of distinct Pit-1 isoforms and functional interaction with Oct-1. Mol Endocrinol. 1999;13:228–38.

200. Dickie LJ, Aziz AM, Savic S, Lucherini OM, Cantarini L, Geiler J, et al. Involvement of X-box binding protein 1 and reactive oxygen species pathways in the pathogenesis of tumour necrosis factor receptor-associated periodic syndrome. Ann Rheum Dis. 2012;71:2035–43.

201. Diez J, Park Y, Zeller M, Brown D, Garza D, Ricordi C, et al. Differential splicing of the IA-2 mRNA in pancreas and lymphoid organs as a permissive genetic mechanism for autoimmunity against the IA-2 type 1 diabetes autoantigen. Diabetes. 2001;50:895–900.

202. Diveu C, Venereau E, Froger J, Ravon E, Grimaud L, Rousseau F, et al. Molecular and functional characterization of a soluble form of oncostatin M/interleukin-31 shared receptor. J Biol Chem. 2006;281:36673–82.

203. Dobbyn HC, Hill K, Hamilton TL, Spriggs KA, Pickering BM, Coldwell MJ, et al. Regulation of BAG-1 IRES-mediated translation following chemotoxic stress. Oncogene. 2007;27:1167–74.

204. Dodt G, Warren D, Becker E, Rehling P, Gould SJ. Domain mapping of human PEX5 reveals functional and structural similarities to Saccharomyces cerevisiae Pex18p and Pex21p. J Biol Chem. 2001;276:41769–81.

205. Dolatshad H, Pellagatti A, Liberante FG, Llorian M, Repapi E, Steeples V, et al. Cryptic splicing events in the iron transporter ABCB7 and other key target genes in SF3B1-mutant myelodysplastic syndromes. Leukemia. 2016;30:2322–31.

206. Dolzhanskaya N, Merz G, Denman RB. Alternative splicing modulates protein arginine methyltransferase-dependent methylation of fragile X syndrome mental retardation protein. Biochemistry. 2006;45:10385–93.

207. Donkor J, Sariahmetoglu M, Dewald J, Brindley DN, Reue K. Three Mammalian Lipins Act as Phosphatidate Phosphatases with Distinct Tissue Expression Patterns. J Biol Chem. 2007;282:3450–7.

208. Du S, Lawrence EJ, Strzelecki D, Rajput P, Xia SJ, Gottesman DM, et al. Co-expression of alternatively spliced forms of PAX3, PAX7, PAX3-FKHR and PAX7-FKHR with distinct DNA binding and transactivation properties in rhabdomyosarcoma. Int J Cancer. 2005;115:85–92.

209. Dubey D, Ganesh S. Modulation of functional properties of laforin phosphatase by alternative splicing reveals a novel mechanism for the EPM2A gene in Lafora progressive myoclonus epilepsy. Hum Mol Genet. 2008;17:3010–20.

210. Dubielecka PM, Cui P, Xiong X, Hossain S, Heck S, Angelov L, et al. Differential regulation of macropinocytosis by Abi1/Hssh3bp1 isoforms. PLoS ONE. 2010;5:e10430.

211. Dudziak D, Nimmerjahn F, Bornkamm GW, Laux G. Alternative splicing generates putative soluble CD83 proteins that inhibit T cell proliferation. J Immunol. 2005;174:6672–6.

212. Dunn NR, Koonce CH, Anderson DC, Islam A, Bikoff EK, Robertson EJ. Mice exclusively expressing the short isoform of Smad2 develop normally and are viable and fertile. Genes Dev. 2005;19:152–63.

213. Duterque-Coquillaud M, Niel C, Plaza S, Stehelin D. New human erg isoforms generated by alternative splicing are transcriptional activators. Oncogene. 1993;8:1865–73.

214. Dutta P, Bui T, Bauckman KA, Keyomarsi K, Mills GB, Nanjundan M. EVI1 splice variants modulate functional responses in ovarian cancer cells. Mol Oncol. 2013;7:647–68.

215. Ealovega MW, McGinnis PK, Sumantran VN, Clarke MF, Wicha MS. bcl-xs gene therapy induces apoptosis of human mammary tumors in nude mice. Cancer Res. 1996;56:1965–9.

216. Eckhardt ERM, Cai L, Sun B, Webb NR, van der Westhuyzen DR. High density lipoprotein uptake by scavenger receptor SR-BII. J Biol Chem. 2004;279:14372–81.

217. Edovitsky E, Elkin M, Zcharia E, Peretz T, Vlodavsky I. Heparanase gene silencing, tumor invasiveness, angiogenesis, and metastasis. J Natl Cancer Inst. 2004;96:1219–30.

218. Endo N, Rutledge SJ, Opas EE, Vogel R, Rodan GA, Schmidt A. Human protein tyrosine phosphatase-σ: Alternative splicing and inhibition by bisphosphonates. J Bone Miner Res. 1996;11:535–43.

219. Erşahin C, Szpaderska AM, Orawski AT, Simmons WH. Aminopeptidase P isozyme expression in human tissues and peripheral blood mononuclear cell fractions. Arch Biochem Biophys. 2005;435:303–10.

220. Ertongur-Fauth T, Hochheimer A, Buescher JM, Rapprich S, Krohn M. A novel TMEM16A splice variant lacking the dimerization domain contributes to calcium-activated chloride secretion in human sweat gland epithelial cells. Exp Dermatol. 2014;23:825–31.

221. Eshel D, Toporik A, Efrati T, Nakav S, Chen A, Douvdevani A. Characterization of natural human antagonistic soluble CD40 isoforms produced through alternative splicing. Mol Immunol. 2008;46:250–7.

222. Essand M, Vikman S, Grawé J, Gedda L, Hellberg C, Oberg K, et al. Identification and characterization of a novel splicing variant of vesicular monoamine transporter 1. J Mol Endocrinol. 2005;35:489–501.

223. Estes PA, Cooke NE, Liebhaber SA. A native RNA secondary structure controls alternative splice-site selection and generates two human growth hormone isoforms. J Biol Chem. 1992;267:14902–8.

224. Esteves SLC, Korrodi-Gregório L, Cotrim CZ, Kleeff PJM van, Domingues SC, Silva OAB da C e, et al. Protein Phosphatase 1γ Isoforms Linked Interactions in the Brain. J Mol Neurosci.

2013;50:179–97.

225. Evans RT, Seasholtz AF. Soluble corticotropin-releasing hormone receptor 2alpha splice variant is efficiently translated but not trafficked for secretion. Endocrinology. 2009;150:4191–202.

226. Even Y, Durieux S, Escande M-L, Lozano JC, Peaucellier G, Weil D, et al. CDC2L5, a Cdk-like kinase with RS domain, interacts with the ASF/SF2-associated protein p32 and affects splicing in vivo. J Cell Biochem. 2006;99:890–904.

227. Evsyukova I, Bradrick SS, Gregory SG, Garcia-Blanco MA. Cleavage and polyadenylation specificity factor 1 (CPSF1) regulates alternative splicing of interleukin 7 receptor (IL7R) exon 6. RNA. 2013;19:103–15.

228. Fan C, Wang X. Mdm2 Splice isoforms regulate the p53/Mdm2/Mdm4 regulatory circuit via RING domain-mediated ubiquitination of p53 and Mdm4. Cell Cycle. 2017;16:660–4.

229. Fang W, Rivard JJ, Mueller DL, Behrens TW. Cloning and molecular characterization of mouse bcl-x in B and T lymphocytes. J Immunol. 1994;153:4388–98.

230. Fang Z, Luna EJ. Supervillin-mediated Suppression of p53 Protein Enhances Cell Survival. J Biol Chem. 2013;288:7918–29.

231. Farmer C, Cox JJ, Fletcher EV, Woods CG, Wood JN, Schorge S. Splice variants of Na(V)1.7 sodium channels have distinct β subunit-dependent biophysical properties. PLoS ONE. 2012;7:e41750.

232. Farris W, Leissring MA, Hemming ML, Chang AY, Selkoe DJ. Alternative splicing of human insulin-degrading enzyme yields a novel isoform with a decreased ability to degrade insulin and amyloid beta-protein. Biochemistry. 2005;44:6513–25.

233. Fatemi SH, Kroll JL, Stary JM. Altered levels of Reelin and its isoforms in schizophrenia and mood disorders. Neuroreport. 2001;12:3209–15.

234. Fei T, Chen Y, Xiao T, Li W, Cato L, Zhang P, et al. Genome-wide CRISPR screen identifies HNRNPL as a prostate cancer dependency regulating RNA splicing. Proc Natl Acad Sci USA. 2017;114:E5207–15.

235. Feng J, Bi C, Clark BS, Mady R, Shah P, Kohtz JD. The Evf-2 noncoding RNA is transcribed from the Dlx-5/6 ultraconserved region and functions as a Dlx-2 transcriptional coactivator. Genes Dev. 2006;20:1470–84.

236. Feng Y-H, Li X, Wang L, Zhou L, Gorodeski GI. A truncated P2X7 receptor variant (P2X7-j) endogenously expressed in cervical cancer cells antagonizes the full-length P2X7 receptor through hetero-oligomerization. J Biol Chem. 2006;281:17228–37.

237. Fernandez K, Serinagaoglu Y, Hammond S, Martin LT, Martin PT. Mice lacking dystrophin or alpha sarcoglycan spontaneously develop embryonal rhabdomyosarcoma with cancer-associated p53 mutations and alternatively spliced or mutant Mdm2 transcripts. Am J Pathol. 2010;176:416–34.

238. Fernández-Real JM, Straczkowski M, Lainez B, Chacón MR, Kowalska I, López-Bermejo A, et al. An alternative spliced variant of circulating soluble tumor necrosis factor-alpha receptor-2 is paradoxically associated with insulin action. Eur J Endocrinol. 2006;154:723–30.

239. Ferrera L, Caputo A, Ubby I, Bussani E, Zegarra-Moran O, Ravazzolo R, et al. Regulation of TMEM16A chloride channel properties by alternative splicing. J Biol Chem. 2009;284:33360–8.

240. Fiesel FC, Weber SS, Supper J, Zell A, Kahle PJ. TDP-43 regulates global translational yield by splicing of exon junction complex component SKAR. Nucleic Acids Res. 2012;40:2668–82.

241. Fisette J-F, Montagna DR, Mihailescu M-R, Wolfe MS. A G-rich element forms a G-quadruplex and regulates BACE1 mRNA alternative splicing. J Neurochem. 2012;121:763–73.

242. Flaman JM, Waridel F, Estreicher A, Vannier A, Limacher JM, Gilbert D, et al. The human tumour suppressor gene p53 is alternatively spliced in normal cells. Oncogene. 1996;12:813–8.

243. Fleischer A, Ayllón V, Dumoutier L, Renauld J-C, Rebollo A. Proapoptotic activity of ITM2B(s), a BH3-only protein induced upon IL-2-deprivation which interacts with Bcl-2. Oncogene. 2002;21:3181–9.

244. Frankton S, Harvey CB, Gleason LM, Fadel A, Williams GR. Multiple messenger ribonucleic acid variants regulate cell-specific expression of human thyroid hormone receptor beta1. Mol Endocrinol. 2004;18:1631–42.

245. Frémin C, Ezan F, Boisselier P, Bessard A, Pagès G, Pouysségur J, et al. ERK2 but not ERK1 plays a key role in hepatocyte replication: An RNAi-mediated ERK2 knockdown approach in wild-type and ERK1 null hepatocytes. Hepatology. 2007;45:1035–45.

246. French AR, Sosnick TR, Rock RS. Investigations of human myosin VI targeting using optogenetically controlled cargo loading. Proc Natl Acad Sci USA. 2017;114:E1607–16.

247. Frias MA, Thoreen CC, Jaffe JD, Schroder W, Sculley T, Carr SA, et al. mSin1 is necessary for Akt/PKB phosphorylation, and its isoforms define three distinct mTORC2s. Curr Biol. 2006;16:1865–70.

248. Frigo DE, Howe MK, Wittmann BM, Brunner AM, Cushman I, Wang Q, et al. CaM kinase kinase β-mediated activation of the growth regulatory kinase AMPK is required for androgen-dependent migration of prostate cancer cells. Cancer Res. 2011;71:528–37.

249. Froesch BA, Takayama S, Reed JC. BAG-1L protein enhances androgen receptor function. J Biol Chem. 1998;273:11660–6.

250. Fukuda E, Hamada S, Hasegawa S, Katori S, Sanbo M, Miyakawa T, et al. Down-regulation of protocadherin-alpha A isoforms in mice changes contextual fear conditioning and spatial working memory. Eur J Neurosci. 2008;28:1362–76.

251. Fukuzawa A, Idowu S, Gautel M. Complete human gene structure of obscurin: implications for isoform generation by differential splicing. J Muscle Res Cell Motil. 2005;26:427–34.

252. Gadea G, Arsic N, Fernandes K, Diot A, Joruiz SM, Abdallah S, et al. TP53 drives invasion through expression of its Δ133p53β variant. eLife. 2016;5:e14734.

253. Gammons MV, Lucas R, Dean R, Coupland SE, Oltean S, Bates DO. Targeting SRPK1 to control VEGF-mediated tumour angiogenesis in metastatic melanoma. Br J Cancer. 2014;111:477–85.

254. Gao Z, Godbout R. Reelin-Disabled-1 signaling in neuronal migration: splicing takes the stage. Cell Mol Life Sci. 2013;70:2319–29.

255. García JF, Villuendas R, Sánchez-Beato M, Sánchez-Aguilera A, Sánchez L, Prieto I, et al. Nucleolar p14(ARF) overexpression in Reed-Sternberg cells in Hodgkin's lymphoma: absence of p14(ARF)/Hdm2 complexes is associated with expression of alternatively spliced Hdm2 transcripts. Am J Pathol. 2002;160:569–78.

256. García-Alai MM, Tidow H, Natan E, Townsley FM, Veprintsev DB, Fersht AR. The novel p53 isoform “delta p53” is a misfolded protein and does not bind the p21 promoter site. Protein Sci. 2008;17:1671–8.

257. Garrison S, Hojgaard A, Patillo D, Weis JJ, Weis JH. Functional characterization of Pactolus, a beta-integrin-like protein preferentially expressed by neutrophils. J Biol Chem. 2001;276:35500–11.

258. Garzon D, Yu G, Fahnestock M. A new brain-derived neurotrophic factor transcript and decrease in brain-derived neurotrophic factor transcripts 1, 2 and 3 in Alzheimer’s disease parietal cortex. J Neurochem. 2002;82:1058–64.

259. Gasdaska PY, Fisher H, Powis G. An alternatively spliced form of NQO1 (DT-diaphorase) messenger RNA lacking the putative quinone substrate binding site is present in human normal and tumor tissues. Cancer Res. 1995;55:2542–7.

260. Gassler N, Roth W, Funke B, Schneider A, Herzog F, Tischendorf JJW, et al. Regulation of enterocyte apoptosis by acyl-CoA synthetase 5 splicing. Gastroenterology. 2007;133:587–98.

261. Gateva G, Kremneva E, Reindl T, Kotila T, Kogan K, Gressin L, et al. Tropomyosin Isoforms Specify Functionally Distinct Actin Filament Populations In Vitro. Current Biology. 2017;27:705–13.

262. Gavin AL, Duong B, Skog P, Aït-Azzouzene D, Greaves DR, Scott ML, et al. deltaBAFF, a splice isoform of BAFF, opposes full-length BAFF activity in vivo in transgenic mouse models. J Immunol. 2005;175:319–28.

263. Gaytan-Cervantes J, Gonzalez-Torres C, Maldonado V, Zampedri C, Ceballos-Cancino G, Melendez-Zajgla J. Protein Sam68 regulates the alternative splicing of survivin DEx3. J Biol Chem. 2017;292:13745–57.

264. George C, Rogers S, Bertrand B, Tunwell R, Thomas NL, Steele D, et al. Alternative splicing of ryanodine receptors modulates cardiomyocyte Ca2+ signaling and susceptibility to apoptosis. Circulation Research. 2007;100:874–83.

265. Georges R, Nemer G, Morin M, Lefebvre C, Nemer M. Distinct expression and function of alternatively spliced Tbx5 isoforms in cell growth and differentiation. Mol Cell Biol. 2008;28:4052–67.

266. Geppert M, Khvotchev M, Krasnoperov V, Goda Y, Missler M, Hammer RE, et al. Neurexin I alpha is a major alpha-latrotoxin receptor that cooperates in alpha-latrotoxin action. J Biol Chem. 1998;273:1705–10.

267. Gerold KD, Zheng P, Rainbow DB, Zernecke A, Wicker LS, Kissler S. The soluble CTLA-4 splice variant protects from type 1 diabetes and potentiates regulatory T-cell function. Diabetes. 2011;60:1955–63.

268. Ghigna C, Giordano S, Shen H, Benvenuto F, Castiglioni F, Comoglio PM, et al. Cell motility is controlled by SF2/ASF through alternative splicing of the Ron protooncogene. Mol Cell. 2005;20:881–90.

269. Ghosh A, Sarkar SN, Rowe TM, Sen GC. A specific isozyme of 2’-5’ oligoadenylate synthetase is a dual function proapoptotic protein of the Bcl-2 family. J Biol Chem. 2001;276:25447–55.

270. Ghosh A, Stewart D, Matlashewski G. Regulation of human p53 activity and cell localization by alternative splicing. Mol Cell Biol. 2004;24:7987–97.

271. Giglio S, Mancini F, Gentiletti F, Sparaco G, Felicioni L, Barassi F, et al. Identification of an aberrantly spliced form of HDMX in human tumors: a new mechanism for HDM2 stabilization. Cancer Res. 2005;65:9687–94.

272. Gill RBS, Day A, Barstow A, Liu H, Zaman G, Dhoot GK. Sulf2 gene is alternatively spliced in mammalian developing and tumour tissues with functional implications. Biochem Biophys Res Commun. 2011;414:468–73.

273. Girouard J, Lafleur M-J, Parent S, Leblanc V, Asselin E. Involvement of Akt isoforms in chemoresistance of endometrial carcinoma cells. Gynecologic Oncology. 2013;128:335–43.

274. Glatz DC, Rujescu D, Tang Y, Berendt FJ, Hartmann AM, Faltraco F, et al. The alternative splicing of tau exon 10 and its regulatory proteins CLK2 and TRA2-BETA1 changes in sporadic Alzheimer’s disease. J Neurochem. 2006;96:635–44.

275. Glover M, Ware JS, Henry A, Wolley M, Walsh R, Wain LV, et al. Detection of mutations in KLHL3 and CUL3 in families with FHHt (familial hyperkalaemic hypertension or Gordon’s syndrome). Clin Sci. 2014;126:721–6.

276. Gnidehou S, Lacroix L, Sezan A, Ohayon R, Noël-Hudson M-S, Morand S, et al. Cloning and characterization of a novel isoform of iodotyrosine dehalogenase 1 (DEHAL1) DEHAL1C from human thyroid: comparisons with DEHAL1 and DEHAL1B. Thyroid. 2006;16:715–24.

277. Goina E, Skoko N, Pagani F. Binding of DAZAP1 and hnRNPA1/A2 to an exonic splicing silencer in a natural BRCA1 exon 18 mutant. Mol Cell Biol. 2008;28:3850–60.

278. Gonçalves V, Matos P, Jordan P. Antagonistic SR proteins regulate alternative splicing of tumor-related Rac1b downstream of the PI3-kinase and Wnt pathways. Hum Mol Genet. 2009;18:3696–707.

279. Gonçalves V, Theisen P, Antunes O, Medeira A, Ramos JS, Jordan P, et al. A missense mutation in the APC tumor suppressor gene disrupts an ASF/SF2 splicing enhancer motif and causes pathogenic skipping of exon 14. Mutat Res. 2009;662:33–6.

280. Goodson ML, Jonas BA, Privalsky ML. Alternative mRNA splicing of SMRT creates functional diversity by generating corepressor isoforms with different affinities for different nuclear receptors. J Biol Chem. 2005;280:7493–503.

281. Gopalakrishnan S, Van Emburgh BO, Shan J, Su Z, Fields CR, Vieweg J, et al. A novel DNMT3B splice variant expressed in tumor and pluripotent cells modulates genomic DNA methylation patterns and displays altered DNA binding. Mol Cancer Res. 2009;7:1622–34.

282. Górska A, Błaszczyk L, Dutkiewicz M, Ciesiołka J. Length variants of the 5’ untranslated region of p53 mRNA and their impact on the efficiency of translation initiation of p53 and its N-truncated isoform ΔNp53. RNA Biol. 2013;10:1726–40.

283. Gostyńska KB, Lemmink H, Bremer J, Pas HH, Nijenhuis M, van den Akker PC, et al. A PLEC Isoform Identified in Skin, Muscle, and Heart. Journal of Investigative Dermatology. 2017;137:518–22.

284. Goulding M, Sterrer S, Fleming J, Balling R, Nadeau J, Moore KJ, et al. Analysis of the Pax-3 gene in the mouse mutant splotch. Genomics. 1993;17:355–63.

285. Gowen BG, Chim B, Marceau CD, Greene TT, Burr P, Gonzalez JR, et al. A forward genetic screen reveals novel independent regulators of ULBP1, an activating ligand for natural killer cells. Elife.

2015;4.

286. Granum S, Sundvold-Gjerstad V, Dai K-Z, Kolltveit KM, Hildebrand K, Huitfeldt HS, et al. Structure function analysis of SH2D2A isoforms expressed in T cells reveals a crucial role for the proline rich region encoded by SH2D2A exon 7. BMC Immunol. 2006;7:15.

287. Granzier H, Radke M, Royal J, Wu Y, Irving TC, Gotthardt M, et al. Functional genomics of chicken, mouse, and human titin supports splice diversity as an important mechanism for regulating biomechanics of striated muscle. Am J Physiol Regul Integr Comp Physiol. 2007;293:R557-567.

288. Grimwood J, Gordon LA, Olsen A, Terry A, Schmutz J, Lamerdin J, et al. The DNA sequence and biology of human chromosome 19. Nature. 2004;428:529–35.

289. Griparic L, Kanazawa T, van der Bliek AM. Regulation of the mitochondrial dynamin-like protein Opa1 by proteolytic cleavage. J Cell Biol. 2007;178:757–64.

290. Grohmann M, Hammer P, Walther M, Paulmann N, Büttner A, Eisenmenger W, et al. Alternative splicing and extensive RNA editing of human TPH2 transcripts. PLoS ONE. 2010;5:e8956.

291. Grumont RJ, Gerondakis S. Alternative splicing of RNA transcripts encoded by the murine p105 NF-kappa B gene generates I kappa B gamma isoforms with different inhibitory activities. Proc Natl Acad Sci USA. 1994;91:4367–71.

292. Guiramand J, Montmayeur JP, Ceraline J, Bhatia M, Borrelli E. Alternative splicing of the dopamine D2 receptor directs specificity of coupling to G-proteins. J Biol Chem. 1995;270:7354–8.

293. Günzel D, Stuiver M, Kausalya PJ, Haisch L, Krug SM, Rosenthal R, et al. Claudin-10 exists in six alternatively spliced isoforms that exhibit distinct localization and function. J Cell Sci. 2009;122 Pt 10:1507–17.

294. Guo B, Godzik A, Reed JC. Bcl-G, a novel pro-apoptotic member of the Bcl-2 family. J Biol Chem. 2001;276:2780–5.

295. Guo R, Li Y, Ning J, Sun D, Lin L, Liu X. HnRNP A1/A2 and SF2/ASF Regulate Alternative Splicing of Interferon Regulatory Factor-3 and Affect Immunomodulatory Functions in Human Non-Small Cell Lung Cancer Cells. PLoS One. 2013;8. doi:10.1371/journal.pone.0062729.

296. Guo R, Zheng L, Park JW, Lv R, Chen H, Jiao F, et al. BS69/ZMYND11 reads and connects histone H3.3 lysine 36 trimethylation-decorated chromatin to regulated pre-mRNA processing. Mol Cell. 2014;56:298–310.

297. Guo W, Chen D, Fan Z, Epstein HF. Differential Turnover of Myosin Chaperone UNC-45A Isoforms Increases in Metastatic Human Breast Cancer. Journal of Molecular Biology. 2011;412:365–78.

298. Guo W, Schafer S, Greaser ML, Radke MH, Liss M, Govindarajan T, et al. RBM20, a gene for hereditary cardiomyopathy, regulates titin splicing. Nat Med. 2012;18:766–73.

299. Guo Z, Yang X, Sun F, Jiang R, Linn DE, Chen H, et al. A novel androgen receptor splice variant is up-regulated during prostate cancer progression and promotes androgen depletion-resistant growth. Cancer Res. 2009;69:2305–13.

300. Guthrie CR, Murray AT, Franklin AA, Hamblin MW. Differential agonist-mediated internalization of the human 5-hydroxytryptamine 7 receptor isoforms. J Pharmacol Exp Ther. 2005;313:1003–10.

301. Hackmann K, Markoff A, Qian F, Bogdanova N, Germino GG, Pennekamp P, et al. A splice form of

polycystin-2, lacking exon 7, does not interact with polycystin-1. Hum Mol Genet. 2005;14:3249–62.

302. Hafsi H, Santos-Silva D, Courtois-Cox S, Hainaut P. Effects of Δ40p53, an isoform of p53 lacking the N-terminus, on transactivation capacity of the tumor suppressor protein p53. BMC Cancer. 2013;13:134.

303. Hagan JP, O'Neill BL, Stewart CL, Kozlov SV, Croce CM. At least ten genes define the imprinted Dlk1-Dio3 cluster on mouse chromosome 12qF1. PLoS ONE. 2009;4:e4352.

304. Hagiyama M, Ichiyanagi N, Kimura KB, Murakami Y, Ito A. Expression of a soluble isoform of cell adhesion molecule 1 in the brain and its involvement in directional neurite outgrowth. Am J Pathol. 2009;174:2278–89.

305. Hahm K, Ernst P, Lo K, Kim GS, Turck C, Smale ST. The lymphoid transcription factor LyF-1 is encoded by specific, alternatively spliced mRNAs derived from the Ikaros gene. Mol Cell Biol. 1994;14:7111–23.

306. Hai T, Yeung M-L, Wood TG, Wei Y, Yamaoka S, Gatalica Z, et al. An alternative splice product of IkappaB kinase (IKKgamma), IKKgamma-delta, differentially mediates cytokine and human T-cell leukemia virus type 1 tax-induced NF-kappaB activation. J Virol. 2006;80:4227–41.

307. Hakre S, Tussie-Luna MI, Ashworth T, Novina CD, Settleman J, Sharp PA, et al. Opposing functions of TFII-I spliced isoforms in growth factor-induced gene expression. Mol Cell. 2006;24:301–8.

308. Hamada N, Ito H, Iwamoto I, Morishita R, Tabata H, Nagata K-I. Role of the cytoplasmic isoform of RBFOX1/A2BP1 in establishing the architecture of the developing cerebral cortex. Mol Autism. 2015;6. doi:10.1186/s13229-015-0049-5.

309. Hamada N, Ito H, Nishijo T, Iwamoto I, Morishita R, Tabata H, et al. Essential role of the nuclear isoform of RBFOX1, a candidate gene for autism spectrum disorders, in the brain development. Sci Rep. 2016;6. doi:10.1038/srep30805.

310. Hamdollah Zadeh MA, Amin EM, Hoareau-Aveilla C, Domingo E, Symonds KE, Ye X, et al. Alternative splicing of TIA-1 in human colon cancer regulates VEGF isoform expression, angiogenesis, tumour growth and bevacizumab resistance. Mol Oncol. 2015;9:167–78.

311. Hamill KJ, Langbein L, Jones JCR, McLean WHI. Identification of a Novel Family of Laminin N-terminal Alternate Splice Isoforms. J Biol Chem. 2009;284:35588–96.

312. Han G-S, Carman GM. Characterization of the Human LPIN1-encoded Phosphatidate Phosphatase Isoforms. J Biol Chem. 2010;285:14628–38.

313. Han KA, Kulesz-Martin MF. Alternatively spliced p53 RNA in transformed and normal cells of different tissue types. Nucleic Acids Res. 1992;20:1979–81.

314. Han K-J, Yang Y, Xu L-G, Shu H-B. Analysis of a TIR-less splice variant of TRIF reveals an unexpected mechanism of TLR3-mediated signaling. J Biol Chem. 2010;285:12543–50.

315. Han Y, Eppinger E, Schuster IG, Weigand LU, Liang X, Kremmer E, et al. Formin-like 1 (FMNL1) is regulated by N-terminal myristoylation and induces polarized membrane blebbing. J Biol Chem. 2009;284:33409–17.

316. Handa M, Choi DS, Caldeiro RM, Messing RO, Gordon AS, Diamond I. Cloning of a novel isoform of the mouse NBMPR-sensitive equilibrative nucleoside transporter (ENT1) lacking a putative

phosphorylation site. Gene. 2001;262:301–7.

317. Hands KJ, Cuchet-Lourenco D, Everett RD, Hay RT. PML isoforms in response to arsenic: high-resolution analysis of PML body structure and degradation. J Cell Sci. 2014;127 Pt 2:365–75.

318. Hanoun N, Bureau C, Diab T, Gayet O, Dusetti N, Selves J, et al. The SV2 variant of KLF6 is down-regulated in hepatocellular carcinoma and displays anti-proliferative and pro-apoptotic functions. J Hepatol. 2010;53:880–8.

319. Hans M, Urrutia A, Deal C, Brust PF, Stauderman K, Ellis SB, et al. Structural elements in domain IV that influence biophysical and pharmacological properties of human alpha1A-containing high-voltage-activated calcium channels. Biophys J. 1999;76:1384–400.

320. Hara H, Takeda T, Yamamoto N, Furuya K, Hirose K, Kamiya T, et al. Zinc-induced modulation of SRSF6 activity alters Bim splicing to promote generation of the most potent apoptotic isoform BimS. FEBS J. 2013;280:3313–27.

321. Harada N, Yonemoto H, Yoshida M, Yamamoto H, Yin Y, Miyamoto A, et al. Alternative splicing produces a constitutively active form of human SREBP-1. Biochem Biophys Res Commun. 2008;368:820–6.

322. Harada N, Yamada Y, Tsukiyama K, Yamada C, Nakamura Y, Mukai E, et al. A novel GIP receptor splice variant influences GIP sensitivity of pancreatic beta-cells in obese mice. Am J Physiol Endocrinol Metab. 2008;294:E61-68.

323. Harazono A, Sugimoto Y, Ichikawa A, Negishi M. Enhancement of adenylate cyclase stimulation by prostaglandin E receptor EP3 subtype isoforms with different efficiencies. Biochem Biophys Res Commun. 1994;201:340–5.

324. Harms KL, Chen X. The C terminus of p53 family proteins is a cell fate determinant. Mol Cell Biol. 2005;25:2014–30.

325. Harper RW, Xu C, Soucek K, Setiadi H, Eiserich JP. A reappraisal of the genomic organization of human Nox1 and its splice variants. Arch Biochem Biophys. 2005;435:323–30.

326. Harris GM, Dodelzon K, Gong L, Gonzalez-Alegre P, Paulson HL. Splice isoforms of the polyglutamine disease protein ataxin-3 exhibit similar enzymatic yet different aggregation properties. PLoS ONE. 2010;5:e13695.

327. Harrison CB, Selemidis S, Guida E, King PT, Sobey CG, Drummond GR. NOX2β: A novel splice variant of NOX2 that regulates NADPH oxidase activity in macrophages. PLoS ONE. 2012;7:e48326.

328. Hascoet P, Chesnel F, Jouan F, Le Goff C, Couturier A, Darrigrand E, et al. The pVHL172 isoform is not a tumor suppressor and up-regulates a subset of pro-tumorigenic genes including TGFB1 and MMP13. Oncotarget. 2017.

329. Hashimoto K, Ishida E, Matsumoto S, Shibusawa N, Okada S, Monden T, et al. A liver X receptor (LXR)-beta alternative splicing variant (LXRBSV) acts as an RNA co-activator of LXR-beta. Biochem Biophys Res Commun. 2009;390:1260–5.

330. Hashimoto Y, Kurita M, Matsuoka M. Identification of soluble WSX-1 not as a dominant-negative but as an alternative functional subunit of a receptor for an anti-Alzheimer's disease rescue factor Humanin. Biochem Biophys Res Commun. 2009;389:95–9.

331. Hassler JR, Scheuner DL, Wang S, Han J, Kodali VK, Li P, et al. The IRE1α/XBP1s Pathway Is Essential for the Glucose Response and Protection of β Cells. PLoS Biol. 2015;13:e1002277.

332. Hatfield MD, Reis AMC, Obeso D, Cook JR, Thompson DM, Rao M, et al. Identification of MMS19 domains with distinct functions in NER and transcription. DNA Repair (Amst). 2006;5:914–24.

333. Hauser AD, Bergom C, Schuld NJ, Chen X, Lorimer EL, Huang J, et al. The SmgGDS splice variant SmgGDS-558 is a key promoter of tumor growth and RhoA signaling in breast cancer. Mol Cancer Res. 2014;12:130–42.

334. Hector RD, Dando O, Landsberger N, Kilstrup-Nielsen C, Kind PC, Bailey MES, et al. Characterisation of CDKL5 transcript isoforms in human and mouse. PLOS ONE. 2016;11:e0157758.

335. Heinsbroek SEM, Taylor PR, Rosas M, Willment JA, Williams DL, Gordon S, et al. Expression of functionally different dectin-1 isoforms by murine macrophages. J Immunol. 2006;176:5513–8.

336. Henderson DM, Conner SD. A novel AAK1 splice variant functions at multiple steps of the endocytic pathway. Mol Biol Cell. 2007;18:2698–706.

337. Hering S, Berjukow S, Sokolov S, Marksteiner R, Weiss RG, Kraus R, et al. Molecular determinants of inactivation in voltage-gated Ca2+ channels. J Physiol (Lond). 2000;528 Pt 2:237–49.

338. Hernández-Torres F, Rastrojo A, Aguado B. Intron retention and transcript chimerism conserved across mammals: Ly6g5b and Csnk2b-Ly6g5b as examples. BMC Genomics. 2013;14:199.

339. Hervé M-A, Buteau-Lozano H, Mourah S, Calvo F, Perrot-Applanat M. VEGF189 stimulates endothelial cells proliferation and migration in vitro and up-regulates the expression of Flk-1/KDR mRNA. Exp Cell Res. 2005;309:24–31.

340. Herzfeld T, Nolte D, Grznarova M, Hofmann A, Schultze JL, Müller U. X-linked dystonia parkinsonism syndrome (XDP, lubag): disease-specific sequence change DSC3 in TAF1/DYT3 affects genes in vesicular transport and dopamine metabolism. Hum Mol Genet. 2013;22:941–51.

341. Heyd F, Carmo-Fonseca M, Möröy T. Differential isoform expression and interaction with the P32 regulatory protein controls the subcellular localization of the splicing factor U2AF26. J Biol Chem. 2008;283:19636–45.

342. Hickey TE, Irvine CM, Dvinge H, Tarulli GA, Hanson AR, Ryan NK, et al. Expression of androgen receptor splice variants in clinical breast cancers. Oncotarget. 2015;6:44728–44.

343. Hill JL, Hardy NF, Jimenez DV, Maynard KR, Kardian AS, Pollock CJ, et al. Loss of promoter IV-driven BDNF expression impacts oscillatory activity during sleep, sensory information processing and fear regulation. Transl Psychiatry. 2016;6:e873.

344. Hinman MN, Sharma A, Luo G, Lou H. Neurofibromatosis type 1 alternative splicing is a key regulator of Ras signaling in neurons. Mol Cell Biol. 2014;34:2188–97.

345. Hmitou I, Druillennec S, Valluet A, Peyssonnaux C, Eychène A. Differential regulation of B-raf isoforms by phosphorylation and autoinhibitory mechanisms. Mol Cell Biol. 2007;27:31–43.

346. Hódi Z, Németh AL, Radnai L, Hetényi C, Schlett K, Bodor A, et al. Alternatively spliced exon B of myosin Va is essential for binding the tail-associated light chain shared by dynein. Biochemistry. 2006;45:12582–95.

347. Hofbauer SW, Krenn PW, Piñón Hofbauer J, Pucher S, Asslaber D, Egle A, et al. The AKT1 isoform plays a dominant role in the survival and chemoresistance of chronic lymphocytic leukaemia cells. Br J Haematol. 2016;172:815–9.

348. Hofer-Warbinek R, Schmid JA, Mayer H, Winsauer G, Orel L, Mueller B, et al. A highly conserved proapoptotic gene, IKIP, located next to the APAF1 gene locus, is regulated by p53. Cell Death Differ. 2004;11:1317–25.

349. Hoff AO, Catala-Lehnen P, Thomas PM, Priemel M, Rueger JM, Nasonkin I, et al. Increased bone mass is an unexpected phenotype associated with deletion of the calcitonin gene. J Clin Invest. 2002;110:1849–57.

350. Hofstetter G, Berger A, Fiegl H, Slade N, Zorić A, Holzer B, et al. Alternative splicing of p53 and p73: the novel p53 splice variant p53delta is an independent prognostic marker in ovarian cancer. Oncogene. 2010;29:1997–2004.

351. Holm F, Hellqvist E, Mason CN, Ali SA, Delos-Santos N, Barrett CL, et al. Reversion to an embryonic alternative splicing program enhances leukemia stem cell self-renewal. Proc Natl Acad Sci USA. 2015;112:15444–9.

352. Holmberg J, Clarke DL, Frisen J. Regulation of repulsion versus adhesion by different splice forms of an Eph receptor. Nature; London. 2000;408:203–6.

353. Hon H, Rucker EB, Hennighausen L, Jacob J. bcl-xL is critical for dendritic cell survival in vivo. J Immunol. 2004;173:4425–32.

354. Hong M, Zhukareva V, Vogelsberg-Ragaglia V, Wszolek Z, Reed L, Miller BI, et al. Mutation-specific functional impairments in distinct tau isoforms of hereditary FTDP-17. Science. 1998;282:1914–7.

355. Horiuchi K, Kawamura T, Iwanari H, Ohashi R, Naito M, Kodama T, et al. Identification of Wilms' Tumor 1-associating Protein Complex and Its Role in Alternative Splicing and the Cell Cycle. J Biol Chem. 2013;288:33292–302.

356. Horiuchi S, Koyanagi Y, Tanaka Y, Waki M, Matsumoto A, Zhou YW, et al. Altered interleukin-2 receptor alpha-chain is expressed in human T-cell leukaemia virus type-I-infected T-cell lines and human peripheral blood mononuclear cells of adult T-cell leukaemia patients through an alternative splicing mechanism. Immunology. 1997;91:28–34.

357. Hosoya T, Monden T, Fukabori Y, Hashimoto K, Satoh T, Kasai K, et al. A novel splice variant of the nuclear coactivator p120 functions strongly for androgen receptor: characteristic expression in prostate disease. Endocr J. 2008;55:657–65.

358. Hövelmeyer N, Wunderlich FT, Massoumi R, Jakobsen CG, Song J, Wörns MA, et al. Regulation of B cell homeostasis and activation by the tumor suppressor gene CYLD. J Exp Med. 2007;204:2615–27.

359. Howard A, Barley NF, Legon S, Walters JRF. Plasma-membrane calcium-pump isoforms in human and rat liver. Biochemical Journal. 1994;303:275–9.

360. Hu R, Dunn TA, Wei S, Isharwal S, Veltri RW, Humphreys E, et al. Ligand-independent androgen receptor variants derived from splicing of cryptic exons signify hormone-refractory prostate cancer. Cancer Res. 2009;69:16–22.

361. Hu X, Emanuel PD, Zuckerman KS. Cloning and sequencing of the cDNAs encoding two alternative splicing-derived variants of the alpha subunit of the granulocyte-macrophage colony-stimulating

factor receptor. Biochim Biophys Acta. 1994;1223:306–8.

362. Hu X, Zuckerman KS. Cloning and sequencing of an alternative splicing-derived cDNA variant of the GM-CSF receptor alpha subunit, which encodes a truncated protein. Am J Hematol. 1998;58:145–7.

363. Huang D, Pospiech H, Kesti T, Syväoja JE. Structural organization and splice variants of the POLE1 gene encoding the catalytic subunit of human DNA polymerase epsilon. Biochem J. 1999;339 (Pt 3):657–65.

364. Huang G-W, Zhang Y-L, Liao L-D, Li E-M, Xu L-Y. Natural antisense transcript TPM1-AS regulates the alternative splicing of tropomyosin I through an interaction with RNA-binding motif protein 4. The International Journal of Biochemistry & Cell Biology. 2017. doi:10.1016/j.biocel.2017.07.017.

365. Huang J, Grotzer MA, Watanabe T, Hewer E, Pietsch T, Rutkowski S, et al. Mutations in the Nijmegen breakage syndrome gene in medulloblastomas. Clin Cancer Res. 2008;14:4053–8.

366. Huang M-C, Miller AL, Wang W, Kong Y, Paul S, Goetzl EJ. Differential signaling of T cell generation of IL-4 by wild-type and short-deletion variant of type 2 G protein-coupled receptor for vasoactive intestinal peptide (VPAC2). J Immunol. 2006;176:6640–6.

367. Huang R, Huang J, Cathcart H, Smith S, Poduslo SE. Genetic variants in brain-derived neurotrophic factor associated with Alzheimer's disease. J Med Genet. 2007;44:e66.

368. Huang TY, Michael S, Xu T, Sarkeshik A, Moresco JJ, Yates JR, et al. A novel Rac1 GAP splice variant relays poly-Ub accumulation signals to mediate Rac1 inactivation. Mol Biol Cell. 2013;24:194–209.

369. Huang Y, Kapere Ochieng J, Kempen MB, Munck AB, Swagemakers S, van Ijcken W, et al. Hypoxia inducible factor 3α plays a critical role in alveolarization and distal epithelial cell differentiation during mouse lung development. PLoS ONE. 2013;8:e57695.

370. Huang Y, Chen X, Chen N, Nie L, Xu M, Zhou Q. Expression and prognostic significance of survivin splice variants in diffusely infiltrating astrocytoma. J Clin Pathol. 2011;64:953–9.

371. Hurt KJ, Sezen SF, Champion HC, Crone JK, Palese MA, Huang PL, et al. Alternatively spliced neuronal nitric oxide synthase mediates penile erection. Proc Natl Acad Sci USA. 2006;103:3440–3.

372. Hutton M, Lendon CL, Rizzu P, Baker M, Froelich S, Houlden H, et al. Association of missense and 5'-splice-site mutations in tau with the inherited dementia FTDP-17. Nature. 1998;393:702–5.

373. Hymowitz SG, Compaan DM, Yan M, Wallweber HJA, Dixit VM, Starovasnik MA, et al. The crystal structures of EDA-A1 and EDA-A2: splice variants with distinct receptor specificity. Structure. 2003;11:1513–20.

374. Hymowitz SG, Patel DR, Wallweber HJA, Runyon S, Yan M, Yin J, et al. Structures of APRIL-receptor complexes: like BCMA, TACI employs only a single cysteine-rich domain for high affinity ligand binding. J Biol Chem. 2005;280:7218–27.

375. Iijima T, Wu K, Witte H, Hanno-Iijima Y, Glatter T, Richard S, et al. SAM68 regulates neuronal activity-dependent alternative splicing of neurexin-1. Cell. 2011;147:1601–14.

376. Ikeda M, Inoue F, Ohkoshi K, Yokoyama S, Tatemizo A, Tokunaga T, et al. B-box and SPRY domain containing protein (BSPRY) is associated with the maintenance of mouse embryonic stem cell

pluripotency and early embryonic development. J Reprod Dev. 2012;58:691–9.

377. Imamura R, Masuda ES, Naito Y, Imai S, Fujino T, Takano T, et al. Carboxyl-terminal 15-amino acid sequence of NFATx1 Is possibly created by tissue-specific splicing and is essential for transactivation activity in T Cells. The Journal of Immunology. 1998;161:3455–63.

378. Indraccolo S, Minuzzo S, Zamarchi R, Calderazzo F, Piovan E, Amadori A. Alternatively spliced forms of Igalpha and Igbeta prevent B cell receptor expression on the cell surface. Eur J Immunol. 2002;32:1530–40.

379. Ingelsson M, Ramasamy K, Russ C, Freeman SH, Orne J, Raju S, et al. Increase in the relative expression of tau with four microtubule binding repeat regions in frontotemporal lobar degeneration and progressive supranuclear palsy brains. Acta Neuropathol. 2007;114:471–9.

380. Ishizaka A, Mizutani T, Kobayashi K, Tando T, Sakurai K, Fujiwara T, et al. Double plant homeodomain (PHD) finger proteins DPF3a and -3b are required as transcriptional co-activators in SWI/SNF complex-dependent activation of NF-κB RelA/p50 heterodimer. J Biol Chem. 2012;287:11924–33.

381. Israel DD, Regan JW. EP(3) prostanoid receptor isoforms utilize distinct mechanisms to regulate ERK 1/2 activation. Biochim Biophys Acta. 2009;1791:238–45.

382. Itoh M, Nagatomo K, Kubo Y, Saitoh O. Alternative splicing of RGS8 gene changes the binding property to the M1 muscarinic receptor to confer receptor type-specific Gq regulation. J Neurochem. 2006;99:1505–16.

383. Iwasaki T, Koibuchi N, Chin WW. Synovial sarcoma translocation (SYT) encodes a nuclear receptor coactivator. Endocrinology. 2005;146:3892–9.

384. Izaguirre DI, Zhu W, Hai T, Cheung HC, Krahe R, Cote GJ. PTBP1-dependent regulation of USP5 alternative RNA splicing plays a role in glioblastoma tumorigenesis. Mol Carcinog. 2012;51:895–906.

385. Izquierdo JM, Valcárcel J. Fas-activated serine/threonine kinase (FAST K) synergizes with TIA-1/TIAR proteins to regulate Fas alternative splicing. J Biol Chem. 2007;282:1539–43.

386. Jackson KA, Helston RM, McKay JA, O'Neill ED, Mathers JC, Ford D. Splice variants of the human zinc transporter ZnT5 (SLC30A5) are differentially localized and regulated by zinc through transcription and mRNA stability. J Biol Chem. 2007;282:10423–31.

387. Jackson P, Rowe A, Grimm M-O. An alternatively spliced KAI1 mRNA is expressed at low levels in human bladder cancers and bladder cancer cell lines and is not associated with invasive behaviour. Oncol Rep. 2007;18:1357–63.

388. Jacob AG, O'Brien D, Singh RK, Comiskey DF, Littleton RM, Mohammad F, et al. Stress-induced isoforms of MDM2 and MDM4 correlate with high-grade disease and an altered splicing network in pediatric rhabdomyosarcoma. Neoplasia. 2013;15:1049–63.

389. Jacob AG, Singh RK, Comiskey DF, Rouhier MF, Mohammad F, Bebee TW, et al. Stress-induced alternative splice forms of MDM2 and MDMX modulate the p53-pathway in distinct ways. PLoS ONE. 2014;9:e104444.

390. Jagannath A, Hughes S, Abdelgany A, Pothecary CA, Di Pretoro S, Pires SS, et al. Isoforms of Melanopsin Mediate Different Behavioral Responses to Light. Curr Biol. 2015;25:2430–4.

391. Jagla M, Fève M, Kessler P, Lapouge G, Erdmann E, Serra S, et al. A splicing variant of the androgen receptor detected in a metastatic prostate cancer exhibits exclusively cytoplasmic actions. Endocrinology. 2007;148:4334–43.

392. Jain BP, Pandey S, Saleem N, Tanti GK, Mishra S, Goswami SK. SG2NA is a regulator of endoplasmic reticulum (ER) homeostasis as its depletion leads to ER stress. Cell Stress Chaperones. 2017.

393. Jakubowska A, Górski B, Byrski T, Huzarski T, Gronwald J, Menkiszak J, et al. Detection of germline mutations in the BRCA1 gene by RNA-based sequencing. Hum Mutat. 2001;18:149–56.

394. Jannatipour M, Dion P, Khan S, Jindal H, Fan X, Laganière J, et al. Schwannomin isoform-1 interacts with syntenin via PDZ domains. J Biol Chem. 2001;276:33093–100.

395. Jaskolski F, Normand E, Mulle C, Coussen F. Differential trafficking of GluR7 kainate receptor subunit splice variants. J Biol Chem. 2005;280:22968–76.

396. Jenkins PM, Kim N, Jones SL, Tseng WC, Svitkina TM, Yin HH, et al. Giant ankyrin-G: a critical innovation in vertebrate evolution of fast and integrated neuronal signaling. Proc Natl Acad Sci USA. 2015;112:957–64.

397. Jensen LE, Whitehead AS. IRAK1b, a novel alternative splice variant of interleukin-1 receptor-associated kinase (IRAK), mediates interleukin-1 signaling and has prolonged stability. J Biol Chem. 2001;276:29037–44.

398. Jeong M-H, Bae J, Kim W-H, Yoo S-M, Kim J-W, Song PI, et al. p19ras interacts with and activates p73 by involving the MDM2 protein. J Biol Chem. 2006;281:8707–15.

399. Jeong Y, Du R, Zhu X, Yin S, Wang J, Cui H, et al. Histone deacetylase isoforms regulate innate immune responses by deacetylating mitogen-activated protein kinase phosphatase-1. J Leukoc Biol. 2014;95:651–9.

400. Jiang Z, Tang H, Havlioglu N, Zhang X, Stamm S, Yan R, et al. Mutations in tau gene exon 10 associated with FTDP-17 alter the activity of an exonic splicing enhancer to interact with Tra2 beta. J Biol Chem. 2003;278:18997–9007.

401. Jin C, Woodward JJ. Effects of 8 different NR1 splice variants on the ethanol inhibition of recombinant NMDA receptors. Alcohol Clin Exp Res. 2006;30:673–9.

402. Jones DC, Roghanian A, Brown DP, Chang C, Allen RL, Trowsdale J, et al. Alternative mRNA splicing creates transcripts encoding soluble proteins from most LILR genes. Eur J Immunol. 2009;39:3195–206.

403. Jones PF, Jakubowicz T, Hemmings BA. Molecular cloning of a second form of rac protein kinase. Cell Regul. 1991;2:1001–9.

404. Jonsen MD, Duval DL, Gutierrez-Hartmann A. The 26-amino acid beta-motif of the Pit-1beta transcription factor is a dominant and independent repressor domain. Mol Endocrinol. 2009;23:1371–84.

405. Józsi M, Richter H, Löschmann I, Skerka C, Buck F, Beisiegel U, et al. FHR-4A: a new factor H-related protein is encoded by the human FHR-4 gene. Eur J Hum Genet. 2005;13:321–9.

406. Juan WC, Roca X, Ong ST. Identification of cis-acting elements and splicing factors involved in the regulation of BIM Pre-mRNA splicing. PLoS ONE. 2014;9:e95210.

407. Kabuss R, Ashikov A, Oelmann S, Gerardy-Schahn R, Bakker H. Endoplasmic reticulum retention of the large splice variant of the UDP-galactose transporter is caused by a dilysine motif. Glycobiology. 2005;15:905–11.

408. Kaczmarski W, Barua M, Mazur-Kolecka B, Frackowiak J, Dowjat W, Mehta P, et al. Intracellular distribution of differentially phosphorylated dual-specificity tyrosine phosphorylation-regulated kinase 1A (DYRK1A). Journal of Neuroscience Research. 2014;92:162–73.

409. Kajiwara T, Matsushita K, Itoga S, Tamura M, Tanaka N, Tomonaga T, et al. SAP155-mediated c-myc suppressor far-upstream element-binding protein-interacting repressor splicing variants are activated in colon cancer tissues. Cancer Sci. 2013;104:149–56.

410. Kalisiak K, Kuliński T, Tomecki R, Cysewski D, Pietras Z, Chlebowski A, et al. A short splicing isoform of HBS1L links the cytoplasmic exosome and SKI complexes in humans. Nucleic Acids Research. 2017;45:2068–2080.

411. Kaneko M, Alvarez-Manilla G, Kamar M, Lee I, Lee JK, Troupe K, et al. A novel beta(1,6)-N-acetylglucosaminyltransferase V (GnT-VB)(1). FEBS Lett. 2003;554:515–9.

412. Kaneko S, Cooper CB, Nishioka N, Yamasaki H, Suzuki A, Jarvis SE, et al. Identification and characterization of novel human Ca(v)2.2 (alpha 1B) calcium channel variants lacking the synaptic protein interaction site. J Neurosci. 2002;22:82–92.

413. Kanezaki R, Toki T, Yokoyama M, Yomogida K, Sugiyama K, Yamamoto M, et al. Transcription factor BACH1 is recruited to the nucleus by its novel alternative spliced isoform. J Biol Chem. 2001;276:7278–84.

414. Kang HC, Chae JH, Lee YH, Park M-A, Shin JH, Kim S-H, et al. Erythroid cell-specific alpha-globin gene regulation by the CP2 transcription factor family. Mol Cell Biol. 2005;25:6005–20.

415. Kapp K, Siemens J, Weyrich P, Schulz JB, Häring H-U, Lammers R. Extracellular domain splice variants of a transforming protein tyrosine phosphatase α mutant differentially activate Src-kinase dependent focus formation. Genes to Cells. 2007;12:63–73.

416. Kappler M, Rot S, Taubert H, Greither T, Bartel F, Dellas K, et al. The effects of knockdown of wild-type survivin, survivin-2B or survivin-delta3 on the radiosensitization in a soft tissue sarcoma cells in vitro under different oxygen conditions. Cancer Gene Ther. 2007;14:994–1001.

417. Kar A, Havlioglu N, Tarn W-Y, Wu JY. RBM4 interacts with an intronic element and stimulates tau exon 10 inclusion. J Biol Chem. 2006;281:24479–88.

418. Karas RH, Baur WE, van Eickles M, Mendelsohn ME. Human vascular smooth muscle cells express an estrogen receptor isoform. FEBS Letters. 1995;377:103–8.

419. Karimi MA, Aguilar O, Zou B, Bachmann MH, Carlyle JR, Baldwin CL, et al. A truncated human NKG2D splice isoform negatively regulates NKG2D-mediated function. J Immunol. 2014;193:2764–71.

420. Karpova AY, Ronco LV, Howley PM. Functional characterization of interferon regulatory factor 3a (IRF-3a), an alternative splice isoform of IRF-3. Mol Cell Biol. 2001;21:4169–76.

421. Kashkin KN, Khlgatian SV, Gurova OV, Kuprash DV, Nedospasov SA. New mutations in the human p53 gene--a regulator of the cell cycle and carcinogenesis. Biochemistry Mosc. 2007;72:282–92.

422. Kaul R, Mukherjee S, Ahmed F, Bhat MK, Chhipa R, Galande S, et al. Direct interaction with and

activation of p53 by SMAR1 retards cell-cycle progression at G2/M phase and delays tumor growth in mice. Int J Cancer. 2003;103:606–15.

423. Kawamura K, Watanabe K, Suzuki T, Yamakawa T, Kamiyama T, Nakagawa H, et al. cDNA cloning and expression of a novel human desmocollin. J Biol Chem. 1994;269:26295–302.

424. Kayagaki N, Warming S, Lamkanfi M, Walle LV, Louie S, Dong J, et al. Non-canonical inflammasome activation targets caspase-11. Nature. 2011;479:117–21.

425. Keats JJ, Maxwell CA, Taylor BJ, Hendzel MJ, Chesi M, Bergsagel PL, et al. Overexpression of transcripts originating from the MMSET locus characterizes all t(4;14)(p16;q32)-positive multiple myeloma patients. Blood. 2005;105:4060–9.

426. Kern J, Bauer M, Rychli K, Wojta J, Ritsch A, Gastl G, et al. Alternative splicing of vasohibin-1 generates an inhibitor of endothelial cell proliferation, migration, and capillary tube formation. Arterioscler Thromb Vasc Biol. 2008;28:478–84.

427. Kerr B, Soto C J, Saez M, Abrams A, Walz K, Young JI. Transgenic complementation of MeCP2 deficiency: phenotypic rescue of Mecp2-null mice by isoform-specific transgenes. Eur J Hum Genet. 2012;20:69–76.

428. Kestler DP, Agarwal S, Cobb J, Goldstein KM, Hall RE. Detection and analysis of an alternatively spliced isoform of interleukin-6 mRNA in peripheral blood mononuclear cells. Blood. 1995;86:4559–67.

429. Khankin EV, Mutter WP, Tamez H, Yuan H-T, Karumanchi SA, Thadhani R. Soluble erythropoietin receptor contributes to erythropoietin resistance in end-stage renal disease. PLoS ONE. 2010;5:e9246.

430. Khurana S, Chakraborty S, Zhao X, Liu Y, Guan D, Lam M, et al. Identification of a Novel LXXLL Motif in α-Actinin 4-spliced Isoform That Is Critical for Its Interaction with Estrogen Receptor α and Co-activators. J Biol Chem. 2012;287:35418–29.

431. Kile BT, Viney EM, Willson TA, Brodnicki TC, Cancilla MR, Herlihy AS, et al. Cloning and characterization of the genes encoding the ankyrin repeat and SOCS box-containing proteins Asb-1, Asb-2, Asb-3 and Asb-4. Gene. 2000;258:31–41.

432. Kim C-H, Kim Y-D, Choi E-K, Kim H-R, Na B-R, Im S-H, et al. Nuclear Speckle-related Protein 70 Binds to Serine/Arginine-rich Splicing Factors 1 and 2 via an Arginine/Serine-like Region and Counteracts Their Alternative Splicing Activity. J Biol Chem. 2016;291:6169–81.

433. Kim CJ, Nishi K, Isono T, Okuyama Y, Tambe Y, Okada Y, et al. Cyclin D1b variant promotes cell invasiveness independent of binding to CDK4 in human bladder cancer cells. Mol Carcinog. 2009;48:953–64.

434. Kim DK, Catterall WA. Ca2+-dependent and -independent interactions of the isoforms of the alpha1A subunit of brain Ca2+ channels with presynaptic SNARE proteins. Proc Natl Acad Sci USA. 1997;94:14782–6.

435. Kim D-J, Oh B, Kim Y-Y. Splicing factor ASF/SF2 and transcription factor PPAR-gamma cooperate to directly regulate transcription of uncoupling protein-3. Biochem Biophys Res Commun. 2009;378:877–82.

436. Kim EY, Chiu Y-H, Dryer SE. Neph1 regulates steady-state surface expression of Slo1 Ca2+-activated K+ channels: different effects in embryonic neurons and podocytes. Am J Physiol Cell

Physiol. 2009;297:C1379–88.

437. Kim HS, Jung G. Reactive oxygen species increase HEPN1 expression via activation of the XBP1 transcription factor. FEBS Lett. 2014;588:4413–21.

438. Kim HJ, Woo IS, Kang ES, Eun SY, Kim HJ, Lee JH, et al. Identification of a truncated alternative splicing variant of human PPARgamma1 that exhibits dominant negative activity. Biochem Biophys Res Commun. 2006;347:698–706.

439. Kim J-H, Bae J. MCL-1ES induces MCL-1L-dependent BAX- and BAK-independent mitochondrial apoptosis. PLoS ONE. 2013;8:e79626.

440. Kim J-E, Han JM, Park CR, Shin K-J, Ahn C, Seong JY, et al. Splicing variants of the orphan G-protein-coupled receptor GPR56 regulate the activity of transcription factors associated with tumorigenesis. J Cancer Res Clin Oncol. 2010;136:47–53.

441. Kim J, Sunagawa M, Kobayashi S, Shin T, Takayama C. Developmental localization of calcitonin gene-related peptide in dorsal sensory axons and ventral motor neurons of mouse cervical spinal cord. Neurosci Res. 2016;105:42–8.

442. Kim JY, Lee KH, Shim MS, Shin H, Xu X-M, Carlson BA, et al. Human selenophosphate synthetase 1 has five splice variants with unique interactions, subcellular localizations and expression patterns. Biochem Biophys Res Commun. 2010;397:53–8.

443. Kim K, Ryu J-H, Park J-W, Kim M-S, Chun Y-S. Induction of a SSAT isoform in response to hypoxia or iron deficiency and its protective effects on cell death. Biochem Biophys Res Commun. 2005;331:78–85.

444. Kim Y-D, Lee J-Y, Oh K-M, Araki M, Araki K, Yamamura K, et al. NSrp70 is a novel nuclear speckle-related protein that modulates alternative pre-mRNA splicing in vivo. Nucleic Acids Res. 2011;39:4300–14.

445. Kimura A, Kitamura K, Ailiken G, Satoh M, Minamoto T, Tanaka N, et al. FIR haplodeficiency promotes splicing to pyruvate kinase M2 in mice thymic lymphoma tissues revealed by six-plex tandem mass tag quantitative proteomic analysis. Oncotarget. 2017.

446. Kimura T, Lueck JD, Harvey PJ, Pace SM, Ikemoto N, Casarotto MG, et al. Alternative splicing of RyR1 alters the efficacy of skeletal EC coupling. Cell Calcium. 2009;45:264–74.

447. Kiok K, Sun H, Clancy H, Bose S, Kluz T, Wu F, et al. Liprin-α4 Is Required for Nickel Induced Receptor Protein Tyrosine Phosphatase-Leukocyte Antigen Related Receptor F (RPTP-LAR) Activity. PLOS ONE. 2011;6:e22764.

448. Kitayama S, Morita K, Dohi T. Functional characterization of the splicing variants of human norepinephrine transporter. Neurosci Lett. 2001;312:108–12.

449. Kleino I, Ortiz RM, Yritys M, Huovila A-PJ, Saksela K. Alternative splicing of ADAM15 regulates its interactions with cellular SH3 proteins. J Cell Biochem. 2009;108:877–85.

450. Ko J-K, Lee M-J, Cho S-H, Cho J-A, Lee B-Y, Koh JS, et al. Bfl-1S, a novel alternative splice variant of Bfl-1, localizes in the nucleus via its C-terminus and prevents cell death. Oncogene. 2003;22:2457–65.

451. Koch A, Hatina J, Rieder H, Seifert H-H, Huckenbeck W, Jankowiak F, et al. Discovery of TP53 splice variants in two novel papillary urothelial cancer cell lines. Cell Oncol (Dordr). 2012;35:243–

57.

452. Koch S, Strasser V, Hauser C, Fasching D, Brandes C, Bajari TM, et al. A secreted soluble form of ApoE receptor 2 acts as a dominant-negative receptor and inhibits Reelin signaling. EMBO J. 2002;21:5996–6004.

453. Koch T, Schulz S, Pfeiffer M, Klutzny M, Schröder H, Kahl E, et al. C-terminal splice variants of the mouse mu-opioid receptor differ in morphine-induced internalization and receptor resensitization. J Biol Chem. 2001;276:31408–14.

454. Kojima S, Hyakutake A, Koshikawa N, Nakagawara A, Takenaga K. MCL-1V, a novel mouse antiapoptotic MCL-1 variant, generated by RNA splicing at a non-canonical splicing pair. Biochem Biophys Res Commun. 2010;391:492–7.

455. Kojo H, Tajima K, Fukagawa M, Isogai T, Nishimura S. A novel estrogen receptor-related protein gamma splice variant lacking a DNA binding domain exon modulates transcriptional activity of a moderate range of nuclear receptors. J Steroid Biochem Mol Biol. 2006;98:181–92.

456. Koli K, Saharinen J, Kärkkäinen M, Keski-Oja J. Novel non-TGF-beta-binding splice variant of LTBP-4 in human cells and tissues provides means to decrease TGF-beta deposition. J Cell Sci. 2001;114 Pt 15:2869–78.

457. Kolmerer B, Olivieri N, Witt CC, Herrmann BG, Labeit S. Genomic organization of M line titin and its tissue-specific expression in two distinct isoforms. J Mol Biol. 1996;256:556–63.

458. Kontos CK, Scorilas A. Molecular cloning of novel alternatively spliced variants of BCL2L12, a new member of the BCL2 gene family, and their expression analysis in cancer cells. Gene. 2012;505:153–66.

459. Kontrogianni-Konstantopoulos A, Frye CS, Benz EJ, Huang SC. The prototypical 4.1R-10-kDa domain and the 4.1g-10-kDa paralog mediate fodrin-actin complex formation. J Biol Chem. 2001;276:20679–87.

460. Kordeli E, Lambert S, Bennett V. AnkyrinG. A new ankyrin gene with neural-specific isoforms localized at the axonal initial segment and node of Ranvier. J Biol Chem. 1995;270:2352–9.

461. Korovkina VP, Fergus DJ, Holdiman AJ, England SK. Characterization of a novel 132-bp exon of the human maxi-K channel. Am J Physiol, Cell Physiol. 2001;281:C361-367.

462. Korvatska O, Strand NS, Berndt JD, Strovas T, Chen D-H, Leverenz JB, et al. Altered splicing of ATP6AP2 causes X-linked parkinsonism with spasticity (XPDS). Hum Mol Genet. 2013;22:3259–68.

463. Kozmik Z, Kurzbauer R, Dörfler P, Busslinger M. Alternative splicing of Pax-8 gene transcripts is developmentally regulated and generates isoforms with different transactivation properties. Mol Cell Biol. 1993;13:6024–35.

464. Krause WC, Shafi AA, Nakka M, Weigel NL. Androgen receptor and its splice variant, AR-V7, differentially regulate FOXA1 sensitive genes in LNCaP prostate cancer cells. Int J Biochem Cell Biol. 2014;54:49–59.

465. Kravchenko IV, Furalyov VA, Chatziefthimiou S, Wilmanns M, Popov VO. Induction of insulin-like growth factor 1 splice forms by subfragments of myofibrillar proteins. Mol Cell Endocrinol. 2015;399:69–77.

466. Kreis P, Rousseau V, Thévenot E, Combeau G, Barnier J-V. The four mammalian splice variants encoded by the p21-activated kinase 3 gene have different biological properties. J Neurochem. 2008;106:1184–97.

467. Krick R, Jakubiczka S, Arnemann J. Expression, alternative splicing and haplotype analysis of transcribed testis specific protein (TSPY) genes. Gene. 2003;302:11–9.

468. Krieg A, Le Negrate G, Reed JC. RIP2-beta: a novel alternative mRNA splice variant of the receptor interacting protein kinase RIP2. Mol Immunol. 2009;46:1163–70.

469. Krovetz HS, Helton TD, Crews AL, Horne WA. C-terminal alternative splicing changes the gating properties of a human spinal cord calcium channel alpha 1A subunit. J Neurosci. 2000;20:7564–70.

470. Kubarek Ł, Kozłowska A, Przybylski M, Lianeri M, Jagodzinski PP. Down-regulation of CXCR4 expression by tamoxifen is associated with DNA methyltransferase 3B up-regulation in MCF-7 breast cancer cells. Biomed Pharmacother. 2009;63:586–91.

471. Kubo K-I, Tomita K, Uto A, Kuroda K, Seshadri S, Cohen J, et al. Migration defects by DISC1 knockdown in C57BL/6, 129X1/SvJ, and ICR strains via in utero gene transfer and virus-mediated RNAi. Biochem Biophys Res Commun. 2010;400:631–7.

472. Kumanogoh H, Asami J, Nakamura S, Inoue T. Balanced expression of various TrkB receptor isoforms from the Ntrk2 gene locus in the mouse nervous system. Mol Cell Neurosci. 2008;39:465–77.

473. Kumbrink J, Soni S, Laumbacher B, Loesch B, Kirsch KH. Identification of Novel Crk-associated Substrate (p130Cas) Variants with Functionally Distinct Focal Adhesion Kinase Binding Activities. J Biol Chem. 2015;290:12247–55.

474. Kustatscher G, Hothorn M, Pugieux C, Scheffzek K, Ladurner AG. Splicing regulates NAD metabolite binding to histone macroH2A. Nat Struct Mol Biol. 2005;12:624–5.

475. Kuwano Y, Nishida K, Kajita K, Satake Y, Akaike Y, Fujita K, et al. Transformer 2β and miR-204 regulate apoptosis through competitive binding to 3' UTR of BCL2 mRNA. Cell Death Differ. 2015;22:815–25.

476. Kyriazis GA, Belal C, Madan M, Taylor DG, Wang J, Wei Z, et al. Stress-induced switch in Numb isoforms enhances Notch-dependent expression of subtype-specific transient receptor potential channel. J Biol Chem. 2010;285:6811–25.

477. La Cognata V, Maugeri G, D'Amico AG, Saccone S, Federico C, Cavallaro S, et al. Differential expression of PARK2 splice isoforms in an in vitro model of dopaminergic-like neurons exposed to toxic insults mimicking Parkinson's disease. J Cell Biochem. :n/a-n/a.

478. Labeit S, Lahmers S, Burkart C, Fong C, McNabb M, Witt S, et al. Expression of distinct classes of titin isoforms in striated and smooth muscles by alternative splicing, and their conserved interaction with filamins. J Mol Biol. 2006;362:664–81.

479. Lacovich V, Espindola SL, Alloatti M, Devoto VP, Cromberg LE, Čarná ME, et al. Tau Isoforms Imbalance Impairs the Axonal Transport of the Amyloid Precursor Protein in Human Neurons. J Neurosci. 2017;37:58–69.

480. Laffin B, Wellberg E, Kwak H-I, Burghardt RC, Metz RP, Gustafson T, et al. Loss of singleminded-2s in the mouse mammary gland induces an epithelial-mesenchymal transition associated with up-

regulation of slug and matrix metalloprotease 2. Mol Cell Biol. 2008;28:1936–46.

481. Laguri C, Sadir R, Rueda P, Baleux F, Gans P, Arenzana-Seisdedos F, et al. The novel CXCL12gamma isoform encodes an unstructured cationic domain which regulates bioactivity and interaction with both glycosaminoglycans and CXCR4. PLoS ONE. 2007;2:e1110.

482. Laity JH, Chung J, Dyson HJ, Wright PE. Alternative splicing of Wilms' tumor suppressor protein modulates DNA binding activity through isoform-specific DNA-induced conformational changes. Biochemistry. 2000;39:5341–8.

483. Laity JH, Dyson HJ, Wright PE. Molecular basis for modulation of biological function by alternate splicing of the Wilms' tumor suppressor protein. Proc Natl Acad Sci USA. 2000;97:11932–5.

484. Lapointe NE, Horowitz PM, Guillozet-Bongaarts AL, Silva A, Andreadis A, Binder LI. Tau 6D and 6P isoforms inhibit polymerization of full-length tau in vitro. Biochemistry. 2009;48:12290–7.

485. Larsson SH, Charlieu JP, Miyagawa K, Engelkamp D, Rassoulzadegan M, Ross A, et al. Subnuclear localization of WT1 in splicing or transcription factor domains is regulated by alternative splicing. Cell. 1995;81:391–401.

486. Lasagni L, Francalanci M, Annunziato F, Lazzeri E, Giannini S, Cosmi L, et al. An alternatively spliced variant of CXCR3 mediates the inhibition of endothelial cell growth induced by IP-10, Mig, and I-TAC, and acts as functional receptor for platelet factor 4. J Exp Med. 2003;197:1537–49.

487. Lau JY, Oliver BG, Baraket M, Beckett EL, Hansbro NG, Moir LM, et al. Fibulin-1 Is Increased in Asthma – A Novel Mediator of Airway Remodeling? PLOS ONE. 2010;5:e13360.

488. Lauffart B, Gangisetty O, Still IH. Molecular cloning, genomic structure and interactions of the putative breast tumor suppressor TACC2. Genomics. 2003;81:192–201.

489. Laurentino SS, Pinto PIS, Tomás J, Cavaco JE, Sousa M, Barros A, et al. Identification of androgen receptor variants in testis from humans and other vertebrates. Andrologia. 2013;45:187–94.

490. Lauriat TL, Richler E, McInnes LA. A quantitative regional expression profile of EAAT2 known and novel splice variants reopens the question of aberrant EAAT2 splicing in disease. Neurochem Int. 2007;50:271–80.

491. Laursen KB, Kashyap V, Scandura J, Gudas LJ. An alternative retinoic acid-responsive Stra6 promoter regulated in response to retinol deficiency. J Biol Chem. 2015;290:4356–66.

492. Lee AW, Champagne N, Wang X, Su X-D, Goodyer C, Leblanc AC. Alternatively spliced caspase-6B isoform inhibits the activation of caspase-6A. J Biol Chem. 2010;285:31974–84.

493. Lee EJ, Kim JM, Lee MK, Jameson JL. Splice variants of the forkhead box protein AFX exhibit dominant negative activity and inhibit AFXalpha-mediated tumor cell apoptosis. PLoS ONE. 2008;3:e2743.

494. Lee H, Dean C, Isacoff E. Alternative splicing of neuroligin regulates the rate of presynaptic differentiation. J Neurosci. 2010;30:11435–46.

495. Lee J-H, Gao CF, Lee CC, Kim MD, Vande Woude GF. An alternatively spliced form of Met receptor is tumorigenic. Exp Mol Med. 2006;38:565–73.

496. Lee J-A, Damianov A, Lin C-H, Fontes M, Parikshak NN, Anderson ES, et al. Cytoplasmic Rbfox1 regulates the expression of synaptic and autism-related genes. Neuron. 2016;89:113–28.

497. Lee J-A, Tang Z-Z, Black DL. An inducible change in Fox-1/A2BP1 splicing modulates the alternative splicing of downstream neuronal target exons. Genes Dev. 2009;23:2284–93.

498. Lee J-L, Wang M-J, Sudhir P-R, Chen G-D, Chi C-W, Chen J-Y. Osteopontin promotes integrin activation through outside-in and inside-out mechanisms: OPN-CD44V interaction enhances survival in gastrointestinal cancer cells. Cancer Res. 2007;67:2089–97.

499. Lee KPK, Dey M, Neculai D, Cao C, Dever TE, Sicheri F. Structure of the dual enzyme Ire1 reveals the basis for catalysis and regulation in nonconventional RNA splicing. Cell. 2008;132:89–100.

500. Lee YM, Kim HS, Choi J-H, Choi J-K, Joo YM, Ahn S-J, et al. Knockdown of Archvillin by siRNA Inhibits Myofibril Assembly in Cultured Skeletal Myoblast. Biomedical Science Letters. 2007. http://www.dbpia.co.kr. Accessed 14 Jul 2017.

501. Lee Y-J, Hwang I-S, Lee Y-J, Lee C-H, Kim S-H, Nam H-S, et al. Knockdown of Bcl-xL Enhances Growth-Inhibiting and Apoptosis-Inducing Effects of Resveratrol and Clofarabine in Malignant Mesothelioma H-2452 Cells. J Korean Med Sci. 2014;29:1464–72.

502. Leeman JR, Weniger MA, Barth TF, Gilmore TD. Deletion analysis and alternative splicing define a transactivation inhibitory domain in human oncoprotein REL. Oncogene. 2008;27:6770–81.

503. Leff SE, Evans RM, Rosenfeld MG. Splice commitment dictates neuron-specific alternative RNA processing in calcitonin/CGRP gene expression. Cell. 1987;48:517–24.

504. Lemin AJ, Saleki K, van Lith M, Benham AM. Activation of the unfolded protein response and alternative splicing of ATF6alpha in HLA-B27 positive lymphocytes. FEBS Lett. 2007;581:1819–24.

505. Lenos K, Grawenda AM, Lodder K, Kuijjer ML, Teunisse AFAS, Repapi E, et al. Alternate splicing of the p53 inhibitor HDMX offers a superior prognostic biomarker than p53 mutation in human cancer. Cancer Res. 2012;72:4074–84.

506. Leroy O, Wang J, Maurage C-A, Parent M, Cooper T, Buée L, et al. Brain-specific change in alternative splicing of Tau exon 6 in myotonic dystrophy type 1. Biochim Biophys Acta. 2006;1762:460–7.

507. Leu S, Lin Y-M, Wu C-H, Ouyang P. Loss of Pnn expression results in mouse early embryonic lethality and cellular apoptosis through SRSF1-mediated alternative expression of Bcl-xS and ICAD. J Cell Sci. 2012;125:3164–72.

508. Leung E, Hong J, Fraser A, Krissansen GW. Splicing of NOD2 (CARD15) RNA transcripts. Mol Immunol. 2007;44:284–94.

509. Leung SW, Apponi LH, Cornejo OE, Kitchen CM, Valentini SR, Pavlath GK, et al. Splice Variants of the Human ZC3H14 Gene Generate Multiple Isoforms of a Zinc Finger Polyadenosine RNA Binding Protein. Gene. 2009;439:71–8.

510. Lévêque C, Marsaud V, Renoir J-M, Sola B. Alternative cyclin D1 forms a and b have different biological functions in the cell cycle of B lymphocytes. Exp Cell Res. 2007;313:2719–29.

511. Levitin F, Baruch A, Weiss M, Stiegman K, Hartmann M-L, Yoeli-Lerner M, et al. A novel protein derived from the MUC1 gene by alternative splicing and frameshifting. J Biol Chem. 2005;280:10655–63.

512. Levy SF, Leboeuf AC, Massie MR, Jordan MA, Wilson L, Feinstein SC. Three- and four-repeat tau

regulate the dynamic instability of two distinct microtubule subpopulations in qualitatively different manners. Implications for neurodegeneration. J Biol Chem. 2005;280:13520–8.

513. Li D, Jin C, Yin C, Zhang Y, Pang B, Tian L, et al. An alternative splice form of CMTM8 induces apoptosis. Int J Biochem Cell Biol. 2007;39:2107–19.

514. Li H, Hou S, Hao T, Azam S, Liu C, Shi L, et al. HuR antagonizes the effect of an intronic pyrimidine-rich sequence in regulating WT1 +/-KTS isoforms. RNA Biol. 2015;12:1364–71.

515. Li LC, Sheng JR, Mulherkar N, Prabhakar BS, Meriggioli MN. Regulation of apoptosis and caspase-8 expression in neuroblastoma cells by isoforms of the IG20 gene. Cancer Res. 2008;68:7352–61.

516. Li N, Richard S. Sam68 functions as a transcriptional coactivator of the p53 tumor suppressor. Nucleic Acids Res. 2016;44:8726–41.

517. Li Q, Dai Y, Guo L, Liu Y, Hao C, Wu G, et al. Polycystin-2 associates with tropomyosin-1, an actin microfilament component. J Mol Biol. 2003;325:949–62.

518. Li R, Harvey AR, Hodgetts SI, Fox AH. Functional dissection of NEAT1 using genome editing reveals substantial localization of the NEAT1_1 isoform outside paraspeckles. RNA. 2017;23:872–81.

519. Li S, Koromilas AE. Dominant negative function by an alternatively spliced form of the interferon-inducible protein kinase PKR. J Biol Chem. 2001;276:13881–90.

520. Li T, Lu J, Zhong Y. Lentivirus-mediated shRNA interference targeting cyclooxygenase-2 inhibits growth of human non-small cell lung cancer. J BUON. 2013;18:908–14.

521. Li X, Wang J, Manley JL. Loss of splicing factor ASF/SF2 induces G2 cell cycle arrest and apoptosis, but inhibits internucleosomal DNA fragmentation. Genes Dev. 2005;19:2705–14.

522. Li Y, Hwang TH, Oseth LA, Hauge A, Vessella RL, Schmechel SC, et al. AR intragenic deletions linked to androgen receptor splice variant expression and activity in models of prostate cancer progression. Oncogene. 2012;31:4759–67.

523. Li Y, Zhang P, Choi T-Y, Park SK, Park H, Lee E-J, et al. Splicing-dependent trans-synaptic SALM3–LAR-RPTP interactions regulate excitatory synapse development and locomotion. Cell Reports. 2015;12:1618–30.

524. Li Y, Yu Z, Zhao X, Shen S-H. Identification and characterization of hepsin/-TM, a non-transmembrane hepsin isoform. Biochim Biophys Acta. 2005;1681:157–65.

525. Li Y, Xie N, Gleave ME, Rennie PS, Dong X. AR-v7 protein expression is regulated by protein kinase and phosphatase. Oncotarget. 2015;6:33743–54.

526. Li Y, Alsagabi M, Fan D, Bova GS, Tewfik AH, Dehm SM. Intragenic rearrangement and altered RNA splicing of the androgen receptor in a cell-based model of prostate cancer progression. Cancer Res. 2011;71:2108–17.

527. Li Z, Li Q, Han L, Tian N, Liang Q, Li Y, et al. Pro-apoptotic effects of splice-switching oligonucleotides targeting Bcl-x pre-mRNA in human glioma cell lines. Oncol Rep. 2016;35:1013–9.

528. Liang Y, Li C, Guzman VM, Chang WW, Evinger AJ, Sao D, et al. Identification of a novel alternative

splicing variant of RGS5 mRNA in human ocular tissues. FEBS J. 2005;272:791–9.

529. Liao P, Yu D, Li G, Yong TF, Soon JL, Chua YL, et al. A smooth muscle Cav1.2 calcium channel splice variant underlies hyperpolarized window current and enhanced state-dependent inhibition by nifedipine. J Biol Chem. 2007;282:35133–42.

530. Liao P, Yu D, Lu S, Tang Z, Liang MC, Zeng S, et al. Smooth muscle-selective alternatively spliced exon generates functional variation in Cav1.2 calcium channels. J Biol Chem. 2004;279:50329–35.

531. Lilly MB, Zemskova M, Frankel AE, Salo J, Kraft AS. Distinct domains of the human granulocyte-macrophage colony-stimulating factor receptor alpha subunit mediate activation of Jak/Stat signaling and differentiation. Blood. 2001;97:1662–70.

532. Lillycrop KA, Latchman DS. Alternative splicing of the Oct-2 transcription factor RNA is differentially regulated in neuronal cells and B cells and results in protein isoforms with opposite effects on the activity of octamer/TAATGARAT-containing promoters. J Biol Chem. 1992;267:24960–5.

533. Lin S-H, Cheng C-J, Lee Y-C, Ye X, Tsai W-W, Kim J, et al. A 45-kDa ErbB3 secreted by prostate cancer cells promotes bone formation. Oncogene. 2008;27:5195–203.

534. Lin Y, Stevens C, Harrison B, Pathuri S, Amin E, Hupp TR. The alternative splice variant of DAPK-1, s-DAPK-1, induces proteasome-independent DAPK-1 destabilization. Mol Cell Biochem. 2009;328:101–7.

535. Lin YS, Yasuda K, Assem M, Cline C, Barber J, Li C-W, et al. The major human pregnane X receptor (PXR) splice variant, PXR.2, exhibits significantly diminished ligand-activated transcriptional regulation. Drug Metab Dispos. 2009;37:1295–304.

536. Linares AJ, Lin C-H, Damianov A, Adams KL, Novitch BG, Black DL. The splicing regulator PTBP1 controls the activity of the transcription factor Pbx1 during neuronal differentiation. eLife. 2015;4:e09268.

537. Lindahl M, Timmusk T, Rossi J, Saarma M, Airaksinen MS. Expression and alternative splicing of mouse Gfra4 suggest roles in endocrine cell development. Mol Cell Neurosci. 2000;15:522–33.

538. Lindenboim L, Borner C, Stein R. Bcl-x(S) can form homodimers and heterodimers and its apoptotic activity requires localization of Bcl-x(S) to the mitochondria and its BH3 and loop domains. Cell Death Differ. 2001;8:933–42.

539. Lipkin SM, Chao EC, Moreno V, Rozek LS, Rennert H, Pinchev M, et al. Genetic variation in 3-hydroxy-3-methylglutaryl CoA reductase modifies the chemopreventive activity of statins for colorectal cancer. Cancer Prev Res (Phila). 2010;3:597–603.

540. Lira ME, Loomis AK, Paciga SA, Lloyd DB, Thompson JF. Expression of CETP and of splice variants induces the same level of ER stress despite secretion efficiency differences. J Lipid Res. 2008;49:1955–62.

541. Lishko VK, Yakubenko VP, Hertzberg KM, Grieninger G, Ugarova TP. The alternatively spliced alpha(E)C domain of human fibrinogen-420 is a novel ligand for leukocyte integrins alpha(M)beta(2) and alpha(X)beta(2). Blood. 2001;98:2448–55.

542. Liu HY, Wenzel-Seifert K, Seifert R. The olfactory G protein G(alphaolf) possesses a lower GDP-affinity and deactivates more rapidly than G(salphashort): consequences for receptor-coupling and adenylyl cyclase activation. J Neurochem. 2001;78:325–38.

543. Liu H, Wang K, Chen S, Sun Q, Zhang Y, Chen L, et al. NFATc1 phosphorylation by DYRK1A increases its protein stability. PLOS ONE. 2017;12:e0172985.

544. Liu J-W, Chandra D, Tang S-H, Chopra D, Tang DG. Identification and characterization of Bimgamma, a novel proapoptotic BH3-only splice variant of Bim. Cancer Res. 2002;62:2976–81.

545. Liu LL, Xie N, Sun S, Plymate S, Mostaghel E, Dong X. Mechanisms of the androgen receptor splicing in prostate cancer cells. Oncogene. 2014;33:3140–50.

546. Liu Q-R, Lu L, Zhu X-G, Gong J-P, Shaham Y, Uhl GR. Rodent BDNF genes, novel promoters, novel splice variants, and regulation by cocaine. Brain Res. 2006;1067:1–12.

547. Liu Q-R, Walther D, Drgon T, Polesskaya O, Lesnick TG, Strain KJ, et al. Human brain derived neurotrophic factor (BDNF) genes, splicing patterns, and assessments of associations with substance abuse and Parkinson's Disease. Am J Med Genet B Neuropsychiatr Genet. 2005;134B:93–103.

548. Liu X, Yang PS, Yang W, Yue DT. Enzyme-inhibitor-like tuning of Ca(2+) channel connectivity with calmodulin. Nature. 2010;463:968–72.

549. Liu Y, Samuel CE. Editing of glutamate receptor subunit B pre-mRNA by splice-site variants of interferon-inducible double-stranded RNA-specific adenosine deaminase ADAR1. J Biol Chem. 1999;274:5070–7.

550. Liu Y, Yang H, Li L, Chen S, Zuo F, Chen L. A novel VHLα isoform inhibits Warburg effect via modulation of PKM splicing. Tumour Biol. 2016;37:13649–57.

551. Liu Y, Bodmer WF. Analysis of P53 mutations and their expression in 56 colorectal cancer cell lines. Proc Natl Acad Sci USA. 2006;103:976–81.

552. Lladó A, Ezquerra M, Gaig C, Sánchez-Valle R, Tolosa E, Molinuevo JL. Brain tau expression and correlation with the H1/H1 tau genotype in frontotemporal lobar degeneration patients. J Neural Transm (Vienna). 2007;114:1585–8.

553. Llanos S, Cuadrado A, Serrano M. MSK2 inhibits p53 activity in the absence of stress. Sci Signal. 2009;2:ra57.

554. Llères D, Denegri M, Biggiogera M, Ajuh P, Lamond AI. Direct interaction between hnRNP-M and CDC5L/PLRG1 proteins affects alternative splice site choice. EMBO Rep. 2010;11:445–51.

555. Lo H-W, Zhu H, Cao X, Aldrich A, Ali-Osman F. A novel splice variant of GLI1 that promotes glioblastoma cell migration and invasion. Cancer Res. 2009;69:6790–8.

556. Lokeshwar VB, Estrella V, Lopez L, Kramer M, Gomez P, Soloway MS, et al. HYAL1-v1, an alternatively spliced variant of HYAL1 hyaluronidase: a negative regulator of bladder cancer. Cancer Res. 2006;66:11219–27.

557. Lonka-Nevalaita L, Lume M, Leppänen S, Jokitalo E, Peränen J, Saarma M. Characterization of the intracellular localization, processing, and secretion of two glial cell line-derived neurotrophic factor splice isoforms. J Neurosci. 2010;30:11403–13.

558. Lopez AY, Wang X, Xu M, Maheshwari A, Curry D, Lam S, et al. Ankyrin-G isoform imbalance and interneuronopathy link epilepsy and bipolar disorder. Mol Psychiatry. 2016. doi:10.1038/mp.2016.233.

559. Lopez V, Kelleher SL. Zinc transporter-2 (ZnT2) variants are localized to distinct subcellular compartments and functionally transport zinc. Biochem J. 2009;422:43–52.

560. Lopez-Mejia IC, De Toledo M, Della Seta F, Fafet P, Rebouissou C, Deleuze V, et al. Tissue-specific and SRSF1-dependent splicing of fibronectin, a matrix protein that controls host cell invasion. Mol Biol Cell. 2013;24:3164–76.

561. Lopez-Mejia IC, Toledo M de, Chavey C, Lapasset L, Cavelier P, Lopez-Herrera C, et al. Antagonistic functions of LMNA isoforms in energy expenditure and lifespan. EMBO reports. 2014;15:529–39.

562. Lorenz M, Hewing B, Hui J, Zepp A, Baumann G, Bindereif A, et al. Alternative splicing in intron 13 of the human eNOS gene: a potential mechanism for regulating eNOS activity. FASEB J. 2007;21:1556–64.

563. Lu H, Huang X, Zhang L, Guo Y, Cheng H, Zhou R. Multiple alternative splicing of mouse Dmrt1 during gonadal differentiation. Biochem Biophys Res Commun. 2007;352:630–4.

564. Lu J, Lonergan PE, Nacusi LP, Wang L, Schmidt LJ, Sun Z, et al. The cistrome and gene signature of androgen receptor splice variants in castration resistant prostate cancer cells. J Urol. 2015;193:690–8.

565. Lu JT, Son Y-J, Lee J, Jetton TL, Shiota M, Moscoso L, et al. Mice Lacking α-Calcitonin Gene-Related Peptide Exhibit Normal Cardiovascular Regulation and Neuromuscular Development. Molecular and Cellular Neuroscience. 1999;14:99–120.

566. Lu M, Conzen SD, Cole CN, Arrick BA. Characterization of functional messenger RNA splice variants of BRCA1 expressed in nonmalignant and tumor-derived breast cells. Cancer Res. 1996;56:4578–81.

567. Lu X, Ferreira PA. Identification of novel murine- and human-specific RPGRIP1 splice variants with distinct expression profiles and subcellular localization. Invest Ophthalmol Vis Sci. 2005;46:1882–90.

568. Lukas SM, Kroe RR, Wildeson J, Peet GW, Frego L, Davidson W, et al. Catalysis and function of the p38 alpha.MK2a signaling complex. Biochemistry. 2004;43:9950–60.

569. Lukashev D, Sitkovsky M. Preferential expression of the novel alternative isoform I.3 of hypoxia-inducible factor 1α in activated human T lymphocytes. Human Immunology. 2008;69:421–5.

570. Lüke Y, Zaim H, Karakesisoglou I, Jaeger VM, Sellin L, Lu W, et al. Nesprin-2 Giant (NUANCE) maintains nuclear envelope architecture and composition in skin. J Cell Sci. 2008;121:1887–98.

571. Lulli V, Romania P, Riccioni R, Boe A, Lo-Coco F, Testa U, et al. Transcriptional silencing of the ETS1 oncogene contributes to human granulocytic differentiation. Haematologica. 2010;95:1633–41.

572. Luo M-H, Tse S-W, Memmott J, Andreadis A. Novel isoforms of tau that lack the microtubule-binding domain. J Neurochem. 2004;90:340–51.

573. Luo Y, Yu H, Peterlin BM. Cellular protein modulates effects of human immunodeficiency virus type 1 Rev. J Virol. 1994;68:3850–6.

574. Lutz EM, Ronaldson E, Shaw P, Johnson MS, Holland PJ, Mitchell R. Characterization of novel splice variants of the PAC1 receptor in human neuroblastoma cells: consequences for signaling

by VIP and PACAP. Mol Cell Neurosci. 2006;31:193–209.

575. Lyman SD, James L, Escobar S, Downey H, de Vries P, Brasel K, et al. Identification of soluble and membrane-bound isoforms of the murine flt3 ligand generated by alternative splicing of mRNAs. Oncogene. 1995;10:149–57.

576. Lynch MJ, Baillie GS, Mohamed A, Li X, Maisonneuve C, Klussmann E, et al. RNA silencing identifies PDE4D5 as the functionally relevant cAMP phosphodiesterase interacting with beta arrestin to control the protein kinase A/AKAP79-mediated switching of the beta2-adrenergic receptor to activation of ERK in HEK293B2 cells. J Biol Chem. 2005;280:33178–89.

577. Ma C, Feng W, Han W, Lu Y, Liu W, Sui Y, et al. Elevated mRNA expression of PGF2α receptor splice variant 2(FP-V2) in human decidua is associated with incomplete mifepristone-misoprostol-induced early medical abortion by regulation of interleukin-8. J Matern Fetal Neonatal Med. 2016;29:3472–7.

578. Ma X, Kawamoto S, Uribe J, Adelstein RS. Function of the neuron-specific alternatively spliced isoforms of nonmuscle myosin II-B during mouse brain development. Mol Biol Cell. 2006;17:2138–49.

579. Macica CM, von Hehn CAA, Wang L-Y, Ho C-S, Yokoyama S, Joho RH, et al. Modulation of the kv3.1b potassium channel isoform adjusts the fidelity of the firing pattern of auditory neurons. J Neurosci. 2003;23:1133–41.

580. Maekawa S, Mori D, Nishiya T, Takikawa O, Horinouchi T, Nishimoto A, et al. OCTN2VT, a splice variant of OCTN2, does not transport carnitine because of the retention in the endoplasmic reticulum caused by insertion of 24 amino acids in the first extracellular loop of OCTN2. Biochim Biophys Acta. 2007;1773:1000–6.

581. Maglic D, Stovall DB, Cline JM, Fry EA, Mallakin A, Taneja P, et al. DMP1β, a splice isoform of the tumour suppressor DMP1 locus, induces proliferation and progression of breast cancer. J Pathol. 2015;236:90–102.

582. Mahajna J, Shi B, Bruskin A. A four-amino-acid insertion in the ligand-binding domain inactivates hRXRbeta and renders dominant negative activity. DNA Cell Biol. 1997;16:463–76.

583. Mahotka C, Liebmann J, Wenzel M, Suschek CV, Schmitt M, Gabbert HE, et al. Differential subcellular localization of functionally divergent survivin splice variants. Cell Death Differ. 2002;9:1334–42.

584. Maiweilidan Y, Klauza I, Kordeli E. Novel interactions of ankyrins-G at the costameres: the muscle-specific Obscurin/Titin-Binding-related Domain (OTBD) binds plectin and filamin C. Exp Cell Res. 2011;317:724–36.

585. Malentacchi F, Simi L, Nannelli C, Andreani M, Janni A, Pastorekova S, et al. Alternative splicing variants of carbonic anhydrase IX in human non-small cell lung cancer. Lung Cancer. 2009;64:271–6.

586. Mallory MJ, Jackson J, Weber B, Chi A, Heyd F, Lynch KW. Signal- and development-dependent alternative splicing of LEF1 in T cells is controlled by CELF2. Mol Cell Biol. 2011;31:2184–95.

587. Mango R, Biocca S, del Vecchio F, Clementi F, Sangiuolo F, Amati F, et al. In vivo and in vitro studies support that a new splicing isoform of OLR1 gene is protective against acute myocardial infarction. Circ Res. 2005;97:152–8.

588. Mangs AH, Speirs HJL, Goy C, Adams DJ, Markus MA, Morris BJ. XE7: a novel splicing factor that interacts with ASF/SF2 and ZNF265. Nucleic Acids Res. 2006;34:4976–86.

589. Mansergh FC, Hunter SM, Geatrell JC, Jarrin M, Powell K, Evans MJ, et al. Developmentally regulated expression of hemoglobin subunits in avascular tissues. Int J Dev Biol. 2004;52:873–86.

590. Marani M, Tenev T, Hancock D, Downward J, Lemoine NR. Identification of novel isoforms of the BH3 domain protein Bim which directly activate Bax to trigger apoptosis. Mol Cell Biol. 2002;22:3577–89.

591. Marcel V, Fernandes K, Terrier O, Lane DP, Bourdon J-C. Modulation of p53β and p53γ expression by regulating the alternative splicing of TP53 gene modifies cellular response. Cell Death Differ. 2014;21:1377–87.

592. Marcel V, Tran PLT, Sagne C, Martel-Planche G, Vaslin L, Teulade-Fichou M-P, et al. G-quadruplex structures in TP53 intron 3: role in alternative splicing and in production of p53 mRNA isoforms. Carcinogenesis. 2011;32:271–8.

593. Marcias G, Erdmann E, Lapouge G, Siebert C, Barthélémy P, Duclos B, et al. Identification of novel truncated androgen receptor (AR) mutants including unreported pre-mRNA splicing variants in the 22Rv1 hormone-refractory prostate cancer (PCa) cell line. Hum Mutat. 2010;31:74–80.

594. Markus MA, Heinrich B, Raitskin O, Adams DJ, Mangs H, Goy C, et al. WT1 interacts with the splicing protein RBM4 and regulates its ability to modulate alternative splicing in vivo. Exp Cell Res. 2006;312:3379–88.

595. Martin D, Li Y, Yang J, Wang G, Margariti A, Jiang Z, et al. Unspliced X-box-binding Protein 1 (XBP1) Protects Endothelial Cells from Oxidative Stress through Interaction with Histone Deacetylase 3. J Biol Chem. 2014;289:30625–34.

596. Martin KR, Xu Y, Looyenga BD, Davis RJ, Wu C-L, Tremblay ML, et al. Identification of PTPσ as an autophagic phosphatase. J Cell Sci. 2011;124:812–9.

597. Martin MM, Willardson BM, Burton GF, White CR, McLaughlin JN, Bray SM, et al. Human angiotensin II type 1 receptor isoforms encoded by messenger RNA splice variants are functionally distinct. Mol Endocrinol. 2001;15:281–93.

598. Martinez O, Brackenridge S, El-Idrissi ME-A, Prabhakar BS. DC-SIGN, but not sDC-SIGN, can modulate IL-2 production from PMA- and anti-CD3-stimulated primary human CD4 T cells. Int Immunol. 2005;17:769–78.

599. Martino MEB, Olsen JC, Fulcher NB, Wolfgang MC, O’Neal WK, Ribeiro CMP. Airway epithelial inflammation-induced endoplasmic reticulum Ca2+ store expansion is mediated by X-box binding protein-1. J Biol Chem. 2009;284:14904–13.

600. Massiello A, Chalfant CE. SRp30a (ASF/SF2) regulates the alternative splicing of caspase-9 pre-mRNA and is required for ceramide-responsiveness. J Lipid Res. 2006;47:892–7.

601. Master A, Wójcicka A, Piekiełko-Witkowska A, Bogusławska J, Popławski P, Tański Z, et al. Untranslated regions of thyroid hormone receptor beta 1 mRNA are impaired in human clear cell renal cell carcinoma. Biochim Biophys Acta. 2010;1802:995–1005.

602. Matic M, Corradin AP, Tsoli M, Clarke SJ, Polly P, Robertson GR. The alternatively spliced murine pregnane X receptor isoform, mPXR(delta171-211) exhibits a repressive action. Int J Biochem

Cell Biol. 2010;42:672–82.

603. Matsushita K, Takeoka M, Sagara J, Itano N, Kurose Y, Nakamura A, et al. A splice variant of ASC regulates IL-1beta release and aggregates differently from intact ASC. Mediators Inflamm. 2009;2009:287387.

604. Matsushita K, Kajiwara T, Tamura M, Satoh M, Tanaka N, Tomonaga T, et al. SAP155-mediated splicing of FUSE-binding protein-interacting repressor serves as a molecular switch for c-myc gene expression. Mol Cancer Res. 2012;10:787–99.

605. Matsushita K, Tamura M, Tanaka N, Tomonaga T, Matsubara H, Shimada H, et al. Interactions between SAP155 and FUSE-binding protein-interacting repressor bridges c-Myc and P27Kip1 expression. Mol Cancer Res. 2013;11:689–98.

606. Matsushita K, Tomonaga T, Shimada H, Shioya A, Higashi M, Matsubara H, et al. An essential role of alternative splicing of c-myc suppressor FUSE-binding protein-interacting repressor in carcinogenesis. Cancer Res. 2006;66:1409–17.

607. Matsushita M, Yamamoto R, Mitsui K, Kanazawa H. Altered motor activity of alternative splice variants of the mammalian kinesin-3 protein KIF1B. Traffic. 2009;10:1647–54.

608. Mavrou A, Brakspear K, Hamdollah-Zadeh M, Damodaran G, Babaei-Jadidi R, Oxley J, et al. Serine-arginine protein kinase 1 (SRPK1) inhibition as a potential novel targeted therapeutic strategy in prostate cancer. Oncogene. 2015;34:4311–9.

609. Mayerhofer PU, Kattenfeld T, Roscher AA, Muntau AC. Two splice variants of human PEX19 exhibit distinct functions in peroxisomal assembly. Biochem Biophys Res Commun. 2002;291:1180–6.

610. Maynard KR, Hill JL, Calcaterra NE, Palko ME, Kardian A, Paredes D, et al. Functional Role of BDNF Production from Unique Promoters in Aggression and Serotonin Signaling. Neuropsychopharmacology. 2016;41:1943–55.

611. Mayr JA, Merkel O, Kohlwein SD, Gebhardt BR, Böhles H, Fötschl U, et al. Mitochondrial phosphate-carrier deficiency: a novel disorder of oxidative phosphorylation. Am J Hum Genet. 2007;80:478–84.

612. Mayshar Y, Rom E, Chumakov I, Kronman A, Yayon A, Benvenisty N. Fibroblast growth factor 4 and its novel splice isoform have opposing effects on the maintenance of human embryonic stem cell self-renewal. Stem Cells. 2008;26:767–74.

613. McCartney CE, McClafferty H, Huibant J-M, Rowan EG, Shipston MJ, Rowe ICM. A cysteine-rich motif confers hypoxia sensitivity to mammalian large conductance voltage- and Ca-activated K (BK) channel alpha-subunits. Proc Natl Acad Sci USA. 2005;102:17870–6.

614. McElvaine AT, Mayo KE. A dominant-negative human growth hormone-releasing hormone (GHRH) receptor splice variant inhibits GHRH binding. Endocrinology. 2006;147:1884–94.

615. McMahon AC, Barnett MW, O'Leary TS, Stoney PN, Collins MO, Papadia S, et al. SynGAP isoforms exert opposing effects on synaptic strength. Nat Commun. 2012;3:900.

616. McMillan P, Korvatska E, Poorkaj P, Evstafjeva Z, Robinson L, Greenup L, et al. Tau isoform regulation is region- and cell-specific in mouse brain. J Comp Neurol. 2008;511:788–803.

617. Mei H, Wang Y, Fan J, Lin Z. Alternative splicing of S6K1 promotes non-small cell lung cancer survival. Tumour Biol. 2016;37:13369–76.

618. Mei Y, Xie C, Xie W, Wu Z, Wu M. Siah-1S, a novel splice variant of Siah-1 (seven in absentia homolog), counteracts Siah-1-mediated downregulation of beta-catenin. Oncogene. 2007;26:6319–31.

619. Meidan R, Klipper E, Gilboa T, Muller L, Levy N. Endothelin-converting enzyme-1, abundance of isoforms a-d and identification of a novel alternatively spliced variant lacking a transmembrane domain. J Biol Chem. 2005;280:40867–74.

620. Meijer OC, Kalkhoven E, van der Laan S, Steenbergen PJ, Houtman SH, Dijkmans TF, et al. Steroid receptor coactivator-1 splice variants differentially affect corticosteroid receptor signaling. Endocrinology. 2005;146:1438–48.

621. Meiners S, Nur-e-Kamal MS, Mercado ML. Identification of a neurite outgrowth-promoting motif within the alternatively spliced region of human tenascin-C. J Neurosci. 2001;21:7215–25.

622. Meng H, Tian L, Zhou J, Li Z, Jiao X, Li WW, et al. PACSIN 2 represses cellular migration through direct association with cyclin D1 but not its alternate splice form cyclin D1b. Cell Cycle. 2011;10:73–81.

623. Menn B, Timsit S, Calothy G, Lamballe F. Differential expression of TrkC catalytic and noncatalytic isoforms suggests that they act independently or in association. J Comp Neurol. 1998;401:47–64.

624. Mercatante DR, Bortner CD, Cidlowski JA, Kole R. Modification of alternative splicing of Bcl-x pre-mRNA in prostate and breast cancer cells. analysis of apoptosis and cell death. J Biol Chem. 2001;276:16411–7.

625. Mercatante DR, Mohler JL, Kole R. Cellular response to an antisense-mediated shift of Bcl-x pre-mRNA splicing and antineoplastic agents. J Biol Chem. 2002;277:49374–82.

626. Meshorer E, Toiber D, Zurel D, Sahly I, Dori A, Cagnano E, et al. Combinatorial complexity of 5' alternative acetylcholinesterase transcripts and protein products. J Biol Chem. 2004;279:29740–51.

627. Metz RP, Kwak H-I, Gustafson T, Laffin B, Porter WW. Differential transcriptional regulation by mouse single-minded 2s. J Biol Chem. 2006;281:10839–48.

628. Micheal S, Ayub H, Islam F, Siddiqui SN, Khan WA, Akhtar F, et al. Variants in the ASB10 Gene Are Associated with Primary Open Angle Glaucoma. PLoS ONE. 2015;10:e0145005.

629. Miederer A-M, Alansary D, Schwär G, Lee P-H, Jung M, Helms V, et al. A STIM2 splice variant negatively regulates store-operated calcium entry. Nat Commun. 2015;6:6899.

630. Miescher GC, Lützelschwab R, Erne B, Ferracin F, Huber S, Steck AJ. Reciprocal expression of myelin-associated glycoprotein splice variants in the adult human peripheral and central nervous systems. Brain Res Mol Brain Res. 1997;52:299–306.

631. Migliaccio E, Mele S, Salcini AE, Pelicci G, Lai K-MV, Superti-Furga G, et al. Opposite effects of the p52shc/p46shc and p66shc splicing isoforms on the EGF receptor–MAP kinase–fos signalling pathway. The EMBO Journal. 1997;16:706–16.

632. Miki T, Bottaro DP, Fleming TP, Smith CL, Burgess WH, Chan AM, et al. Determination of ligand-binding specificity by alternative splicing: two distinct growth factor receptors encoded by a single gene. Proc Natl Acad Sci USA. 1992;89:246–50.

633. Millet C, Lemaire P, Orsetti B, Guglielmi P, François V. The human chordin gene encodes several

differentially expressed spliced variants with distinct BMP opposing activities. Mech Dev. 2001;106:85–96.

634. Minegishi T, Nakamura K, Yamashita S, Omori Y. The effect of splice variant of the human luteinizing hormone (LH) receptor on the expression of gonadotropin receptor. Mol Cell Endocrinol. 2007;260–262:117–25.

635. Minn AJ, Boise LH, Thompson CB. Bcl-x(S) anatagonizes the protective effects of Bcl-x(L). J Biol Chem. 1996;271:6306–12.

636. Minvielle S, Giscard-Dartevelle S, Cohen R, Taboulet J, Labye F, Jullienne A, et al. A novel calcitonin carboxyl-terminal peptide produced in medullary thyroid carcinoma by alternative RNA processing of the calcitonin/calcitonin gene-related peptide gene. J Biol Chem. 1991;266:24627–31.

637. Mion S, Corti C, Neki A, Shigemoto R, Corsi M, Fumagalli G, et al. Bidirectional regulation of neurite elaboration by alternatively spliced metabotropic glutamate receptor 5 (mGluR5) isoforms. Mol Cell Neurosci. 2001;17:957–72.

638. Mitani Y, Li J, Weber RS, Lippman SL, Flores ER, Caulin C, et al. Expression and regulation of the ΔN and TAp63 isoforms in salivary gland tumorigenesis clinical and experimental findings. Am J Pathol. 2011;179:391–9.

639. Mitra RS, Benedict MA, Qian D, Foreman KE, Ekhterae D, Nickoloff BJ, et al. Killing of sarcoma cells by proapoptotic Bcl-X(S): role of the BH3 domain and regulation by Bcl-X(L). Neoplasia. 2001;3:437–45.

640. Miyake Y, Mizuno T, Yanagi K, Hanaoka F. Novel splicing variant of mouse Orc1 is deficient in nuclear translocation and resistant for proteasome-mediated degradation. J Biol Chem. 2005;280:12643–52.

641. Miyatake R, Furukawa A, Matsushita M, Iwahashi K, Nakamura K, Ichikawa Y, et al. Tissue-specific alternative splicing of mouse brain type ryanodine receptor/calcium release channel mRNA. FEBS Lett. 1996;395:123–6.

642. Miyazawa K, Williams DA, Gotoh A, Nishimaki J, Broxmeyer HE, Toyama K. Membrane-bound Steel factor induces more persistent tyrosine kinase activation and longer life span of c-kit gene-encoded protein than its soluble form. Blood. 1995;85:641–9.

643. Mizoguchi H, Watanabe C, Higashiya T, Takeda S, Moriyama K, Aoki Y, et al. Distinct physiological role of amidino-TAPA-sensitive and DAMGO-insensitive μ-opioid receptor splice variants in the mouse spinal cord. Eur J Pharmacol. 2013;711:80–6.

644. Mizuno K, Hasegawa K, Ogimoto M, Katagiri T, Yakura H. Developmental regulation of gene expression for the MPTPδ isoforms in the central nervous system and the immune system. FEBS Letters. 1994;355:223–8.

645. Molina E, Hermida J, López-Sagaseta J, Puy C, Montes R. The functional properties of a truncated form of endothelial cell protein C receptor generated by alternative splicing. Haematologica. 2008;93:878–84.

646. Monfregola J, Cevenini A, Terracciano A, van Vlies N, Arbucci S, Wanders RJA, et al. Functional analysis of TMLH variants and definition of domains required for catalytic activity and mitochondrial targeting. J Cell Physiol. 2005;204:839–47.

647. Montague P, McCallion AS, Barrie JE, Edgar JM, McLaughlin M, Davies RW, et al. Characterization of the murine splice variant Mobp155: developmental CNS expression pattern and subcellular localization of epitope-tagged protein. Glia. 2005;50:80–5.

648. Montalbano J, Jin W, Sheikh MS, Huang Y. RBEL1 is a novel gene that encodes a nucleocytoplasmic Ras superfamily GTP-binding protein and is overexpressed in breast cancer. J Biol Chem. 2007;282:37640–9.

649. Montalbano J, Lui K, Sheikh MS, Huang Y. Identification and characterization of RBEL1 subfamily of GTPases in the Ras superfamily involved in cell growth regulation. J Biol Chem. 2009;284:18129–42.

650. Monterrat C, Boal F, Grise F, Hémar A, Lang J. Synaptotagmin 8 is expressed both as a calcium-insensitive soluble and membrane protein in neurons, neuroendocrine and endocrine cells. Biochim Biophys Acta. 2006;1763:73–81.

651. Montes M, Cloutier A, Sánchez-Hernández N, Michelle L, Lemieux B, Blanchette M, et al. TCERG1 regulates alternative splicing of the Bcl-x gene by modulating the rate of RNA polymerase II transcription. Mol Cell Biol. 2012;32:751–62.

652. Moon H, Cho S, Loh TJ, Oh HK, Jang HN, Zhou J, et al. SRSF2 promotes splicing and transcription of exon 11 included isoform in Ron proto-oncogene. Biochim Biophys Acta. 2014;1839:1132–40.

653. Morales AA, Olsson A, Celsing F, Osterborg A, Jondal M, Osorio LM. Expression and transcriptional regulation of functionally distinct Bmf isoforms in B-chronic lymphocytic leukemia cells. Leukemia. 2004;18:41–7.

654. Morihara T, Hayashi N, Yokokoji M, Akatsu H, Silverman MA, Kimura N, et al. Transcriptome analysis of distinct mouse strains reveals kinesin light chain-1 splicing as an amyloid-β accumulation modifier. PNAS. 2014;111:2638–43.

655. Morten BC, Wong-Brown MW, Scott RJ, Avery-Kiejda KA. The presence of the intron 3 16 bp duplication polymorphism of p53 (rs17878362) in breast cancer is associated with a low Δ40p53:p53 ratio and better outcome. Carcinogenesis. 2016;37:81–6.

656. Mosedale M, Egodage S, Calma RC, Chi N-W, Chessler SD. Neurexin-1α Contributes to Insulin-containing Secretory Granule Docking. J Biol Chem. 2012;287:6350–61.

657. Mosinger B, Tillmann U, Westphal H, Tremblay ML. Cloning and characterization of a mouse cDNA encoding a cytoplasmic protein-tyrosine-phosphatase. Proc Natl Acad Sci U S A. 1992;89:499–503.

658. Mosley JD, Keri RA. Splice variants of mIAP1 have an enhanced ability to inhibit apoptosis. Biochem Biophys Res Commun. 2006;348:1174–83.

659. Mosselman S, Claesson-Welsh L, Kamphuis JS, van Zoelen EJ. Developmentally regulated expression of two novel platelet-derived growth factor alpha-receptor transcripts in human teratocarcinoma cells. Cancer Res. 1994;54:220–5.

660. Mostafavi-Pour Z, Askari JA, Whittard JD, Humphries MJ. Identification of a novel heparin-binding site in the alternatively spliced IIICS region of fibronectin: roles of integrins and proteoglycans in cell adhesion to fibronectin splice variants. Matrix Biol. 2001;20:63–73.

661. Motoyama M, Yamazaki S, Eto-Kimura A, Takeshige K, Muta T. Positive and negative regulation of nuclear factor-kappaB-mediated transcription by IkappaB-zeta, an inducible nuclear protein. J

Biol Chem. 2005;280:7444–51.

662. Motoyama N, Wang F, Roth KA, Sawa H, Nakayama K, Nakayama K, et al. Massive cell death of immature hematopoietic cells and neurons in Bcl-x-deficient mice. Science. 1995;267:1506–10.

663. Mou H, Smith JL, Peng L, Yin H, Moore J, Zhang X-O, et al. CRISPR/Cas9-mediated genome editing induces exon skipping by alternative splicing or exon deletion. Genome Biology. 2017;18:108.

664. Moulton VR, Gillooly AR, Perl MA, Markopoulou A, Tsokos GC. Serine Arginine-Rich Splicing Factor 1 (SRSF1) Contributes to the Transcriptional Activation of CD3ζ in Human T Cells. PLoS One. 2015;10. doi:10.1371/journal.pone.0131073.

665. Moulton VR, Kyttaris VC, Juang Y-T, Chowdhury B, Tsokos GC. The RNA-stabilizing protein HuR regulates the expression of zeta chain of the human T cell receptor-associated CD3 complex. J Biol Chem. 2008;283:20037–44.

666. Moulton VR, Tsokos GC. Alternative splicing factor/splicing factor 2 regulates the expression of the zeta subunit of the human T cell receptor-associated CD3 complex. J Biol Chem. 2010;285:12490–6.

667. Mouquet H, Farci S, Joly P, Maillère B, Leblond J, Drouot L, et al. A truncated alternative spliced isoform of human desmoglein 1 contains a specific T cell epitope binding to the pemphigus foliaceus-associated HLA class II DRbeta1*0102 molecule. J Immunol. 2006;177:6517–26.

668. Mourelatos Z, Abel L, Yong J, Kataoka N, Dreyfuss G. SMN interacts with a novel family of hnRNP and spliceosomal proteins. EMBO J. 2001;20:5443–52.

669. Muda M, He C, Martini PGV, Ferraro T, Layfield S, Taylor D, et al. Splice variants of the relaxin and INSL3 receptors reveal unanticipated molecular complexity. Mol Hum Reprod. 2005;11:591–600.

670. Muise AM, Walters T, Wine E, Griffiths AM, Turner D, Duerr RH, et al. Protein-Tyrosine Phosphatase Sigma Is Associated with Ulcerative Colitis. Current Biology. 2007;17:1212–8.

671. Mukhopadhyay SS, Wyszomierski SL, Gronostajski RM, Rosen JM. Differential interactions of specific nuclear factor I isoforms with the glucocorticoid receptor and STAT5 in the cooperative regulation of WAP gene transcription. Mol Cell Biol. 2001;21:6859–69.

672. Mulcahy H, O'Rourke KP, Adams C, Molloy MG, O'Gara F. LST1 and NCR3 expression in autoimmune inflammation and in response to IFN-gamma, LPS and microbial infection. Immunogenetics. 2006;57:893–903.

673. Mulherkar N, Ramaswamy M, Mordi DC, Prabhakar BS. MADD/DENN splice variant of the IG20 gene is necessary and sufficient for cancer cell survival. Oncogene. 2006;25:6252–61.

674. Mulherkar N, Prasad KV, Prabhakar BS. MADD/DENN splice variant of the IG20 gene is a negative regulator of caspase-8 activation. Knockdown enhances TRAIL-induced apoptosis of cancer cells. J Biol Chem. 2007;282:11715–21.

675. Munkley J, Oltean S, Vodák D, Wilson BT, Livermore KE, Zhou Y, et al. The androgen receptor controls expression of the cancer-associated sTn antigen and cell adhesion through induction of ST6GalNAc1 in prostate cancer. Oncotarget. 2015;6:34358–74.

676. Murakami T, Sakane F, Imai S, Houkin K, Kanoh H. Identification and Characterization of Two Splice Variants of Human Diacylglycerol Kinase η. J Biol Chem. 2003;278:34364–72.

677. Muraoka-Cook RS, Sandahl MA, Strunk KE, Miraglia LC, Husted C, Hunter DM, et al. ErbB4 splice variants Cyt1 and Cyt2 differ by 16 amino acids and exert opposing effects on the mammary epithelium in vivo. Mol Cell Biol. 2009;29:4935–48.

678. Nagy G, Milosevic I, Fasshauer D, Müller EM, Groot BL de, Lang T, et al. Alternative splicing of SNAP-25 regulates secretion through nonconservative substitutions in the SNARE domain. Mol Biol Cell. 2005;16:5675–85.

679. Nakabeppu Y, Nathans D. A naturally occurring truncated form of FosB that inhibits Fos/Jun transcriptional activity. Cell. 1991;64:751–9.

680. Nakajima M, Miyamoto Y, Ikegawa S. Cloning and characterization of the osteoarthritis-associated gene DVWA. J Bone Miner Metab. 2011;29:300–8.

681. Nakamura Y, Komatsu N, Nakauchi H. A truncated erythropoietin receptor that fails to prevent programmed cell death of erythroid cells. Science. 1992;257:1138–41.

682. Nakano J, Huang C, Liu D, Masuya D, Yokomise H, Ueno M, et al. The clinical significance of splice variants and subcellular localisation of survivin in non-small cell lung cancers. Br J Cancer. 2008;98:1109–17.

683. Nakano K, Vousden KH. PUMA, a novel proapoptotic gene, is induced by p53. Mol Cell. 2001;7:683–94.

684. Nakata D, Nakao S, Nakayama K, Araki S, Nakayama Y, Aparicio S, et al. The RNA helicase DDX39B and its paralog DDX39A regulate androgen receptor splice variant AR-V7 generation. Biochem Biophys Res Commun. 2017;483:271–6.

685. Nakata T, Yokota T, Emi M, Minami S. Differential expression of multiple isoforms of the ELKS mRNAs involved in a papillary thyroid carcinoma. Genes, Chromosomes and Cancer. 2002;35:30–7.

686. Nakayama K, Nakayama K, Negishi I, Kuida K, Sawa H, Loh DY. Targeted disruption of Bcl-2 alpha beta in mice: occurrence of gray hair, polycystic kidney disease, and lymphocytopenia. Proc Natl Acad Sci USA. 1994;91:3700–4.

687. Narla G, DiFeo A, Yao S, Banno A, Hod E, Reeves HL, et al. Targeted inhibition of the KLF6 splice variant, KLF6 SV1, suppresses prostate cancer cell growth and spread. Cancer Res. 2005;65:5761–8.

688. Naughton BJ, Thirtamara-Rajamani K, Wang C, During MJ, Gu HH. Specific knockdown of the D2 long dopamine receptor variant. Neuroreport. 2012;23:1–5.

689. Naumann T, Casademunt E, Hollerbach E, Hofmann J, Dechant G, Frotscher M, et al. Complete deletion of the neurotrophin receptor p75NTR leads to long-lasting increases in the number of basal forebrain cholinergic neurons. J Neurosci. 2002;22:2409–18.

690. Nestler EJ, Kelz MB, Chen J. DeltaFosB: a molecular mediator of long-term neural and behavioral plasticity. Brain Res. 1999;835:10–7.

691. Niccoli-Sire P, Fayadat L, Siffroi-Fernandez S, Malthierry Y, Franc JL. Alternatively spliced form of human thyroperoxidase, TPOzanelli: activity, intracellular trafficking, and role in hormonogenesis. Biochemistry. 2001;40:2572–9.

692. Nicholls CD, Shields MA, Lee PWK, Robbins SM, Beattie TL. UV-dependent alternative splicing

uncouples p53 activity and PIG3 gene function through rapid proteolytic degradation. J Biol Chem. 2004;279:24171–8.

693. Nicotera TM, Schuster DP, Bourhim M, Chadha K, Klaich G, Corral DA. Regulation of PSA secretion and survival signaling by calcium-independent phopholipase A(2)beta in prostate cancer cells. Prostate. 2009;69:1270–80.

694. Nie D-S, Liu Y-B, Lu G-X. Cloning and primarily function study of two novel putative N5-glutamine methyltransferase (Hemk) splice variants from mouse stem cells. Mol Biol Rep. 2009;36:2221–8.

695. Nishimura H, Fujimoto A, Tamura N, Yajima T, Wajjwalku W, Yoshikai Y. A novel autoregulatory mechanism for transcriptional activation of the IL-15 gene by a nonsecretable isoform of IL-15 generated by alternative splicing. FASEB J. 2005;19:19–28.

696. Nishioka Y, Imaizumi H, Imada J, Katahira J, Matsuura N, Hieda M. SUN1 splice variants, SUN1_888, SUN1_785, and predominant SUN1_916, variably function in directional cell migration. Nucleus. 2016;7:572–84.

697. Nishizawa Y, Usukura J, Singh DP, Chylack LT, Shinohara T. Spatial and temporal dynamics of two alternatively spliced regulatory factors, lens epithelium-derived growth factor (ledgf/p75) and p52, in the nucleus. Cell Tissue Res. 2001;305:107–14.

698. Nitz MD, Harding MA, Smith SC, Thomas S, Theodorescu D. RREB1 transcription factor splice variants in urologic cancer. Am J Pathol. 2011;179:477–86.

699. Nogueira TC, Paula FM, Villate O, Colli ML, Moura RF, Cunha DA, et al. GLIS3, a susceptibility gene for type 1 and type 2 diabetes, modulates pancreatic beta cell apoptosis via regulation of a splice variant of the BH3-only protein Bim. PLoS Genet. 2013;9:e1003532.

700. Noutsios GT, Silveyra P, Bhatti F, Floros J. Exon B of human surfactant protein A2 mRNA, alone or within its surrounding sequences, interacts with 14-3-3; role of cis-elements and secondary structure. Am J Physiol Lung Cell Mol Physiol. 2013;304:L722–35.

701. Novikov L, Park JW, Chen H, Klerman H, Jalloh AS, Gamble MJ. QKI-mediated alternative splicing of the histone variant MacroH2A1 regulates cancer cell proliferation. Mol Cell Biol. 2011;31:4244–55.

702. Nowak DG, Woolard J, Amin EM, Konopatskaya O, Saleem MA, Churchill AJ, et al. Expression of pro- and anti-angiogenic isoforms of VEGF is differentially regulated by splicing and growth factors. Journal of Cell Science. 2008;121:3487–95.

703. Nurmi JT, Puolakkainen PA, Rautonen NE. Intron 1 retaining cyclooxygenase 1 splice variant is induced by osmotic stress in human intestinal epithelial cells. Prostaglandins Leukot Essent Fatty Acids. 2005;73:343–50.

704. Nutthasirikul N, Limpaiboon T, Leelayuwat C, Patrakitkomjorn S, Jearanaikoon P. Ratio disruption of the Δ133p53 and TAp53 isoform equilibrium correlates with poor clinical outcome in intrahepatic cholangiocarcinoma. Int J Oncol. 2013;42:1181–8.

705. Nyman U, Sobczak-Pluta A, Vlachos P, Perlmann T, Zhivotovsky B, Joseph B. Full-length p73alpha represses drug-induced apoptosis in small cell lung carcinoma cells. J Biol Chem. 2005;280:34159–69.

706. O'Connor L, Strasser A, O'Reilly LA, Hausmann G, Adams JM, Cory S, et al. Bim: a novel member of the Bcl-2 family that promotes apoptosis. EMBO J. 1998;17:384–95.

707. O'Grady P, Thai TC, Saito H. The Laminin–Nidogen Complex is a Ligand for a Specific Splice Isoform of the Transmembrane Protein Tyrosine Phosphatase LAR. The Journal of Cell Biology. 1998;141:1675–84.

708. O'Leary H, Sui X, Lin P-J, Volpe P, Bayer KU. Nuclear targeting of the CaMKII anchoring protein alphaKAP is regulated by alternative splicing and protein kinases. Brain Res. 2006;1086:17–26.

709. O'Malley KL, Harmon S, Moffat M, Uhland-Smith A, Wong S. The human aromatic L-amino acid decarboxylase gene can be alternatively spliced to generate unique protein isoforms. J Neurochem. 1995;65:2409–16.

710. O'Rourke JP, Ness SA. Alternative RNA splicing produces multiple forms of c-Myb with unique transcriptional activities. Mol Cell Biol. 2008;28:2091–101.

711. Offenhäuser N, Muzio V, Biffo S. BDNF binding to truncated trkB.T1 does not affect gene expression. Neuroreport. 2002;13:1189–93.

712. Oh J-H, Yang JO, Hahn Y, Kim M-R, Byun S-S, Jeon Y-J, et al. Transcriptome analysis of human gastric cancer. Mamm Genome. 2005;16:942–54.

713. Oh SW, Pope RK, Smith KP, Crowley JL, Nebl T, Lawrence JB, et al. Archvillin, a muscle-specific isoform of supervillin, is an early expressed component of the costameric membrane skeleton. Journal of Cell Science. 2003;116:2261–75.

714. Ohsaki Y, Kawai T, Yoshikawa Y, Cheng J, Jokitalo E, Fujimoto T. PML isoform II plays a critical role in nuclear lipid droplet formation. J Cell Biol. 2016;212:29–38.

715. Okabe S, Miwa A, Okado H. Alternative splicing of the C-terminal domain regulates cell surface expression of the NMDA receptor NR1 subunit. J Neurosci. 1999;19:7781–92.

716. Olsen SK, Li JYH, Bromleigh C, Eliseenkova AV, Ibrahimi OA, Lao Z, et al. Structural basis by which alternative splicing modulates the organizer activity of FGF8 in the brain. Genes Dev. 2006;20:185–98.

717. Olson PA, Tkatch T, Hernandez-Lopez S, Ulrich S, Ilijic E, Mugnaini E, et al. G-protein-coupled receptor modulation of striatal CaV1.3 L-type Ca2+ channels is dependent on a Shank-binding domain. J Neurosci. 2005;25:1050–62.

718. Oltvai ZN, Milliman CL, Korsmeyer SJ. Bcl-2 heterodimerizes in vivo with a conserved homolog, Bax, that accelerates programmed cell death. Cell. 1993;74:609–19.

719. Oppermann M, Mizel D, Huang G, Li C, Deng C, Theilig F, et al. Macula densa control of renin secretion and preglomerular resistance in mice with selective deletion of the B isoform of the Na,K,2Cl co-transporter. J Am Soc Nephrol. 2006;17:2143–52.

720. Orban TI, Olah E. Expression profiles of BRCA1 splice variants in asynchronous and in G1/S synchronized tumor cell lines. Biochem Biophys Res Commun. 2001;280:32–8.

721. Orlichenko L, Geyer R, Yanagisawa M, Khauv D, Radisky ES, Anastasiadis PZ, et al. The 19-amino acid insertion in the tumor-associated splice isoform Rac1b confers specific binding to p120 catenin. J Biol Chem. 2010;285:19153–61.

722. Ortuño-Pineda C, Galindo-Rosales JM, Calderón-Salinas JV, Villegas-Sepúlveda N, Saucedo-Cárdenas O, De Nova-Ocampo M, et al. Binding of hnRNP H and U2AF65 to respective G-codes and a poly-uridine tract collaborate in the N50-5'ss selection of the REST N exon in H69 cells.

PLoS ONE. 2012;7:e40315.

723. Osuka F, Endo Y, Higuchi M, Suzuki H, Shio Y, Fujiu K, et al. Molecular cloning and characterization of novel splicing variants of human decay-accelerating factor. Genomics. 2006;88:316–22.

724. Oudejans CB, Poutsma A, Michel OJ, Thulluru HK, Mulders J, van de Vrugt HJ, et al. Noncoding RNA-regulated gain-of-function of STOX2 in Finnish pre-eclamptic families. Sci Rep. 2016;6:32129.

725. Ovando-Roche P, Yu JSL, Testori S, Ho C, Cui W. TRF2-mediated stabilization of hREST4 is critical for the differentiation and maintenance of neural progenitors. Stem Cells. 2014;32:2111–22.

726. Pachlopnik Schmid J, Lemoine R, Nehme N, Cormier-Daire V, Revy P, Debeurme F, et al. Polymerase ε1 mutation in a human syndrome with facial dysmorphism, immunodeficiency, livedo, and short stature ("FILS syndrome"). J Exp Med. 2012;209:2323–30.

727. Palsson-McDermott EM, Doyle SL, McGettrick AF, Hardy M, Husebye H, Banahan K, et al. TAG, a splice variant of the adaptor TRAM, negatively regulates the adaptor MyD88-independent TLR4 pathway. Nat Immunol. 2009;10:579–86.

728. Palve VC, Teni TR. Association of anti-apoptotic Mcl-1L isoform expression with radioresistance of oral squamous carcinoma cells. Radiat Oncol. 2012;7:135.

729. Palve V, Mallick S, Ghaisas G, Kannan S, Teni T. Overexpression of Mcl-1L splice variant is associated with poor prognosis and chemoresistance in oral cancers. PLoS ONE. 2014;9:e111927.

730. Paolella G, Henchcliffe C, Sebastio G, Baralle FE. Sequence analysis and in vivo expression show that alternative splicing of ED-B and ED-A regions of the human fibronectin gene are independent events. Nucleic Acids Res. 1988;16:3545–57.

731. Papadopoulos C, Arato K, Lilienthal E, Zerweck J, Schutkowski M, Chatain N, et al. Splice variants of the dual specificity tyrosine phosphorylation-regulated kinase 4 (DYRK4) differ in their subcellular localization and catalytic activity. J Biol Chem. 2011;286:5494–505.

732. Parent JL, Labrecque P, Driss Rochdi M, Benovic JL. Role of the differentially spliced carboxyl terminus in thromboxane A2 receptor trafficking: identification of a distinct motif for tonic internalization. J Biol Chem. 2001;276:7079–85.

733. Park JE, Keller GA, Ferrara N. The vascular endothelial growth factor (VEGF) isoforms: differential deposition into the subepithelial extracellular matrix and bioactivity of extracellular matrix-bound VEGF. Mol Biol Cell. 1993;4:1317–26.

734. Park JB, Levine M. Cloning, sequencing, and characterization of alternatively spliced glutaredoxin 1 cDNA and its genomic gene: chromosomal localization, mrna stability, and origin of pseudogenes. J Biol Chem. 2005;280:10427–34.

735. Parrott AM, Walsh MR, Reichman TW, Mathews MB. RNA binding and phosphorylation determine the intracellular distribution of nuclear factors 90 and 110. J Mol Biol. 2005;348:281–93.

736. Patzke S, Redick S, Warsame A, Murga-Zamalloa CA, Khanna H, Doxsey S, et al. CSPP is a ciliary protein interacting with Nephrocystin 8 and required for cilia formation. Mol Biol Cell. 2010;21:2555–67.

737. Paulis YWJ, Huijbers EJM, van der Schaft DWJ, Soetekouw PMMB, Pauwels P, Tjan-Heijnen VCG,

et al. CD44 enhances tumor aggressiveness by promoting tumor cell plasticity. Oncotarget. 2015;6:19634–46.

738. Peacock SO, Fahrenholtz CD, Burnstein KL. Vav3 enhances androgen receptor splice variant activity and is critical for castration-resistant prostate cancer growth and survival. Mol Endocrinol. 2012;26:1967–79.

739. Pekova S, Cmejla R, Smolej L, Kozak T, Spacek M, Prucha M. Identification of a novel, transactivation-defective splicing variant of p53 gene in patients with chronic lymphocytic leukemia. Leuk Res. 2008;32:395–400.

740. Pekova S, Mazal O, Cmejla R, Hardekopf DW, Plachy R, Zejskova L, et al. A comprehensive study of TP53 mutations in chronic lymphocytic leukemia: Analysis of 1287 diagnostic and 1148 follow-up CLL samples. Leuk Res. 2011;35:889–98.

741. Pelley JL, Nicholls CD, Beattie TL, Brown CB. Discovery and characterization of a novel splice variant of the GM-CSF receptor alpha subunit. Exp Hematol. 2007;35:1483–94.

742. Pelosi M, Marampon F, Zani BM, Prudente S, Perlas E, Caputo V, et al. ROCK2 and Its Alternatively Spliced Isoform ROCK2m Positively Control the Maturation of the Myogenic Program. Mol Cell Biol. 2007;27:6163–76.

743. Peneff C, Ferrari P, Charrier V, Taburet Y, Monnier C, Zamboni V, et al. Crystal structures of two human pyrophosphorylase isoforms in complexes with UDPGlc(Gal)NAc: role of the alternatively spliced insert in the enzyme oligomeric assembly and active site architecture. EMBO J. 2001;20:6191–202.

744. Percival JM, Anderson KNE, Huang P, Adams ME, Froehner SC. Golgi and sarcolemmal neuronal NOS differentially regulate contraction-induced fatigue and vasoconstriction in exercising mouse skeletal muscle. J Clin Invest. 2010;120:816–26.

745. Pereira S, Massacrier A, Roll P, Vérine A, Etienne-Grimaldi M-C, Poitelon Y, et al. Nuclear localization of a novel human syntaxin 1B isoform. Gene. 2008;423:160–71.

746. Pérez-Gómez E, Eleno N, López-Novoa JM, Ramirez JR, Velasco B, Letarte M, et al. Characterization of murine S-endoglin isoform and its effects on tumor development. Oncogene. 2005;24:4450–61.

747. Perriaud L, Marcel V, Sagne C, Favaudon V, Guédin A, De Rache A, et al. Impact of G-quadruplex structures and intronic polymorphisms rs17878362 and rs1642785 on basal and ionizing radiation-induced expression of alternative p53 transcripts. Carcinogenesis. 2014;35:2706–15.

748. Perry C, Sklan EH, Birikh K, Shapira M, Trejo L, Eldor A, et al. Complex regulation of acetylcholinesterase gene expression in human brain tumors. Oncogene. 2002;21:8428–41.

749. Perry ME, Mendrysa SM, Saucedo LJ, Tannous P, Holubar M. p76(MDM2) inhibits the ability of p90(MDM2) to destabilize p53. J Biol Chem. 2000;275:5733–8.

750. Perumalsamy A, Fernandes R, Lai I, Detmar J, Varmuza S, Casper RF, et al. Developmental consequences of alternative Bcl-x splicing during preimplantation embryo development. FEBS J. 2010;277:1219–33.

751. Péterfy M, Phan J, Reue K. Alternatively spliced lipin isoforms exhibit distinct expression pattern, subcellular localization, and role in adipogenesis. J Biol Chem. 2005;280:32883–9.

752. Peters B, Kaiser HW, Magin TM. Skin-specific expression of ank-3(93), a novel ankyrin-3 splice variant. J Invest Dermatol. 2001;116:216–23.

753. Peters LL, John KM, Lu FM, Eicher EM, Higgins A, Yialamas M, et al. Ank3 (epithelial ankyrin), a widely distributed new member of the ankyrin gene family and the major ankyrin in kidney, is expressed in alternatively spliced forms, including forms that lack the repeat domain. J Cell Biol. 1995;130:313–30.

754. Petersen-Mahrt SK, Estmer C, Ohrmalm C, Matthews DA, Russell WC, Akusjärvi G. The splicing factor-associated protein, p32, regulates RNA splicing by inhibiting ASF/SF2 RNA binding and phosphorylation. EMBO J. 1999;18:1014–24.

755. Phanish MK, Heidebrecht F, Nabi ME, Shah N, Niculescu-Duvaz I, Dockrell MEC. The Regulation of TGFβ1 Induced Fibronectin EDA Exon Alternative Splicing in Human Renal Proximal Tubule Epithelial Cells. Journal of Cellular Physiology. 2015;230:286–95.

756. Philibert P, Audran F, Pienkowski C, Morange I, Kohler B, Flori E, et al. Complete androgen insensitivity syndrome is frequently due to premature stop codons in exon 1 of the androgen receptor gene: an international collaborative report of 13 new mutations. Fertil Steril. 2010;94:472–6.

757. Piccione EC, Lieu TJ, Gentile CF, Williams TR, Connolly AJ, Godwin AK, et al. A novel epidermal growth factor receptor variant lacking multiple domains directly activates transcription and is overexpressed in tumors. Oncogene. 2012;31:2953–67.

758. Piekielko-Witkowska A, Kedzierska H, Poplawski P, Wojcicka A, Rybicka B, Maksymowicz M, et al. Alternative splicing of iodothyronine deiodinases in pituitary adenomas. Regulation by oncoprotein SF2/ASF. Biochimica et Biophysica Acta (BBA) - Molecular Basis of Disease. 2013;1832:763–72.

759. Pineda-Lucena A, Ho CSW, Mao DYL, Sheng Y, Laister RC, Muhandiram R, et al. A structure-based model of the c-Myc/Bin1 protein interaction shows alternative splicing of Bin1 and c-Myc phosphorylation are key binding determinants. J Mol Biol. 2005;351:182–94.

760. Pirnie SP, Osman A, Zhu Y, Carmichael GG. An Ultraconserved Element (UCE) controls homeostatic splicing of ARGLU1 mRNA. Nucleic Acids Res. 2017;45:3473–86.

761. Pizon V, Iakovenko A, Van Der Ven PFM, Kelly R, Fatu C, Fürst DO, et al. Transient association of titin and myosin with microtubules in nascent myofibrils directed by the MURF2 RING-finger protein. J Cell Sci. 2002;115 Pt 23:4469–82.

762. Plötz M, Gillissen B, Quast S-A, Berger A, Daniel PT, Eberle J. The BH3-only protein Bim(L) overrides Bcl-2-mediated apoptosis resistance in melanoma cells. Cancer Lett. 2013;335:100–8.

763. Polfus LM, Khajuria RK, Schick UM, Pankratz N, Pazoki R, Brody JA, et al. Whole-Exome Sequencing Identifies Loci Associated with Blood Cell Traits and Reveals a Role for Alternative GFI1B Splice Variants in Human Hematopoiesis. The American Journal of Human Genetics. 2016;99:481–8.

764. Policha A, Daneshtalab N, Chen L, Dale LB, Altier C, Khosravani H, et al. Role of angiotensin II type 1A receptor phosphorylation, phospholipase D, and extracellular calcium in isoform-specific protein kinase C membrane translocation responses. J Biol Chem. 2006;281:26340–9.

765. Pollard AJ, Krainer AR, Robson SC, Europe-Finner GN. Alternative splicing of the adenylyl cyclase stimulatory G-protein G alpha(s) is regulated by SF2/ASF and heterogeneous nuclear

ribonucleoprotein A1 (hnRNPA1) and involves the use of an unusual TG 3'-splice Site. J Biol Chem. 2002;277:15241–51.

766. Porcelli V, Fiermonte G, Longo A, Palmieri F. The Human Gene SLC25A29, of Solute Carrier Family 25, Encodes a Mitochondrial Transporter of Basic Amino Acids. J Biol Chem. 2014;289:13374–84.

767. Potter C, Zhu W, Razafsky D, Ruzycki P, Kolesnikov AV, Doggett T, et al. Multiple Isoforms of Nesprin1 Are Integral Components of Ciliary Rootlets. Current Biology. doi:10.1016/j.cub.2017.05.066.

768. Poukkula M, Kaunisto A, Hietakangas V, Denessiouk K, Katajamäki T, Johnson MS, et al. Rapid turnover of c-FLIPshort is determined by its unique C-terminal tail. J Biol Chem. 2005;280:27345–55.

769. Poulsen KA, Pedersen SF, Kolko M, Lambert IH. Induction of group VIA phospholipase A2 activity during in vitro ischemia in C2C12 myotubes is associated with changes in the level of its splice variants. Am J Physiol, Cell Physiol. 2007;293:C1605-1615.

770. Powell DJ, Hrstka R, Candeias M, Bourougaa K, Vojtesek B, Fåhraeus R. Stress-dependent changes in the properties of p53 complexes by the alternative translation product p53/47. Cell Cycle. 2008;7:950–9.

771. Prabhakar BS, Mulherkar N, Prasad KV. Role of IG20 splice variants in TRAIL resistance. Clin Cancer Res. 2008;14:347–51.

772. Prasannan P, Appling DR. Human mitochondrial C1-tetrahydrofolate synthase: submitochondrial localization of the full-length enzyme and characterization of a short isoform. Arch Biochem Biophys. 2009;481:86–93.

773. Premont RT, Macrae AD, Stoffel RH, Chung N, Pitcher JA, Ambrose C, et al. Characterization of the G protein-coupled receptor kinase GRK4. Identification of four splice variants. J Biol Chem. 1996;271:6403–10.

774. Pretorius PR, Aldahmesh MA, Alkuraya FS, Sheffield VC, Slusarski DC. Functional analysis of BBS3 A89V that results in non-syndromic retinal degeneration. Hum Mol Genet. 2011;20:1625–32.

775. Pretorius PR, Baye LM, Nishimura DY, Searby CC, Bugge K, Yang B, et al. Identification and Functional Analysis of the Vision-Specific BBS3 (ARL6) Long Isoform. PLOS Genetics. 2010;6:e1000884.

776. Prevost JM, Pelley JL, Zhu W, D'Egidio GE, Beaudry PP, Pihl C, et al. Granulocyte-macrophage colony-stimulating factor (GM-CSF) and inflammatory stimuli up-regulate secretion of the soluble GM-CSF receptor in human monocytes: evidence for ectodomain shedding of the cell surface GM-CSF receptor alpha subunit. J Immunol. 2002;169:5679–88.

777. Prou D, Gu WJ, Le Crom S, Vincent JD, Salamero J, Vernier P. Intracellular retention of the two isoforms of the D(2) dopamine receptor promotes endoplasmic reticulum disruption. J Cell Sci. 2001;114 Pt 19:3517–27.

778. Purcell DF, Russell SM, Deacon NJ, Brown MA, Hooker DJ, McKenzie IF. Alternatively spliced RNAs encode several isoforms of CD46 (MCP), a regulator of complement activation. Immunogenetics. 1991;33:335–44.

779. Qi J, Su Y, Sun R, Zhang F, Luo X, Yang Z, et al. CASK inhibits ECV304 cell growth and interacts

with Id1. Biochem Biophys Res Commun. 2005;328:517–21.

780. Qin S, Zheng F, Chen G-H, Fang H, Wang X-M, Zhou J-N. Variable alternative spliced exon (VASE)-containing and VASE-lacking neural cell adhesion molecule in the dorsal and ventral hippocampus of SAMP8 mice. J Neurosci Res. 2005;80:838–44.

781. Quayle SN, Sadar MD. A truncated isoform of TMEFF2 encodes a secreted protein in prostate cancer cells. Genomics. 2006;87:633–7.

782. Quijada P, Hariharan N, Cubillo JD, Bala KM, Emathinger JM, Wang BJ, et al. Nuclear Calcium/Calmodulin-dependent Protein Kinase II Signaling Enhances Cardiac Progenitor Cell Survival and Cardiac Lineage Commitment. J Biol Chem. 2015;290:25411–26.

783. Radomska KJ, Halvardson J, Reinius B, Lindholm Carlström E, Emilsson L, Feuk L, et al. RNA-binding protein QKI regulates Glial fibrillary acidic protein expression in human astrocytes. Hum Mol Genet. 2013;22:1373–82.

784. Raharjo SB, Emoto N, Ikeda K, Sato R, Yokoyama M, Matsuo M. Alternative splicing regulates the endoplasmic reticulum localization or secretion of soluble secreted endopeptidase. J Biol Chem. 2001;276:25612–20.

785. Ram Kumar RM, Betz MM, Robl B, Born W, Fuchs B. ΔNp63α enhances the oncogenic phenotype of osteosarcoma cells by inducing the expression of GLI2. BMC Cancer. 2014;14:559.

786. Ramiro AR, Navarro MN, Carreira A, Carrasco YR, de Yébenes VG, Carrillo G, et al. Differential developmental regulation and functional effects on pre-TCR surface expression of human pTalpha(a) and pTalpha(b) spliced isoforms. J Immunol. 2001;167:5106–14.

787. Ramos-Morales F, Vime C, Bornens M, Fedriani C, Rios RM. Two splice variants of Golgi-microtubule-associated protein of 210 kDa (GMAP-210) differ in their binding to the cis-Golgi network. Biochem J. 2001;357 Pt 3:699–708.

788. Rao N, Nguyen S, Ngo K, Fung-Leung W-P. A novel splice variant of interleukin-1 receptor (IL-1R)-associated kinase 1 plays a negative regulatory role in Toll/IL-1R-induced inflammatory signaling. Mol Cell Biol. 2005;25:6521–32.

789. Rappe U, Schlechter T, Aschoff M, Hotz-Wagenblatt A, Hofmann I. Nuclear ARVCF Protein Binds Splicing Factors and Contributes to the Regulation of Alternative Splicing. J Biol Chem. 2014;289:12421–34.

790. Rauschendorf M-A, Zimmer J, Ohnmacht C, Vogt PH. DDX3X, the X homologue of AZFa gene DDX3Y, expresses a complex pattern of transcript variants only in the male germ line. Mol Hum Reprod. 2014;20:1208–22.

791. Rauscher FJ, Morris JF, Tournay OE, Cook DM, Curran T. Binding of the Wilms' tumor locus zinc finger protein to the EGR-1 consensus sequence. Science. 1990;250:1259–62.

792. Reddy JC, Morris JC, Wang J, English MA, Haber DA, Shi Y, et al. WT1-mediated transcriptional activation is inhibited by dominant negative mutant proteins. J Biol Chem. 1995;270:10878–84.

793. Regan PM, Sariyer IK, Langford TD, Datta PK, Khalili K. Morphine-induced MOR-1X and ASF/SF2 expression is independent of transcriptional regulation: Implications for MOR-1X signaling. J Cell Physiol. 2016;231:1542–53.

794. Regelmann AG, Danzl NM, Wanjalla C, Alexandropoulos K. The Hematopoietic Isoform of Cas-

Hef1-Associated Signal Transducer Regulates Chemokine-Induced Inside-Out Signaling and T Cell Trafficking. Immunity. 2006;25:907–18.

795. Reisine T, Kong H, Raynor K, Yano H, Takeda J, Yasuda K, et al. Splice variant of the somatostatin receptor 2 subtype, somatostatin receptor 2B, couples to adenylyl cyclase. Mol Pharmacol. 1993;44:1016–20.

796. Reissner C, Klose M, Fairless R, Missler M. Mutational analysis of the neurexin/neuroligin complex reveals essential and regulatory components. Proc Natl Acad Sci U S A. 2008;105:15124–9.

797. Ren D, Collingwood TN, Rebar EJ, Wolffe AP, Camp HS. PPARγ knockdown by engineered transcription factors: exogenous PPARγ2 but not PPARγ1 reactivates adipogenesis. Genes Dev. 2002;16:27–32.

798. Ren S, Nguyen L, Wu S, Encinas C, Adams JS, Hewison M. Alternative splicing of vitamin D-24-hydroxylase: a novel mechanism for the regulation of extrarenal 1,25-dihydroxyvitamin D synthesis. J Biol Chem. 2005;280:20604–11.

799. Renshaw SA, Dempsey CE, Barnes FA, Bagstaff SM, Dower SK, Bingle CD, et al. Three novel Bid proteins generated by alternative splicing of the human Bid gene. J Biol Chem. 2004;279:2846–55.

800. Rettig J, Sheng ZH, Kim DK, Hodson CD, Snutch TP, Catterall WA. Isoform-specific interaction of the alpha1A subunits of brain Ca2+ channels with the presynaptic proteins syntaxin and SNAP-25. Proc Natl Acad Sci USA. 1996;93:7363–8.

801. Richard DJ, Schumacher V, Royer-Pokora B, Roberts SG. Par4 is a coactivator for a splice isoform-specific transcriptional activation domain in WT1. Genes Dev. 2001;15:328–39.

802. Riley JH, Edbrooke MR, Craig RK. Ectopic synthesis of high-Mr calcitonin by the BEN lung carcinoma cell line reflects aberrant proteolytic processing. FEBS Lett. 1986;198:71–9.

803. Rindler TN, Lasko VM, Nieman ML, Okada M, Lorenz JN, Lingrel JB. Knockout of the Na,K-ATPase α2 isoform in cardiac myocytes delays pressure overload-induced cardiac dysfunction. American Journal of Physiology - Heart and Circulatory Physiology. 2013;304:H1147–58.

804. Riteau B, Rouas-Freiss N, Menier C, Paul P, Dausset J, Carosella ED. HLA-G2, -G3, and -G4 isoforms expressed as nonmature cell surface glycoproteins inhibit NK and antigen-specific CTL cytolysis. J Immunol. 2001;166:5018–26.

805. Roberts S, Calautti E, Vanderweil S, Nguyen HO, Foley A, Baden HP, et al. Changes in localization of human discs large (hDlg) during keratinocyte differentiation are [corrected] associated with expression of alternatively spliced hDlg variants. Exp Cell Res. 2007;313:2521–30.

806. Rocha-Sanchez SMS, Morris KA, Kachar B, Nichols D, Fritzsch B, Beisel KW. Developmental expression of Kcnq4 in vestibular neurons and neurosensory epithelia. Brain Res. 2007;1139:117–25.

807. Rodríguez-Carballo E, Gámez B, Sedó-Cabezón L, Sánchez-Feutrie M, Zorzano A, Manzanares-Céspedes C, et al. The p38α MAPK Function in Osteoprecursors Is Required for Bone Formation and Bone Homeostasis in Adult Mice. PLOS ONE. 2014;9:e102032.

808. Rodríguez-Malavé NI, Fernando TR, Patel PC, Contreras JR, Palanichamy JK, Tran TM, et al. BALR-6 regulates cell growth and cell survival in B-lymphoblastic leukemia. Mol Cancer. 2015;14:214.

809. Rohaly G, Chemnitz J, Dehde S, Nunez AM, Heukeshoven J, Deppert W, et al. A novel human p53 isoform is an essential element of the ATR-intra-S phase checkpoint. Cell. 2005;122:21–32.

810. Romanish MT, Nakamura H, Lai CB, Wang Y, Mager DL. A Novel Protein Isoform of the Multicopy Human NAIP Gene Derives from Intragenic Alu SINE Promoters. PLOS ONE. 2009;4:e5761.

811. Ronaldson E, Robertson DN, Johnson MS, Holland PJ, Mitchell R, Lutz EM. Specific interaction between the hop1 intracellular loop 3 domain of the human PAC(1) receptor and ARF. Regul Pept. 2002;109:193–8.

812. Rong Z, Ren Y, Cheng L, Li Z, Li Y, Sun Y, et al. Sef-S, an alternative splice isoform of sef gene, inhibits NIH3T3 cell proliferation via a mitogen-activated protein kinases p42 and p44 (ERK1/2)-independent mechanism. Cell Signal. 2007;19:93–102.

813. Rösel-Hillgärtner TD, Hung L-H, Khrameeva E, Le Querrec P, Gelfand MS, Bindereif A. A novel intra-U1 snRNP cross-regulation mechanism: alternative splicing switch links U1C and U1-70K expression. PLoS Genet. 2013;9:e1003856.

814. Rosenberg KJ, Ross JL, Feinstein HE, Feinstein SC, Israelachvili J. Complementary dimerization of microtubule-associated tau protein: Implications for microtubule bundling and tau-mediated pathogenesis. Proc Natl Acad Sci USA. 2008;105:7445–50.

815. Rosso L, Marques AC, Weier M, Lambert N, Lambot M-A, Vanderhaeghen P, et al. Birth and rapid subcellular adaptation of a hominoid-specific CDC14 Protein. PLOS Biology. 2008;6:e140.

816. Rouleau M, Roberge J, Bellemare J, Guillemette C. Dual roles for splice variants of the glucuronidation pathway as regulators of cellular metabolism. Mol Pharmacol. 2014;85:29–36.

817. Rousseaud A, Delépine C, Nectoux J, Billuart P, Bienvenu T. Differential expression and regulation of brain-derived neurotrophic factor (BDNF) mRNA isoforms in brain cells from Mecp2308/y mouse model. J Mol Neurosci. 2015;56:758–67.

818. Rowen L, Young J, Birditt B, Kaur A, Madan A, Philipps DL, et al. Analysis of the human neurexin genes: alternative splicing and the generation of protein diversity. Genomics. 2002;79:587–97.

819. Ruan G-X, Barry E, Yu D, Lukason M, Cheng SH, Scaria A. CRISPR/Cas9-mediated genome editing as a therapeutic approach for Leber congenital amaurosis 10. Mol Ther. 2017;25:331–41.

820. Rueckert EH, Barker D, Ruderfer D, Bergen SE, O’Dushlaine C, Luce CJ, et al. Cis-acting regulation of brain-specific ANK3 gene expression by a genetic variant associated with bipolar disorder. Mol Psychiatry. 2013;18:922–9.

821. Ruggeri P, Farina AR, Di Ianni N, Cappabianca L, Ragone M, Ianni G, et al. The TrkAIII oncoprotein inhibits mitochondrial free radical ROS-induced death of SH-SY5Y neuroblastoma cells by augmenting SOD2 expression and activity at the mitochondria, within the context of a tumour stem cell-like phenotype. PLoS ONE. 2014;9:e94568.

822. Ruppert SM, Li W, Zhang G, Carlson AL, Limaye A, Durum SK, et al. The major isoforms of Bim contribute to distinct biological activities that govern the processes of autophagy and apoptosis in interleukin-7 dependent lymphocytes. Biochim Biophys Acta. 2012;1823:1877–93.

823. Sabatino L, Casamassimi A, Peluso G, Barone MV, Capaccio D, Migliore C, et al. A novel peroxisome proliferator-activated receptor gamma isoform with dominant negative activity generated by alternative splicing. J Biol Chem. 2005;280:26517–25.

824. Sadakata T, Shinoda Y, Oka M, Sekine Y, Sato Y, Saruta C, et al. Reduced axonal localization of a Caps2 splice variant impairs axonal release of BDNF and causes autistic-like behavior in mice. Proc Natl Acad Sci USA. 2012;109:21104–9.

825. Sadakata T, Washida M, Iwayama Y, Shoji S, Sato Y, Ohkura T, et al. Autistic-like phenotypes in Cadps2-knockout mice and aberrant CADPS2 splicing in autistic patients. J Clin Invest. 2007;117:931–43.

826. Sagne C, Marcel V, Bota M, Martel-Planche G, Nobrega A, Palmero EI, et al. Age at cancer onset in germline TP53 mutation carriers: association with polymorphisms in predicted G-quadruplex structures. Carcinogenesis. 2014;35:807–15.

827. Saillour Y, Broix L, Bruel-Jungerman E, Lebrun N, Muraca G, Rucci J, et al. Beta tubulin isoforms are not interchangeable for rescuing impaired radial migration due to Tubb3 knockdown. Hum Mol Genet. 2014;23:1516–26.

828. Sakai Y, Oda H, Yoshimura D, Furuichi M, Kang D, Iwai S, et al. The GT to GC single nucleotide polymorphism at the beginning of an alternative exon 2C of human MTH1 gene confers an amino terminal extension that functions as a mitochondrial targeting signal. J Mol Med. 2006;84:660–70.

829. Sakurai Y, Onishi Y, Tanimoto Y, Kizaki H. Novel protein kinase C delta isoform insensitive to caspase-3. Biol Pharm Bull. 2001;24:973–7.

830. Sánchez-Aguilera A, García JF, Sánchez-Beato M, Piris MA. Hodgkin's lymphoma cells express alternatively spliced forms of HDM2 with multiple effects on cell cycle control. Oncogene. 2006;25:2565–74.

831. Sangha N, Wu R, Kuick R, Powers S, Mu D, Fiander D, et al. Neurofibromin 1 (NF1) defects are common in human ovarian serous carcinomas and co-occur with TP53 mutations. Neoplasia. 2008;10:1362–72, following 1372.

832. Sarkar A, Caamano S, Fernandez JM. The elasticity of individual titin PEVK exons measured by single molecule atomic force microscopy. J Biol Chem. 2005;280:6261–4.

833. Sarkissian M, Winne A, Lafyatis R. The mammalian homolog of suppressor-of-white-apricot regulates alternative mRNA splicing of CD45 exon 4 and fibronectin IIICS. J Biol Chem. 1996;271:31106–14.

834. Sasaki M, Miyosawa K, Ohkubo S, Nakahata N. Physiological significance of thromboxane A(2) receptor dimerization. J Pharmacol Sci. 2006;100:263–70.

835. Sato F, Yasumoto K, Kimura K, Numayama-Tsuruta K, Sogawa K. Heterodimerization with LBP-1b is necessary for nuclear localization of LBP-1a and LBP-1c. Genes Cells. 2005;10:861–70.

836. Saunders LR, Jurecic V, Barber GN. The 90- and 110-kDa human NFAR proteins are translated from two differentially spliced mRNAs encoded on chromosome 19p13. Genomics. 2001;71:256–9.

837. Saunders LR, Perkins DJ, Balachandran S, Michaels R, Ford R, Mayeda A, et al. Characterization of two evolutionarily conserved, alternatively spliced nuclear phosphoproteins, NFAR-1 and -2, that function in mRNA processing and interact with the double-stranded RNA-dependent protein kinase, PKR. J Biol Chem. 2001;276:32300–12.

838. Schardt JA, Weber D, Eyholzer M, Mueller BU, Pabst T. Activation of the unfolded protein response is associated with favorable prognosis in acute myeloid leukemia. Clin Cancer Res.

2009;15:3834–41.

839. Scharnhorst V, Dekker P, van der Eb AJ, Jochemsen AG. Physical interaction between Wilms tumor 1 and p73 proteins modulates their functions. J Biol Chem. 2000;275:10202–11.

840. Schenk RL, Tuzlak S, Carrington EM, Zhan Y, Heinzel S, Teh CE, et al. Characterisation of mice lacking all functional isoforms of the pro-survival BCL-2 family member A1 reveals minor defects in the haematopoietic compartment. Cell Death Differ. 2017;24:534–45.

841. Schiel MA, Green S, Davis WI, Sanghani PC, Bosron WF, Sanghani SP. Expression and characterization of a human carboxylesterase 2 splice variant. J Pharmacol Exp Ther. 2007;323:94–101.

842. Schinke T, Liese S, Priemel M, Haberland M, Schilling AF, Catala-Lehnen P, et al. Decreased bone formation and osteopenia in mice lacking alpha-calcitonin gene-related peptide. J Bone Miner Res. 2004;19:2049–56.

843. Schlesinger F, Tammena D, Krampfl K, Bufler J. Desensitization and resensitization are independently regulated in human recombinant GluR subunit coassemblies. Synapse. 2005;55:176–82.

844. Schneider C, Boeglin WE, Brash AR. Human cyclo-oxygenase-1 and an alternative splice variant: contrasts in expression of mRNA, protein and catalytic activities. Biochem J. 2005;385 Pt 1:57–64.

845. Schnerwitzki D, Perner B, Hoppe B, Pietsch S, Mehringer R, Hänel F, et al. Alternative splicing of Wilms tumor suppressor 1 (Wt1) exon 4 results in protein isoforms with different functions. Dev Biol. 2014;393:24–32.

846. Schwarzenbach H, Eichelser C, Steinbach B, Tadewaldt J, Pantel K, Lobanenkov V, et al. Differential regulation of MAGE-A1 promoter activity by BORIS and Sp1, both interacting with the TATA binding protein. BMC Cancer. 2014;14:796.

847. Scotlandi K, Zuntini M, Manara MC, Sciandra M, Rocchi A, Benini S, et al. CD99 isoforms dictate opposite functions in tumour malignancy and metastases by activating or repressing c-Src kinase activity. Oncogene. 2007;26:6604–18.

848. Scott DJ, Tregear GW, Bathgate R a. D. LGR7-truncate is a splice variant of the relaxin receptor LGR7 and is a relaxin antagonist in vitro. Ann N Y Acad Sci. 2005;1041:22–6.

849. Scotton P, Bleckmann D, Stebler M, Sciandra F, Brancaccio A, Meier T, et al. Activation of muscle-specific receptor tyrosine kinase and binding to dystroglycan are regulated by alternative mRNA splicing of agrin. J Biol Chem. 2006;281:36835–45.

850. Seiz Preianò B, Guerini D, Carafoli E. Expression and Functional Characterization of Isoforms 4 of the Plasma Membrane Calcium Pump. Biochemistry. 1996;35:7946–53.

851. Seng A, Mitchell JL, Yankee TM. Expression and splicing of Ikaros family members in murine and human thymocytes. The Journal of Immunology. 2017;198 1 Supplement:60.11-60.11.

852. Senturk S, Yao Z, Camiolo M, Stiles B, Rathod T, Walsh AM, et al. p53Ψ is a transcriptionally inactive p53 isoform able to reprogram cells toward a metastatic-like state. Proc Natl Acad Sci USA. 2014;111:E3287-3296.

853. Seo P-S, Jeong J-J, Zeng L, Takoudis CG, Quinn BJ, Khan AA, et al. Alternatively spliced exon 5 of

the FERM domain of protein 4.1R encodes a novel binding site for erythrocyte p55 and is critical for membrane targeting in epithelial cells. Biochim Biophys Acta. 2009;1793:281–9.

854. Seo SY, Chen Y-B, Ivanovska I, Ranger AM, Hong SJ, Dawson VL, et al. BAD is a pro-survival factor prior to activation of its pro-apoptotic function. J Biol Chem. 2004;279:42240–9.

855. Sepp M, Kannike K, Eesmaa A, Urb M, Timmusk T. Functional diversity of human basic helix-loop-helix transcription factor TCF4 isoforms generated by alternative 5' exon usage and splicing. PLoS ONE. 2011;6:e22138.

856. Serova O, Montagna M, Torchard D, Narod SA, Tonin P, Sylla B, et al. A high incidence of BRCA1 mutations in 20 breast-ovarian cancer families. Am J Hum Genet. 1996;58:42–51.

857. Shafi AA, Putluri V, Arnold JM, Tsouko E, Maity S, Roberts JM, et al. Differential regulation of metabolic pathways by androgen receptor (AR) and its constitutively active splice variant, AR-V7, in prostate cancer cells. Oncotarget. 2015;6:31997–2012.

858. Shah ZH, Ahmed SU, Ford JR, Allison SJ, Knight JRP, Milner J. A deacetylase-deficient SIRT1 variant opposes full-length SIRT1 in regulating tumor suppressor p53 and governs expression of cancer-related genes. Mol Cell Biol. 2012;32:704–16.

859. Shao H, Zhu C, Zhao Z, Guo M, Qiu H, Liu H, et al. KRAB-containing zinc finger gene ZNF268 encodes multiple alternatively spliced isoforms that contain transcription regulatory domains. Int J Mol Med. 2006;18:457–63.

860. Shao L, Liu L, Miao Z, Ren H, Wang W, Lang Y, et al. A novel SLC12A3 splicing mutation skipping of two exons and preliminary screening for alternative splice variants in human kidney. Am J Nephrol. 2008;28:900–7.

861. Sharp DA, Lawrence DA, Ashkenazi A. Selective knockdown of the long variant of cellular FLICE inhibitory protein augments death receptor-mediated caspase-8 activation and apoptosis. J Biol Chem. 2005;280:19401–9.

862. Shaul YD, Seger R. ERK1c regulates Golgi fragmentation during mitosis. J Cell Biol. 2006;172:885–97.

863. Sheng W, Wang G, Wang Y, Liang J, Wen J, Zheng P-S, et al. The roles of versican V1 and V2 isoforms in cell proliferation and apoptosis. Mol Biol Cell. 2005;16:1330–40.

864. Shi B, Triebe D, Kajiji S, Iwata KK, Bruskin A, Mahajna J. Identification and characterization of baxepsilon, a novel bax variant missing the BH2 and the transmembrane domains. Biochem Biophys Res Commun. 1999;254:779–85.

865. Shi J, Zhang T, Zhou C, Chohan MO, Gu X, Wegiel J, et al. Increased dosage of Dyrk1A alters alternative splicing factor (ASF)-regulated alternative splicing of tau in Down syndrome. J Biol Chem. 2008;283:28660–9.

866. Shi L, Chang X, Zhang P, Coba MP, Lu W, Wang K. The functional genetic link of NLGN4X knockdown and neurodevelopment in neural stem cells. Hum Mol Genet. 2013;22:3749–60.

867. Shia W-J, Okumura AJ, Yan M, Sarkeshik A, Lo M-C, Matsuura S, et al. PRMT1 interacts with AML1-ETO to promote its transcriptional activation and progenitor cell proliferative potential. Blood. 2012;119:4953–62.

868. Shieh J-J, Liu K-T, Huang S-W, Chen Y-J, Hsieh T-Y. Modification of alternative splicing of Mcl-1

pre-mRNA using antisense morpholino oligonucleotides induces apoptosis in basal cell carcinoma cells. J Invest Dermatol. 2009;129:2497–506.

869. Shikama N, Lee CW, France S, Delavaine L, Lyon J, Krstic-Demonacos M, et al. A novel cofactor for p300 that regulates the p53 response. Mol Cell. 1999;4:365–76.

870. Shimokawa T, Tostar U, Lauth M, Palaniswamy R, Kasper M, Toftgård R, et al. Novel human glioma-associated oncogene 1 (GLI1) splice variants reveal distinct mechanisms in the terminal transduction of the hedgehog signal. J Biol Chem. 2008;283:14345–54.

871. Shingleton JR, Hemann MT. The Chromatin Regulator CHD8 Is a Context-Dependent Mediator of Cell Survival in Murine Hematopoietic Malignancies. PLOS ONE. 2015;10:e0143275.

872. Shiote Y, Ouchida M, Jitsumori Y, Ogama Y, Matsuo Y, Ishimaru F, et al. Multiple splicing variants of Naf1/ABIN-1 transcripts and their alterations in hematopoietic tumors. Int J Mol Med. 2006;18:917–23.

873. Shishikura M, Nakamura F, Yamashita N, Uetani N, Iwakura Y, Goshima Y. Expression of receptor protein tyrosine phosphatase δ, PTPδ, in mouse central nervous system. Brain Research. 2016;1642:244–54.

874. Shkreta L, Michelle L, Toutant J, Tremblay ML, Chabot B. The DNA damage response pathway regulates the alternative splicing of the apoptotic mediator Bcl-x. J Biol Chem. 2011;286:331–40.

875. Shu L, Yan W, Chen X. RNPC1, an RNA-binding protein and a target of the p53 family, is required for maintaining the stability of the basal and stress-induced p21 transcript. Genes Dev. 2006;20:2961–72.

876. Shultz JC, Goehe RW, Murudkar CS, Wijesinghe DS, Mayton EK, Massiello A, et al. SRSF1 regulates the alternative splicing of caspase 9 via a novel intronic splicing enhancer affecting the chemotherapeutic sensitivity of non-small cell lung cancer cells. Mol Cancer Res. 2011;9:889–900.

877. Shultz JC, Goehe RW, Wijesinghe DS, Murudkar C, Hawkins AJ, Shay JW, et al. Alternative splicing of caspase 9 is modulated by the phosphoinositide 3-kinase/Akt pathway via phosphorylation of SRp30a. Cancer Res. 2010;70:9185–96.

878. Siewiera J, Gouilly J, Hocine H-R, Cartron G, Levy C, Al-Daccak R, et al. Natural cytotoxicity receptor splice variants orchestrate the distinct functions of human natural killer cell subtypes. Nat Commun. 2015;6:10183.

879. Simons A, Melamed-Bessudo C, Wolkowicz R, Sperling J, Sperling R, Eisenbach L, et al. PACT: cloning and characterization of a cellular p53 binding protein that interacts with Rb. Oncogene. 1997;14:145–55.

880. Singh R, Gupta SC, Peng W-X, Zhou N, Pochampally R, Atfi A, et al. Regulation of alternative splicing of Bcl-x by BC200 contributes to breast cancer pathogenesis. Cell Death Dis. 2016;7:e2262.

881. Singh SK, Wilczynska KM, Grzybowski A, Yester J, Osrah B, Bryan L, et al. The Unique Transcriptional Activation Domain of Nuclear Factor-I-X3 Is Critical to Specifically Induce Marker Gene Expression in Astrocytes. J Biol Chem. 2011;286:7315–26.

882. Siu I-M, Bai R, Gallia GL, Edwards JB, Tyler BM, Eberhart CG, et al. Coexpression of neuronatin splice forms promotes medulloblastoma growth. Neuro-oncology. 2008;10:716–24.

883. Slaaby R, Schäffer L, Lautrup-Larsen I, Andersen AS, Shaw AC, Mathiasen IS, et al. Hybrid receptors formed by insulin receptor (IR) and insulin-like growth factor I receptor (IGF-IR) have low insulin and high IGF-1 affinity irrespective of the IR splice variant. J Biol Chem. 2006;281:25869–74.

884. Small TW, Pickering JG. Nuclear Degradation of Wilms Tumor 1-associating Protein and Survivin Splice Variant Switching Underlie IGF-1-mediated Survival. J Biol Chem. 2009;284:24684–95.

885. Smith DE, Lipsky BP, Russell C, Ketchem RR, Kirchner J, Hensley K, et al. A central nervous system-restricted isoform of the interleukin-1 receptor accessory protein modulates neuronal responses to interleukin-1. Immunity. 2009;30:817–31.

886. Sogawa C, Kumagai K, Sogawa N, Morita K, Dohi T, Kitayama S. C-terminal region regulates the functional expression of human noradrenaline transporter splice variants. Biochem J. 2007;401:185–95.

887. Sogawa C, Mitsuhata C, Kumagai-Morioka K, Sogawa N, Ohyama K, Morita K, et al. Expression and function of variants of human catecholamine transporters lacking the fifth transmembrane region encoded by exon 6. PLoS ONE. 2010;5:e11945.

888. Sohail M, Zhang M, Litchfield D, Wang L, Kung S, Xie J. Differential expression, distinct localization and opposite effect on Golgi structure and cell differentiation by a novel splice variant of human PRMT5. Biochim Biophys Acta. 2015;1853 10 Pt A:2444–52.

889. Solberg R, Sandberg M, Natarajan V, Torjesen PA, Hansson V, Jahnsen T, et al. The human gene for the regulatory subunit RI alpha of cyclic adenosine 3', 5'-monophosphate-dependent protein kinase: two distinct promoters provide differential regulation of alternately spliced messenger ribonucleic acids. Endocrinology. 1997;138:169–81.

890. Soldatov NM, Bouron A, Reuter H. Different voltage-dependent inhibition by dihydropyridines of human Ca2+ channel splice variants. J Biol Chem. 1995;270:10540–3.

891. Soldatov NM, Zühlke RD, Bouron A, Reuter H. Molecular structures involved in L-type calcium channel inactivation. Role of the carboxyl-terminal region encoded by exons 40-42 in alpha1C subunit in the kinetics and Ca2+ dependence of inactivation. J Biol Chem. 1997;272:3560–6.

892. Soleymanlou N, Wu Y, Wang JX, Todros T, Ietta F, Jurisicova A, et al. A novel Mtd splice isoform is responsible for trophoblast cell death in pre-eclampsia. Cell Death Differ. 2005;12:441–52.

893. Song SW, Fuller GN, Zheng H, Zhang W. Inactivation of the invasion inhibitory gene IIp45 by alternative splicing in gliomas. Cancer Res. 2005;65:3562–7.

894. Soreq H, Seidman S. Acetylcholinesterase--new roles for an old actor. Nat Rev Neurosci. 2001;2:294–302.

895. Soupene E, Dinh NP, Siliakus M, Kuypers FA. Activity of the acyl-CoA synthetase ACSL6 isoforms: role of the fatty acid Gate-domains. BMC Biochem. 2010;11:18.

896. Speek M, Njunkova O, Pata I, Valdre E, Kogerman P. A potential role of alternative splicing in the regulation of the transcriptional activity of human GLI2 in gonadal tissues. BMC Mol Biol. 2006;7:13.

897. Spena S, Tenchini ML, Buratti E. Cryptic splice site usage in exon 7 of the human fibrinogen Bbeta-chain gene is regulated by a naturally silent SF2/ASF binding site within this exon. RNA. 2006;12:948–58.

898. Spiesbach K, Tannapfel A, Mössner J, Engeland K. TAp63gamma can substitute for p53 in inducing expression of the maspin tumor suppressor. Int J Cancer. 2005;114:555–62.

899. Spiess M, Lodish HF. An internal signal sequence: the asialoglycoprotein receptor membrane anchor. Cell. 1986;44:177–85.

900. Spillantini MG, Murrell JR, Goedert M, Farlow MR, Klug A, Ghetti B. Mutation in the tau gene in familial multiple system tauopathy with presenile dementia. Proc Natl Acad Sci USA. 1998;95:7737–41.

901. Sporici RA, Hodskins JS, Locasto DM, Meszaros LB, Ferry AL, Weidner AM, et al. Repression of the prolactin promoter: a functional consequence of the heterodimerization between Pit-1 and Pit-1 beta. J Mol Endocrinol. 2005;35:317–31.

902. Sporn JC, Jung B. Differential regulation and predictive potential of MacroH2A1 isoforms in colon cancer. Am J Pathol. 2012;180:2516–26.

903. Srinivasula SM, Ahmad M, Guo Y, Zhan Y, Lazebnik Y, Fernandes-Alnemri T, et al. Identification of an endogenous dominant-negative short isoform of caspase-9 that can regulate apoptosis. Cancer Res. 1999;59:999–1002.

904. Stamps AC, Elmore MA, Hill ME, Kelly K, Makda AA, Finnen MJ. A human cDNA sequence with homology to non-mammalian lysophosphatidic acid acyltransferases. Biochem J. 1997;326 (Pt 2):455–61.

905. Stauffer TP, Hilfiker H, Carafoli E, Strehler EE. Quantitative analysis of alternative splicing options of human plasma membrane calcium pump genes. J Biol Chem. 1993;268:25993–6003.

906. Staversky RJ, Vitiello PF, Yee M, Callahan LM, Dean DA, O'Reilly MA. Epithelial ablation of Bcl-XL increases sensitivity to oxygen without disrupting lung development. Am J Respir Cell Mol Biol. 2010;43:376–85.

907. Stocker JW, Nadasdi L, Aldrich RW, Tsien RW. Preferential interaction of omega-conotoxins with inactivated N-type Ca2+ channels. J Neurosci. 1997;17:3002–13.

908. Stockley J, Markert E, Zhou Y, Robson CN, Elliott DJ, Lindberg J, et al. The RNA-binding protein Sam68 regulates expression and transcription function of the androgen receptor splice variant AR-V7. Sci Rep. 2015;5:13426.

909. Stoilov P, Castren E, Stamm S. Analysis of the human TrkB gene genomic organization reveals novel TrkB isoforms, unusual gene length, and splicing mechanism. Biochem Biophys Res Commun. 2002;290:1054–65.

910. Strehler EE, James P, Fischer R, Heim R, Vorherr T, Filoteo AG, et al. Peptide sequence analysis and molecular cloning reveal two calcium pump isoforms in the human erythrocyte membrane. J Biol Chem. 1990;265:2835–42.

911. Strichman-Almashanu LZ, Bustin M, Landsman D. Retroposed copies of the HMG genes: a window to genome dynamics. Genome Res. 2003;13:800–12.

912. Ström A-L, Forsgren L, Holmberg M. Identification and characterization of Spinocerebellar Ataxia Type 7 (SCA7) isoform SCA7b in mice. Biochim Biophys Acta. 2005;1731:149–53.

913. Stros M, Ozaki T, Bacikova A, Kageyama H, Nakagawara A. HMGB1 and HMGB2 cell-specifically down-regulate the p53- and p73-dependent sequence-specific transactivation from the human

Bax gene promoter. J Biol Chem. 2002;277:7157–64.

914. Su B, Martin MM, Beason KB, Miller PJ, Elton TS. The genomic organization and functional analysis of the promoter for the human angiotensin II type 1 receptor. Biochem Biophys Res Commun. 1994;204:1039–46.

915. Suenaga K, Lee K-Y, Nakamori M, Tatsumi Y, Takahashi MP, Fujimura H, et al. Muscleblind-Like 1 Knockout Mice Reveal Novel Splicing Defects in the Myotonic Dystrophy Brain. PLOS ONE. 2012;7:e33218.

916. Sun F, Chen H, Li W, Yang X, Wang X, Jiang R, et al. Androgen receptor splice variant AR3 promotes prostate cancer via modulating expression of autocrine/paracrine factors. J Biol Chem. 2014;289:1529–39.

917. Sun S, Zhang Z, Sinha R, Karni R, Krainer AR. SF2/ASF autoregulation involves multiple layers of post-transcriptional and translational control. Nat Struct Mol Biol. 2010;17:306–12.

918. Sun X, Jin Z, Song X, Wang J, Li Y, Qian X, et al. Evaluation of KIF23 variant 1 expression and relevance as a novel prognostic factor in patients with hepatocellular carcinoma. BMC Cancer. 2015;15:961.

919. Sun YF, Yu LY, Saarma M, Timmusk T, Arumae U. Neuron-specific Bcl-2 homology 3 domain-only splice variant of Bak is anti-apoptotic in neurons, but pro-apoptotic in non-neuronal cells. J Biol Chem. 2001;276:16240–7.

920. Sundvall M, Peri L, Määttä JA, Tvorogov D, Paatero I, Savisalo M, et al. Differential nuclear localization and kinase activity of alternative ErbB4 intracellular domains. Oncogene. 2007;26:6905–14.

921. Sundvall M, Korhonen A, Paatero I, Gaudio E, Melino G, Croce CM, et al. Isoform-specific monoubiquitination, endocytosis, and degradation of alternatively spliced ErbB4 isoforms. Proc Natl Acad Sci USA. 2008;105:4162–7.

922. Sutherland C, Alterio J, Campbell DG, Le Bourdellès B, Mallet J, Haavik J, et al. Phosphorylation and activation of human tyrosine hydroxylase in vitro by mitogen-activated protein (MAP) kinase and MAP-kinase-activated kinases 1 and 2. Eur J Biochem. 1993;217:715–22.

923. Sutherland LC, Thibault P, Durand M, Lapointe E, Knee JM, Beauvais A, et al. Splicing arrays reveal novel RBM10 targets, including SMN2 pre-mRNA. BMC Molecular Biology. 2017;18:19.

924. Tabaczewski P, Shirwan H, Lewis K, Stroynowski I. Alternative splicing of class Ib major histocompatibility complex transcripts in vivo leads to the expression of soluble Qa-2 molecules in murine blood. Proc Natl Acad Sci USA. 1994;91:1883–7.

925. Tabata Y, Chen W, Warrier MR, Gibson AM, Daines MO, Hershey GKK. Allergy-driven alternative splicing of IL-13 receptor alpha2 yields distinct membrane and soluble forms. J Immunol. 2006;177:7905–12.

926. Tadokoro K, Yamazaki-Inoue M, Tachibana M, Fujishiro M, Nagao K, Toyoda M, et al. Frequent occurrence of protein isoforms with or without a single amino acid residue by subtle alternative splicing: the case of Gln in DRPLA affects subcellular localization of the products. J Hum Genet. 2005;50:382–94.

927. Tagami T, Yamamoto H, Moriyama K, Sawai K, Usui T, Shimatsu A, et al. Identification of a novel human thyroid hormone receptor beta isoform as a transcriptional modulator. Biochem Biophys

Res Commun. 2010;396:983–8.

928. Tago K, Noda T, Hayakawa M, Iwahana H, Yanagisawa K, Yashiro T, et al. Tissue distribution and subcellular localization of a variant form of the human ST2 gene product, ST2V. Biochem Biophys Res Commun. 2001;285:1377–83.

929. Takagi M, Ohashi K, Morimura T, Sugimoto C, Onuma M. The presence of the p53 transcripts with truncated open reading frames in Marek's disease tumor-derived cell lines. Leuk Res. 2006;30:987–92.

930. Takagi S, Ueda Y, Hijikata M, Shimotohno K. Overproduced p73alpha activates a minimal promoter through a mechanism independent of its transcriptional activity. FEBS Lett. 2001;509:47–52.

931. Takahashi H, Chen MC, Pham H, Matsuo Y, Ishiguro H, Reber HA, et al. Simultaneous knock-down of Bcl-xL and Mcl-1 induces apoptosis through Bax activation in pancreatic cancer cells. Biochim Biophys Acta. 2013;1833:2980–7.

932. Takasawa S, Kuroki M, Nata K, Noguchi N, Ikeda T, Yamauchi A, et al. A novel ryanodine receptor expressed in pancreatic islets by alternative splicing from type 2 ryanodine receptor gene. Biochem Biophys Res Commun. 2010;397:140–5.

933. Takeo K, Kawai T, Nishida K, Masuda K, Teshima-Kondo S, Tanahashi T, et al. Oxidative stress-induced alternative splicing of transformer 2beta (SFRS10) and CD44 pre-mRNAs in gastric epithelial cells. Am J Physiol, Cell Physiol. 2009;297:C330-338.

934. Talarico EF. Plasma membrane calcium-ATPase isoform four distribution changes during corneal epithelial wound healing. Mol Vis. 2010;16:2259–72.

935. Tamborini E, Della Torre G, Lavarino C, Azzarelli A, Carpinelli P, Pierotti MA, et al. Analysis of the molecular species generated by MDM2 gene amplification in liposarcomas. Int J Cancer. 2001;92:790–6.

936. Tan D, Tan S, Zhang J, Tang P, Huang J, Zhou W, et al. Histone trimethylation of the p53 gene by expression of a constitutively active prolactin receptor in prostate cancer cells. Chin J Physiol. 2013;56:282–90.

937. Tan D, Walker AM. Short form 1b human prolactin receptor down-regulates expression of the long form. J Mol Endocrinol. 2010;44:187–94.

938. Tan GMY, Yu D, Wang J, Soong TW. Alternative splicing at C terminus of CaV1.4 calcium channel modulates calcium-dependent inactivation, activation potential, and current density. J Biol Chem. 2012;287:832–47.

939. Tanahashi H, Tabira T. Three novel alternatively spliced isoforms of the human beta-site amyloid precursor protein cleaving enzyme (BACE) and their effect on amyloid beta-peptide production. Neurosci Lett. 2001;307:9–12.

940. Tanaka S, Saito K, Reed JC. Structure-function analysis of the Bcl-2 oncoprotein. Addition of a heterologous transmembrane domain to portions of the Bcl-2 beta protein restores function as a regulator of cell survival. J Biol Chem. 1993;268:10920–6.

941. Tang X, Tian Z, Chueh P-J, Chen S, Morré DM, Morré DJ. Alternative splicing as the basis for specific localization of tNOX, a unique hydroquinone (NADH) oxidase, to the cancer cell surface. Biochemistry. 2007;46:12337–46.

942. Tang Y, Horikawa I, Ajiro M, Robles AI, Fujita K, Mondal AM, et al. Downregulation of splicing factor SRSF3 induces p53β, an alternatively spliced isoform of p53 that promotes cellular senescence. Oncogene. 2013;32:2792–8.

943. Tang ZZ, Sharma S, Zheng S, Chawla G, Nikolic J, Black DL. Regulation of the mutually exclusive exons 8a and 8 in the CaV1.2 calcium channel transcript by polypyrimidine tract-binding protein. J Biol Chem. 2011;286:10007–16.

944. Tange TO, Jensen TH, Kjems J. In vitro interaction between human immunodeficiency virus type 1 Rev protein and splicing factor ASF/SF2-associated protein, p32. J Biol Chem. 1996;271:10066–72.

945. Tanowitz M, Hislop JN, von Zastrow M. Alternative splicing determines the post-endocytic sorting fate of G-protein-coupled receptors. J Biol Chem. 2008;283:35614–21.

946. Tanti GK, Goswami SK. SG2NA recruits DJ-1 and Akt into the mitochondria and membrane to protect cells from oxidative damage. Free Radical Biology and Medicine. 2014;75:1–13.

947. Tao R-H, Kawate H, Ohnaka K, Ishizuka M, Hagiwara H, Takayanagi R. Opposite effects of alternative TZF spliced variants on androgen receptor. Biochem Biophys Res Commun. 2006;341:515–21.

948. Taranta A, Petrini S, Palma A, Mannucci L, Wilmer MJ, De Luca V, et al. Identification and subcellular localization of a new cystinosin isoform. Am J Physiol Renal Physiol. 2008;294:F1101-1108.

949. Tardos JG, Eisenreich A, Deikus G, Bechhofer DH, Chandradas S, Zafar U, et al. SR proteins ASF/SF2 and SRp55 participate in tissue factor biosynthesis in human monocytic cells. Journal of Thrombosis and Haemostasis. 2008;6:877–84.

950. Taylor JK, Zhang QQ, Wyatt JR, Dean NM. Induction of endogenous Bcl-xS through the control of Bcl-x pre-mRNA splicing by antisense oligonucleotides. Nat Biotechnol. 1999;17:1097–100.

951. Teixeira A, Yen B, Gusella GL, Thomas AG, Mullen MP, Aberg J, et al. Prothymosin α variants isolated from CD8+ T cells and cervicovaginal fluid suppress HIV-1 replication through type I interferon induction. J Infect Dis. 2015;211:1467–75.

952. Thakur S, Zhang HB, Peng Y, Le H, Carroll B, Ward T, et al. Localization of BRCA1 and a splice variant identifies the nuclear localization signal. Mol Cell Biol. 1997;17:444–52.

953. Tian L, Duncan RR, Hammond MS, Coghill LS, Wen H, Rusinova R, et al. Alternative splicing switches potassium channel sensitivity to protein phosphorylation. J Biol Chem. 2001;276:7717–20.

954. Tiran Z, Oren A, Hermesh C, Rotman G, Levine Z, Amitai H, et al. A novel recombinant soluble splice variant of Met is a potent antagonist of the hepatocyte growth factor/scatter factor-Met pathway. Clin Cancer Res. 2008;14:4612–21.

955. Tiwari S, Zhang Y, Heller J, Abernethy DR, Soldatov NM. Atherosclerosis-related molecular alteration of the human CaV1.2 calcium channel alpha1C subunit. Proc Natl Acad Sci USA. 2006;103:17024–9.

956. Toda M, Suzuki T, Hosono K, Kurihara Y, Kurihara H, Hayashi I, et al. Roles of calcitonin gene-related peptide in facilitation of wound healing and angiogenesis. Biomed Pharmacother. 2008;62:352–9.

957. Tom Tang Y, Emtage P, Funk WD, Hu T, Arterburn M, Park EEJ, et al. TAFA: a novel secreted family with conserved cysteine residues and restricted expression in the brain. Genomics. 2004;83:727–34.

958. Tomida M. Presence of mRNAs encoding the soluble D-factor/LIF receptor in human choriocarcinoma cells and production of the soluble receptor. Biochem Biophys Res Commun. 1997;232:427–31.

959. Tomlinson DC, L'Hôte CG, Kennedy W, Pitt E, Knowles MA. Alternative splicing of fibroblast growth factor receptor 3 produces a secreted isoform that inhibits fibroblast growth factor-induced proliferation and is repressed in urothelial carcinoma cell lines. Cancer Res. 2005;65:10441–9.

960. Tone M, Tone Y, Fairchild PJ, Wykes M, Waldmann H. Regulation of CD40 function by its isoforms generated through alternative splicing. Proc Natl Acad Sci USA. 2001;98:1751–6.

961. Tong L, Wu S. ROS and p53 in regulation of UVB-induced HDM2 alternative splicing. Photochem Photobiol. 2015;91:221–4.

962. Tonnelle C, Bardin F, Maroc C, Imbert AM, Campa F, Dalloul A, et al. Forced expression of the Ikaros 6 isoform in human placental blood CD34(+) cells impairs their ability to differentiate toward the B-lymphoid lineage. Blood. 2001;98:2673–80.

963. Tournillon A-S, López I, Malbert-Colas L, Naski N, Olivares-Illana V, Fåhraeus R. The alternative translated MDMX(p60) isoform regulates MDM2 activity. Cell Cycle. 2015;14:449–58.

964. Tran YH, Xu Z, Kato A, Mistry AC, Goya Y, Taira M, et al. Spliced isoforms of LIM-domain-binding protein (CLIM/NLI/Ldb) lacking the LIM-interaction domain. J Biochem. 2006;140:105–19.

965. Treeck O, Pfeiler G, Horn F, Federhofer B, Houlihan H, Vollmer A, et al. Novel estrogen receptor beta transcript variants identified in human breast cancer cells affect cell growth and apoptosis of COS-1 cells. Mol Cell Endocrinol. 2007;264:50–60.

966. Tripathi V, Ellis JD, Shen Z, Song DY, Pan Q, Watt AT, et al. The nuclear-retained noncoding RNA MALAT1 regulates alternative splicing by modulating SR splicing factor phosphorylation. Mol Cell. 2010;39:925–38.

967. Tsai K-W, Tseng H-C, Lin W-C. Two wobble-splicing events affect ING4 protein subnuclear localization and degradation. Exp Cell Res. 2008;314:3130–41.

968. Tsatsaris V, Tarrade A, Merviel P, Garel JM, Segond N, Jullienne A, et al. Calcitonin gene-related peptide (CGRP) and CGRP receptor expression at the human implantation site. J Clin Endocrinol Metab. 2002;87:4383–90.

969. Tse S-W, Broderick JA, Wei M-L, Luo M-H, Smith D, McCaffery P, et al. Identification, expression analysis, genomic organization and cellular location of a novel protein with a RhoGEF domain. Gene. 2005;359:63–72.

970. Tsujimoto Y, Croce CM. Analysis of the structure, transcripts, and protein products of bcl-2, the gene involved in human follicular lymphoma. Proc Natl Acad Sci USA. 1986;83:5214–8.

971. Tsyba L, Gryaznova T, Dergai O, Dergai M, Skrypkina I, Kropyvko S, et al. Alternative splicing affecting the SH3A domain controls the binding properties of intersectin 1 in neurons. Biochem Biophys Res Commun. 2008;372:929–34.

972. Tuluc P, Molenda N, Schlick B, Obermair GJ, Flucher BE, Jurkat-Rott K. A CaV1.1 Ca2+ channel

splice variant with high conductance and voltage-sensitivity alters EC coupling in developing skeletal muscle. Biophys J. 2009;96:35–44.

973. Tutakhel OAZ, Jeleń S, Valdez-Flores M, Dimke H, Piersma SR, Jimenez CR, et al. Alternative splice variant of the thiazide-sensitive NaCl cotransporter: a novel player in renal salt handling. Am J Physiol Renal Physiol. 2016;310:F204-216.

974. U M, Miyashita T, Shikama Y, Tadokoro K, Yamada M. Molecular cloning and characterization of six novel isoforms of human Bim, a member of the proapoptotic Bcl-2 family. FEBS Lett. 2001;509:135–41.

975. Uchikawa H, Toyoda M, Nagao K, Miyauchi H, Nishikawa R, Fujii K, et al. Brain- and heart-specific Patched-1 containing exon 12b is a dominant negative isoform and is expressed in medulloblastomas. Biochem Biophys Res Commun. 2006;349:277–83.

976. Ueyama T, Lekstrom K, Tsujibe S, Saito N, Leto TL. Subcellular localization and function of alternatively spliced Noxo1 isoforms. Free Radic Biol Med. 2007;42:180–90.

977. Unoki M, Shen JC, Zheng Z-M, Harris CC. Novel splice variants of ING4 and their possible roles in the regulation of cell growth and motility. J Biol Chem. 2006;281:34677–86.

978. Uo T, Dworzak J, Kinoshita C, Inman DM, Kinoshita Y, Horner PJ, et al. Drp1 levels constitutively regulate mitochondrial dynamics and cell survival in cortical neurons. Exp Neurol. 2009;218:274–85.

979. Upheber S, Karle A, Miller J, Schlaugk S, Gross E, Reuning U. Alternative splicing of KAI1 abrogates its tumor-suppressive effects on integrin αvβ3-mediated ovarian cancer biology. Cell Signal. 2015;27:652–62.

980. Urano Y, Iiduka M, Sugiyama A, Akiyama H, Uzawa K, Matsumoto G, et al. Involvement of the mouse Prp19 gene in neuronal/astroglial cell fate decisions. J Biol Chem. 2006;281:7498–514.

981. Usiello A, Baik J-H, Rougé-Pont F, Picetti R, Dierich A, LeMeur M, et al. Distinct functions of the two isoforms of dopamine D2 receptors. Nature. 2000;408:199–203.

982. Vaghi V, Polacchini A, Baj G, Pinheiro VLM, Vicario A, Tongiorgi E. Pharmacological Profile of Brain-derived Neurotrophic Factor (BDNF) Splice Variant Translation Using a Novel Drug Screening Assay A “QUANTITATIVE CODE.” J Biol Chem. 2014;289:27702–13.

983. Vallejo-Illarramendi A, Domercq M, Matute C. A novel alternative splicing form of excitatory amino acid transporter 1 is a negative regulator of glutamate uptake. J Neurochem. 2005;95:341–8.

984. van den Berg YW, van den Hengel LG, Myers HR, Ayachi O, Jordanova E, Ruf W, et al. Alternatively spliced tissue factor induces angiogenesis through integrin ligation. Proc Natl Acad Sci USA. 2009;106:19497–502.

985. van der Voort R, Verweij V, de Witte TM, Lasonder E, Adema GJ, Dolstra H. An alternatively spliced CXCL16 isoform expressed by dendritic cells is a secreted chemoattractant for CXCR6+ cells. J Leukoc Biol. 2010;87:1029–39.

986. Van Itallie CM, Tietgens AJ, Krystofiak E, Kachar B, Anderson JM. A complex of ZO-1 and the BAR-domain protein TOCA-1 regulates actin assembly at the tight junction. Mol Biol Cell. 2015;26:2769–87.

987. Vasta V, Sonnenburg WK, Yan C, Soderling SH, Shimizu-Albergine M, Beavo JA. Identification of a

new variant of PDE1A calmodulin-stimulated cyclic nucleotide phosphodiesterase expressed in mouse sperm. Biol Reprod. 2005;73:598–609.

988. Vatter P, Stoesser C, Samel I, Gierschik P, Moepps B. The variable C-terminal extension of G-protein-coupled receptor kinase 6 constitutes an accessorial autoregulatory domain. FEBS J. 2005;272:6039–51.

989. Vegran F, Boidot R, Oudin C, Riedinger J-M, Lizard-Nacol S. [Implication of alternative splice transcripts of caspase-3 and survivin in chemoresistance]. Bull Cancer. 2005;92:219–26.

990. Végran F, Boidot R, Oudin C, Riedinger J-M, Bonnetain F, Lizard-Nacol S. Overexpression of caspase-3s splice variant in locally advanced breast carcinoma is associated with poor response to neoadjuvant chemotherapy. Clin Cancer Res. 2006;12:5794–800.

991. Venables JP, Bourgeois CF, Dalgliesh C, Kister L, Stevenin J, Elliott DJ. Up-regulation of the ubiquitous alternative splicing factor Tra2beta causes inclusion of a germ cell-specific exon. Hum Mol Genet. 2005;14:2289–303.

992. Vendel AC, Terry MD, Striegel AR, Iverson NM, Leuranguer V, Rithner CD, et al. Alternative splicing of the voltage-gated Ca2+ channel beta4 subunit creates a uniquely folded N-terminal protein binding domain with cell-specific expression in the cerebellar cortex. J Neurosci. 2006;26:2635–44.

993. Vendelin J, Pulkkinen V, Rehn M, Pirskanen A, Räisänen-Sokolowski A, Laitinen A, et al. Characterization of GPRA, a novel G protein-coupled receptor related to asthma. Am J Respir Cell Mol Biol. 2005;33:262–70.

994. Verduci L, Simili M, Rizzo M, Mercatanti A, Evangelista M, Mariani L, et al. MicroRNA (miRNA)-mediated interaction between leukemia/lymphoma-related factor (LRF) and alternative splicing factor/splicing factor 2 (ASF/SF2) affects mouse embryonic fibroblast senescence and apoptosis. J Biol Chem. 2010;285:39551–63.

995. Verissimo CS, Molenaar JJ, Meerman J, Puigvert JC, Lamers F, Koster J, et al. Silencing of the microtubule-associated proteins doublecortin-like and doublecortin-like kinase-long induces apoptosis in neuroblastoma cells. Endocr Relat Cancer. 2010;17:399–414.

996. Vernes SC, Nicod J, Elahi FM, Coventry JA, Kenny N, Coupe A-M, et al. Functional genetic analysis of mutations implicated in a human speech and language disorder. Hum Mol Genet. 2006;15:3154–67.

997. Villar VAM, Jones JE, Armando I, Palmes-Saloma C, Yu P, Pascua AM, et al. G Protein-coupled Receptor Kinase 4 (GRK4) Regulates the Phosphorylation and Function of the Dopamine D3 Receptor. J Biol Chem. 2009;284:21425–34.

998. Vliet V, I A, Baelde HJ, Vleming L-J, de Heer E, Anthonie Bruijn J. Distribution of fibronectin isoforms in human renal disease. The Journal of Pathology. 2001;193:256–62.

999. von Marschall Z, Fisher LW. Decorin is processed by three isoforms of bone morphogenetic protein-1 (BMP1). Biochemical and Biophysical Research Communications. 2010;391:1374–8.

1000. von Schack D, Casademunt E, Schweigreiter R, Meyer M, Bibel M, Dechant G. Complete ablation of the neurotrophin receptor p75NTR causes defects both in the nervous and the vascular system. Nat Neurosci. 2001;4:977–8.

1001. Wada K, Tanji K, Kamitani T. Function and subcellular location of Ro52beta. Biochem Biophys Res

Commun. 2006;340:872–8.

1002. Wagner K, Kafert-Kasting S, Heil G, Ganser A, Eder M. Inhibition of granulocyte-macrophage colony-stimulating factor receptor function by a splice variant of the common beta-receptor subunit. Blood. 2001;98:2689–96.

1003. Wagner W, Fodor E, Ginsburg A, Hammer JA. The binding of DYNLL2 to myosin Va requires alternatively spliced exon B and stabilizes a portion of the myosin's coiled-coil domain. Biochemistry. 2006;45:11564–77.

1004. Walker SM, Downes CP, Leslie NR. TPIP: a novel phosphoinositide 3-phosphatase. Biochem J. 2001;360 Pt 2:277–83.

1005. Wallmen B, Schrempp M, Hecht A. Intrinsic properties of Tcf1 and Tcf4 splice variants determine cell-type-specific Wnt/β-catenin target gene expression. Nucleic Acids Res. 2012;40:9455–69.

1006. Walz HA, Shi X, Chouinard M, Bue CA, Navaroli DM, Hayakawa A, et al. Isoform-specific Regulation of Akt Signaling by the Endosomal Protein WDFY2. J Biol Chem. 2010;285:14101–8.

1007. Walzik S, Schroeter A, Benndorf K, Zimmer T. Alternative splicing of the cardiac sodium channel creates multiple variants of mutant T1620K channels. PLoS ONE. 2011;6:e19188.

1008. Wan W, Farboud B, Privalsky ML. Pituitary resistance to thyroid hormone syndrome is associated with T3 receptor mutants that selectively impair beta2 isoform function. Mol Endocrinol. 2005;19:1529–42.

1009. Wang F, Pan J, Liu Y, Meng Q, Lv P, Qu F, et al. Alternative splicing of the androgen receptor in polycystic ovary syndrome. Proc Natl Acad Sci USA. 2015;112:4743–8.

1010. Wang H-C, Yang Y, Xu S-Y, Peng J, Jiang J-H, Li C-Y. The CRISPR/Cas system inhibited the pro-oncogenic effects of alternatively spliced fibronectin extra domain A via editing the genome in salivary adenoid cystic carcinoma cells. Oral Dis. 2015;21:608–18.

1011. Wang H, Wang P, Sun X, Luo Y, Wang X, Ma D, et al. Cloning and characterization of a novel caspase-10 isoform that activates NF-kappa B activity. Biochim Biophys Acta. 2007;1770:1528–37.

1012. Wang J, Thio SSC, Yang SSH, Yu D, Yu CY, Wong YP, et al. Splice variant specific modulation of CaV1.2 calcium channel by galectin-1 regulates arterial constriction. Circ Res. 2011;109:1250–8.

1013. Wang L, Duke L, Zhang PS, Arlinghaus RB, Symmans WF, Sahin A, et al. Alternative splicing disrupts a nuclear localization signal in spleen tyrosine kinase that is required for invasion suppression in breast cancer. Cancer Res. 2003;63:4724–30.

1014. Wang L, Li G, Sugita S. A central kinase domain of type I phosphatidylinositol phosphate kinases is sufficient to prime exocytosis: isoform specificity and its underlying mechanism. J Biol Chem. 2005;280:16522–7.

1015. Wang MG, Yi H, Hilfiker H, Carafoli E, Strehler EE, McBride OW. Localization of two genes encoding plasma membrane Ca2+ ATPases isoforms 2 (ATP2B2) and 3 (ATP2B3) to human chromosomes 3p26-->p25 and Xq28, respectively. Cytogenet Cell Genet. 1994;67:41–5.

1016. Wang Q, O'Brien PJ, Chen CX, Cho DS, Murray JM, Nishikura K. Altered G protein-coupling functions of RNA editing isoform and splicing variant serotonin2C receptors. J Neurochem. 2000;74:1290–300.

1017. Wang Q, Chikina MD, Pincas H, Sealfon SC. Homer1 alternative splicing is regulated by gonadotropin-releasing hormone and modulates gonadotropin gene expression. Mol Cell Biol. 2014;34:1747–56.

1018. Wang W, Zhang H, Liu S, Kim CK, Xu Y, Hurley LA, et al. Internalized CD44s splice isoform attenuates EGFR degradation by targeting Rab7A. PNAS. 2017;:201701289.

1019. Wang X, Sheng P, Guo X, Wang J, Hou L, Hu G, et al. Identification and expression of a novel MDM4 splice variant in human glioma. Brain Res. 2013;1537:260–6.

1020. Wang X, Yang Y, Duan Q, Jiang N, Huang Y, Darzynkiewicz Z, et al. sSgo1, a major splice variant of Sgo1, functions in centriole cohesion where it is regulated by Plk1. Dev Cell. 2008;14:331–41.

1021. Wang Y, Bernhardy AJ, Cruz C, Krais JJ, Nacson J, Nicolas E, et al. The BRCA1-Δ11q Alternative Splice Isoform Bypasses Germline Mutations and Promotes Therapeutic Resistance to PARP Inhibition and Cisplatin. Cancer Res. 2016;76:2778–90.

1022. Wang Y, Li Y, Chen B, Zhang Y, Lou G, Chen S, et al. Identification and characterization of PNRC splicing variants. Gene. 2008;423:116–24.

1023. Wang Z-Z, Zhang Y, Liu Y-Q, Zhao N, Zhang Y-Z, Yuan L, et al. RNA interference-mediated phosphodiesterase 4D splice variants knock-down in the prefrontal cortex produces antidepressant-like and cognition-enhancing effects. Br J Pharmacol. 2013;168:1001–14.

1024. Wang Z, Jiang H, Chen S, Du F, Wang X. The mitochondrial phosphatase PGAM5 functions at the convergence point of multiple necrotic death pathways. Cell. 2012;148:228–43.

1025. Warner SMB, Hackett T-L, Shaheen F, Hallstrand TS, Kicic A, Stick SM, et al. Transcription factor p63 regulates key genes and wound repair in human airway epithelial basal cells. Am J Respir Cell Mol Biol. 2013;49:978–88.

1026. Watson PA, Chen YF, Balbas MD, Wongvipat J, Socci ND, Viale A, et al. Constitutively active androgen receptor splice variants expressed in castration-resistant prostate cancer require full-length androgen receptor. Proc Natl Acad Sci USA. 2010;107:16759–65.

1027. Wei X, Hao L, Ni S, Liu Q, Xu J, Correll PH. Altered exon usage in the juxtamembrane domain of mouse and human RON regulates receptor activity and signaling specificity. J Biol Chem. 2005;280:40241–51.

1028. Wei Y, Fu G, Hu H, Lin G, Yang J, Guo J, et al. Isolation and characterization of mouse testis specific serine/threonine kinase 5 possessing four alternatively spliced variants. J Biochem Mol Biol. 2007;40:749–56.

1029. Weichert W, Röske A, Niesporek S, Noske A, Buckendahl A-C, Dietel M, et al. Class I histone deacetylase expression has independent prognostic impact in human colorectal cancer: specific role of class I histone deacetylases in vitro and in vivo. Clin Cancer Res. 2008;14:1669–77.

1030. Weise A, Bruser K, Elfert S, Wallmen B, Wittel Y, Wöhrle S, et al. Alternative splicing of Tcf7l2 transcripts generates protein variants with differential promoter-binding and transcriptional activation properties at Wnt/beta-catenin targets. Nucleic Acids Res. 2010;38:1964–81.

1031. Werder A von, Mayr M, Schneider G, Oesterle D, Fritsch RM, Seidler B, et al. Truncated IRAG variants modulate cGMP-mediated inhibition of human colonic smooth muscle cell contraction. Am J Physiol, Cell Physiol. 2011;301:C1445-1457.

1032. Wethkamp N, Hanenberg H, Funke S, Suschek CV, Wetzel W, Heikaus S, et al. Daxx-beta and Daxx-gamma, two novel splice variants of the transcriptional co-repressor Daxx. J Biol Chem. 2011;286:19576–88.

1033. Wiedemann P, Bönisch H, Oerters F, Brüss M. Structure of the human histamine H3 receptor gene (HRH3) and identification of naturally occurring variations. J Neural Transm (Vienna). 2002;109:443–53.

1034. Wiesenthal A, Hoffmeister M, Siddique M, Kovacevic I, Oess S, Müller-Esterl W, et al. NOSTRINbeta - a shortened NOSTRIN variant with a role in transcriptional regulation. Traffic. 2009;10:26–34.

1035. Wiggins CM, Johnson M, Cook SJ. Refining the minimal sequence required for ERK1/2-dependent poly-ubiquitination and proteasome-dependent turnover of BIM. Cell Signal. 2010;22:801–8.

1036. Wilhelmi I, Kanski R, Neumann A, Herdt O, Hoff F, Jacob R, et al. Sec16 alternative splicing dynamically controls COPII transport efficiency. Nat Commun. 2016;7:12347.

1037. Wilson TR, McLaughlin KM, McEwan M, Sakai H, Rogers KMA, Redmond KM, et al. c-FLIP: a key regulator of colorectal cancer cell death. Cancer Res. 2007;67:5754–62.

1038. Wimberly H, Han G, Pinnaduwage D, Murphy LC, Yang XR, Andrulis IL, et al. ERβ splice variant expression in four large cohorts of human breast cancer patient tumors. Breast Cancer Res Treat. 2014;146:657–67.

1039. Wistow G, Bernstein SL, Wyatt MK, Behal A, Touchman JW, Bouffard G, et al. Expressed sequence tag analysis of adult human lens for the NEIBank Project: over 2000 non-redundant transcripts, novel genes and splice variants. Mol Vis. 2002;8:171–84.

1040. Wistow G, Bernstein SL, Wyatt MK, Fariss RN, Behal A, Touchman JW, et al. Expressed sequence tag analysis of human RPE/choroid for the NEIBank Project: over 6000 non-redundant transcripts, novel genes and splice variants. Mol Vis. 2002;8:205–20.

1041. Wollscheid H-P, Biancospino M, He F, Magistrati E, Molteni E, Lupia M, et al. Diverse functions of myosin VI elucidated by an isoform-specific α-helix domain. Nat Struct Mol Biol. 2016;23:300–8.

1042. Wong J, Hyde TM, Cassano HL, Deep-Soboslay A, Kleinman JE, Weickert CS. Promoter specific alterations of brain-derived neurotrophic factor mRNA in schizophrenia. Neuroscience. 2010;169:1071–84.

1043. Wong J, Garner B, Halliday GM, Kwok JBJ. Srp20 regulates TrkB pre-mRNA splicing to generate TrkB-Shc transcripts with implications for Alzheimer's disease. J Neurochem. 2012;123:159–71.

1044. Woodside DG, Kram RM, Mitchell JS, Belsom T, Billard MJ, McIntyre BW, et al. Contrasting roles for domain 4 of VCAM-1 in the regulation of cell adhesion and soluble VCAM-1 binding to integrin alpha4beta1. J Immunol. 2006;176:5041–9.

1045. Woolfson A, Milstein C. Alternative splicing generates secretory isoforms of human CD1. Proc Natl Acad Sci USA. 1994;91:6683–7.

1046. Worton LE, Shi Y-C, Smith EJ, Barry SC, Gonda TJ, Whitehead JP, et al. Ectodermal-Neural Cortex 1 Isoforms Have Contrasting Effects on MC3T3-E1 Osteoblast Mineralization and Gene Expression. J Cell Biochem. 2017;118:2141–50.

1047. Wu H-M, Schally AV, Cheng J-C, Zarandi M, Varga J, Leung PCK. Growth hormone-releasing

hormone antagonist induces apoptosis of human endometrial cancer cells through PKCδ-mediated activation of p53/p21. Cancer Letters. 2010;298:16–25.

1048. Wu JY, Kar A, Kuo D, Yu B, Havlioglu N. SRp54 (SFRS11), a regulator for tau exon 10 alternative splicing identified by an expression cloning strategy. Mol Cell Biol. 2006;26:6739–47.

1049. Wu L, Mao C, Ming X. Modulation of Bcl-x Alternative Splicing Induces Apoptosis of Human Hepatic Stellate Cells. Biomed Res Int. 2016;2016:7478650.

1050. Wu Y, Shi X, Guo S-W. The knockdown of progesterone receptor isoform B (PR-B) promotes proliferation in immortalized endometrial stromal cells. Fertility and Sterility. 2008;90:1320–3.

1051. Wu Y, Lu Y, Hu Y, Li R. Cyclic AMP-dependent modification of gonad-selective TAF(II)105 in a human ovarian granulosa cell line. J Cell Biochem. 2005;96:751–9.

1052. Wuebben EL, Mallanna SK, Cox JL, Rizzino A. Musashi2 is required for the self-renewal and pluripotency of embryonic stem cells. PLoS ONE. 2012;7:e34827.

1053. Xiao Q, Ford AL, Xu J, Yan P, Lee K-Y, Gonzales E, et al. Bcl-x pre-mRNA splicing regulates brain injury after neonatal hypoxia-ischemia. J Neurosci. 2012;32:13587–96.

1054. Xu CF, Brown MA, Chambers JA, Griffiths B, Nicolai H, Solomon E. Distinct transcription start sites generate two forms of BRCA1 mRNA. Hum Mol Genet. 1995;4:2259–64.

1055. Xu CF, Chambers JA, Nicolai H, Brown MA, Hujeirat Y, Mohammed S, et al. Mutations and alternative splicing of the BRCA1 gene in UK breast/ovarian cancer families. Genes Chromosomes Cancer. 1997;18:102–10.

1056. Xu D, Zhan Y, Qi Y, Cao B, Bai S, Xu W, et al. Androgen receptor splice variants dimerize to transactivate target genes. Cancer Res. 2015;75:3663–71.

1057. Xu H, El-Gewely MR. Differentially expressed downstream genes in cells with normal or mutated p53. Oncol Res. 2003;13:429–36.

1058. Xu T, Nie L, Zhang Y, Mo J, Feng W, Wei D, et al. Roles of alternative splicing in the functional properties of inner ear-specific KCNQ4 channels. J Biol Chem. 2007;282:23899–909.

1059. Xu X-M, Zhou Y-Q, Wang M-H. Mechanisms of cytoplasmic {beta}-catenin accumulation and its involvement in tumorigenic activities mediated by oncogenic splicing variant of the receptor originated from Nantes tyrosine kinase. J Biol Chem. 2005;280:25087–94.

1060. Xu XJ, Boumechache M, Robinson LE, Marschall V, Gorecki DC, Masin M, et al. Splice variants of the P2X7 receptor reveal differential agonist dependence and functional coupling with pannexin-1. J Cell Sci. 2012;125 Pt 16:3776–89.

1061. Xu Y, Zhang S, Malhotra A, Edelman-Novemsky I, Ma J, Kruppa A, et al. Characterization of tafazzin splice variants from humans and fruit flies. J Biol Chem. 2009;284:29230–9.

1062. Xu Z-X, Zhao R-X, Ding T, Tran TT, Zhang W, Pandolfi PP, et al. Promyelocytic leukemia protein 4 induces apoptosis by inhibition of survivin expression. J Biol Chem. 2004;279:1838–44.

1063. Yamaguchi H, Inokuchi K, Dan K. The study for loss of bcl-xs expression as a prognostic factor in acute myeloid leukemia. Leuk Res. 2002;26:1119–23.

1064. Yamamoto Y, Huibregtse JM, Howley PM. The human E6-AP gene (UBE3A) encodes three

potential protein isoforms generated by differential splicing. Genomics. 1997;41:263–6.

1065. Yamamoto Y, Loriot Y, Beraldi E, Zhang F, Wyatt AW, Al Nakouzi N, et al. Generation 2.5 antisense oligonucleotides targeting the androgen receptor and its splice variants suppress enzalutamide-resistant prostate cancer cell growth. Clin Cancer Res. 2015;21:1675–87.

1066. Yamankurt G, Wu HC, McCarthy M, Cunha SR. Exon organization and novel alternative splicing of Ank3 in mouse heart. PLoS ONE. 2015;10:e0128177.

1067. Yamashita S, Lai K-P, Chuang K-L, Xu D, Miyamoto H, Tochigi T, et al. ASC-J9 suppresses castration-resistant prostate cancer growth through degradation of full-length and splice variant androgen receptors. Neoplasia. 2012;14:74–83.

1068. Yan J, Noltner K, Feng J, Li W, Schroer R, Skinner C, et al. Neurexin 1alpha structural variants associated with autism. Neurosci Lett. 2008;438:368–70.

1069. Yan M, Kanbe E, Peterson LF, Boyapati A, Miao Y, Wang Y, et al. A previously unidentified alternatively spliced isoform of t(8;21) transcript promotes leukemogenesis. Nat Med. 2006;12:945–9.

1070. Yan W, Chen X. GPX2, a direct target of p63, inhibits oxidative stress-induced apoptosis in a p53-dependent manner. J Biol Chem. 2006;281:7856–62.

1071. Yang B-X, Duan Y-J, Dong C-Y, Zhang F, Gao W-F, Cui X-Y, et al. Novel Functions for mda-7/IL-24 and IL-24 delE5: Regulation of Differentiation of Acute Myeloid Leukemic Cells. Mol Cancer Ther. 2011;10:615–25.

1072. Yang L, Sakurai T, Kamiyoshi A, Ichikawa-Shindo Y, Kawate H, Yoshizawa T, et al. Endogenous CGRP protects against neointimal hyperplasia following wire-induced vascular injury. J Mol Cell Cardiol. 2013;59:55–66.

1073. Yang XL, Miura N, Kawarada Y, Terada K, Petrukhin K, Gilliam T, et al. Two forms of Wilson disease protein produced by alternative splicing are localized in distinct cellular compartments. Biochem J. 1997;326 (Pt 3):897–902.

1074. Yang X, Guo Z, Sun F, Li W, Alfano A, Shimelis H, et al. Novel membrane-associated androgen receptor splice variant potentiates proliferative and survival responses in prostate cancer cells. J Biol Chem. 2011;286:36152–60.

1075. Yap K, Xiao Y, Friedman BA, Je HS, Makeyev EV. Polarizing the Neuron through Sustained Co-expression of Alternatively Spliced Isoforms. Cell Rep. 2016.

1076. Yedavalli VSRK, Jeang K-T. Rev-ing up post-transcriptional HIV-1 RNA expression. RNA Biol. 2011;8:195–9.

1077. Yee CSK, Yao Y, Li P, Klemsz MJ, Blum JS, Chang C-H. Cathepsin E: a novel target for regulation by class II transactivator. The Journal of Immunology. 2004;172:5528–34.

1078. Yi P, Zhang W, Zhai Z, Miao L, Wang Y, Wu M. Bcl-rambo beta, a special splicing variant with an insertion of an Alu-like cassette, promotes etoposide- and Taxol-induced cell death. FEBS Lett. 2003;534:61–8.

1079. Yin F, Du Y, Hu W, Qiao T, Ding J, Wu K, et al. Mad2beta, an alternative variant of Mad2 reducing mitotic arrest and apoptosis induced by adriamycin in gastric cancer cells. Life Sci. 2006;78:1277–86.

1080. Yokohari K, Yamashita Y, Okada S, Ohya K, Oda S, Hatano M, et al. Isoform-dependent interaction of BRDG1 with Tec kinase. Biochem Biophys Res Commun. 2001;289:414–20.

1081. Yonezawa T, Chen K-HE, Ghosh MK, Rivera L, Dill R, Ma L, et al. Anti-metastatic outcome of isoform-specific prolactin receptor targeting in breast cancer. Cancer Lett. 2015;366:84–92.

1082. Yoshida H, Crowther RA, Goedert M. Functional effects of tau gene mutations deltaN296 and N296H. J Neurochem. 2002;80:548–51.

1083. Yoshida T, Shiroshima T, Lee S-J, Yasumura M, Uemura T, Chen X, et al. Interleukin-1 receptor accessory protein organizes neuronal synaptogenesis as a cell adhesion molecule. J Neurosci. 2012;32:2588–600.

1084. Yoshida T, Yasumura M, Uemura T, Lee S-J, Ra M, Taguchi R, et al. IL-1 receptor accessory protein-like 1 associated with mental retardation and autism mediates synapse formation by trans-synaptic interaction with protein tyrosine phosphatase δ. J Neurosci. 2011;31:13485–99.

1085. You D-J, Park CR, Lee HB, Moon MJ, Kang J-H, Lee C, et al. A splicing variant of NME1 negatively regulates NF-κB signaling and inhibits cancer metastasis by interacting with IKKβ. J Biol Chem. 2014;289:17709–20.

1086. Yu Q, Guo J, Zhou J. A minimal length between tau exon 10 and 11 is required for correct splicing of exon 10. J Neurochem. 2004;90:164–72.

1087. Yu W, Li R, Gui B, Shang Y. sZIP, an alternative splice variant of ZIP, antagonizes transcription repression and growth inhibition by ZIP. J Biol Chem. 2010;285:14301–7.

1088. Zacharias DA, DeMarco SJ, Strehler EE. mRNA expression of the four isoforms of the human plasma membrane Ca2+-ATPase in the human hippocampus. Molecular Brain Research. 1997;45:173–6.

1089. Zambon AC, Wilderman A, Ho A, Insel PA. Increased expression of the pro-apoptotic protein BIM, a mechanism for cAMP/protein kinase A (PKA)-induced apoptosis of immature T cells. J Biol Chem. 2011;286:33260–7.

1090. Zendman AJW, Van Kraats AA, Weidle UH, Ruiter DJ, Van Muijen GNP. The XAGE family of cancer/testis-associated genes: alignment and expression profile in normal tissues, melanoma lesions and Ewing’s sarcoma. Int J Cancer. 2002;99:361–9.

1091. Zeng L, Zhang P, Shi L, Yamamoto V, Lu W, Wang K. Functional Impacts of NRXN1 Knockdown on Neurodevelopment in Stem Cell Models. PLOS ONE. 2013;8:e59685.

1092. Zeng X, Xia X-M, Lingle CJ. Species-specific Differences among KCNMB3 BK beta3 auxiliary subunits: some beta3 N-terminal variants may be primate-specific subunits. J Gen Physiol. 2008;132:115–29.

1093. Zha X, Yan X, Shen Q, Zhang Y, Wu X, Chen S, et al. Alternative expression of TCRζ related genes in patients with chronic myeloid leukemia. J Hematol Oncol. 2012;5:74.

1094. Zhang B, Wang W, Zhang Z, Hu Y, Meng F, Wang F, et al. Alternative Splicing of Disabled-1 Controls Multipolar-to-Bipolar Transition of Migrating Neurons in the Neocortex. Cerebral Cortex. 2017;:1–11.

1095. Zhang D-L, Hughes RM, Ollivierre-Wilson H, Ghosh MC, Rouault TA. A Ferroportin Transcript that Lacks an Iron-Responsive Element Enables Duodenal and Erythroid Precursor Cells to Evade

Translational Repression. Cell Metabolism. 2009;9:461–73.

1096. Zhang G, Liu X, Li J, Ledet E, Alvarez X, Qi Y, et al. Androgen receptor splice variants circumvent AR blockade by microtubule-targeting agents. Oncotarget. 2015;6:23358–71.

1097. Zhang H, Heim J, Meyhack B. Novel BNIP1 variants and their interaction with BCL2 family members. FEBS Lett. 1999;448:23–7.

1098. Zhang L, Liu X, Sheng H, Liu S, Li Y, Zhao JQ, et al. Neuron-specific SUMO knockdown suppresses global gene expression response and worsens functional outcome after transient forebrain ischemia in mice. Neuroscience. 2017;343:190–212.

1099. Zhang P, Greendorfer JS, Jiao J, Kelpke SC, Thompson JA. Alternatively spliced FGFR-1 isoforms differentially modulate endothelial cell activation of c-YES. Arch Biochem Biophys. 2006;450:50–62.

1100. Zhang QH, Ye M, Wu XY, Ren SX, Zhao M, Zhao CJ, et al. Cloning and functional analysis of cDNAs with open reading frames for 300 previously undefined genes expressed in CD34+ hematopoietic stem/progenitor cells. Genome Res. 2000;10:1546–60.

1101. Zhang W, Bai T, Zhang S, Xu S, Chen H, Li C. Isoforms of the nuclear envelope protein Nurim are differentially expressed during heart development in mice. Gene. doi:10.1016/j.gene.2017.06.009.

1102. Zhang X, Moor AN, Merkler KA, Liu Q, McLean MP. Regulation of alternative splicing of liver scavenger receptor class B gene by estrogen and the involved regulatory splicing factors. Endocrinology. 2007;148:5295–304.

1103. Zhang X, Farrell AS, Daniel CJ, Arnold H, Scanlan C, Laraway BJ, et al. Mechanistic insight into Myc stabilization in breast cancer involving aberrant Axin1 expression. Proc Natl Acad Sci USA. 2012;109:2790–5.

1104. Zhang X, Azhar G, Huang C, Cui C, Zhong Y, Huck S, et al. Alternative splicing and nonsense-mediated mRNA decay regulate gene expression of serum response factor. Gene. 2007;400:131–9.

1105. Zhang X, Morrissey C, Sun S, Ketchandji M, Nelson PS, True LD, et al. Androgen receptor variants occur frequently in castration resistant prostate cancer metastases. PLoS ONE. 2011;6:e27970.

1106. Zhang Y, Huypens P, Adamson AW, Chang JS, Henagan TM, Boudreau A, et al. Alternative mRNA splicing produces a novel biologically active short isoform of PGC-1alpha. J Biol Chem. 2009;284:32813–26.

1107. Zhao C, Xu Z, Chen J, Yu Z, Tong K-L, Lo W-S, et al. Two isoforms of GABA(A) receptor beta2 subunit with different electrophysiological properties: Differential expression and genotypical correlations in schizophrenia. Mol Psychiatry. 2006;11:1092–105.

1108. Zhao X, Khurana S, Charkraborty S, Tian Y, Sedor JR, Bruggman LA, et al. α Actinin 4 (ACTN4) Regulates Glucocorticoid Receptor-mediated Transactivation and Transrepression in Podocytes. J Biol Chem. 2017;292:1637–47.

1109. Zheng CF, Guan KL. Properties of MEKs, the kinases that phosphorylate and activate the extracellular signal-regulated kinases. J Biol Chem. 1993;268:23933–9.

1110. Zheng S, Gray EE, Chawla G, Porse BT, O'Dell TJ, Black DL. PSD-95 is post-transcriptionally repressed during early neural development by PTBP1 and PTBP2. Nat Neurosci. 2012;15:381–8,

S1.

1111. Zhong X, Liu JR, Kyle JW, Hanck DA, Agnew WS. A profile of alternative RNA splicing and transcript variation of CACNA1H, a human T-channel gene candidate for idiopathic generalized epilepsies. Hum Mol Genet. 2006;15:1497–512.

1112. Zhou H-L, Baraniak AP, Lou H. Role for Fox-1/Fox-2 in mediating the neuronal pathway of calcitonin/calcitonin gene-related peptide alternative RNA processing. Mol Cell Biol. 2007;27:830–41.

1113. Zhou W, Liu Z, Wu J, Liu J, Hyder SM, Antoniou E, et al. Identification and Characterization of Two Novel Splicing Isoforms of Human Estrogen-Related Receptor β. J Clin Endocrinol Metab. 2006;91:569–79.

1114. Zhou X, Li X, Cheng Y, Wu W, Xie Z, Xi Q, et al. BCLAF1 and its splicing regulator SRSF10 regulate the tumorigenic potential of colon cancer cells. Nature Communications. 2014;5:ncomms5581.

1115. Zhu B, Ramachandran B, Gulick T. Alternative pre-mRNA splicing governs expression of a conserved acidic transactivation domain in myocyte enhancer factor 2 factors of striated muscle and brain. J Biol Chem. 2005;280:28749–60.

1116. Zhu H, Guo W, Zhang L, Davis JJ, Teraishi F, Wu S, et al. Bcl-XL small interfering RNA suppresses the proliferation of 5-fluorouracil-resistant human colon cancer cells. Mol Cancer Ther. 2005;4:451–6.

1117. Zhu H, Hasman RA, Young KM, Kedersha NL, Lou H. U1 snRNP-dependent function of TIAR in the regulation of alternative RNA processing of the human calcitonin/CGRP pre-mRNA. Mol Cell Biol. 2003;23:5959–71.

1118. Zibetti C, Adamo A, Binda C, Forneris F, Toffolo E, Verpelli C, et al. Alternative splicing of the histone demethylase LSD1/KDM1 contributes to the modulation of neurite morphogenesis in the mammalian nervous system. J Neurosci. 2010;30:2521–32.

1119. Zmijewski MA, Slominski AT. Modulation of corticotropin releasing factor (CRF) signaling through receptor splicing in mouse pituitary cell line AtT-20--emerging role of soluble isoforms. J Physiol Pharmacol. 2009;60 Suppl 4:39–46.

1120. Zühlke RD, Bouron A, Soldatov NM, Reuter H. Ca2+ channel sensitivity towards the blocker isradipine is affected by alternative splicing of the human alpha1C subunit gene. FEBS Lett. 1998;427:220–4.

1121. Zunino G, Messina A, Sgadò P, Baj G, Casarosa S, Bozzi Y. Brain-derived neurotrophic factor signaling is altered in the forebrain of Engrailed-2 knockout mice. Neuroscience. 2016;324:252–61.

1122. COOH-terminal sequence motifs target the T cell protein tyrosine phosphatase to the ER and nucleus. J Cell Biol. 1995;131:631–43.
